# Dissecting the role of epigenetic regulation in oral squamous cell carcinoma microenvironment: mechanisms and therapeutics

**DOI:** 10.3389/fimmu.2026.1758433

**Published:** 2026-01-26

**Authors:** Xuechao Li, Yifei Ren, Shenghua Pei, Kai Zhao, Guanyu Chen, Zhenglin He

**Affiliations:** 1 School and Hospital of Stomatology, Jilin University, Changchun, China; 2 Department of Immunobiology, Yale University School of Medicine, New Haven, CT, United States; 3 Center of Molecular and Cellular Oncology, Yale Cancer Center, Yale University, New Haven, CT, United States; 4 Department of Experimental Orofacial Medicine, Marburg University, Marburg, Germany; 5 China-Japan Union Hospital of Jilin University, Jilin University, Changchun, China

**Keywords:** biomarkers, epigenetic regulation, oral squamous cell carcinoma, therapeutic targets, tumor microenvironment, DNA methylation, histone modification, drug resistance

## Abstract

Oral squamous cell carcinoma (OSCC) is a prevalent and aggressive malignancy with a persistently high mortality rate, largely attributable to therapy resistance and tumor recurrence. This review comprehensively explores the critical interplay between epigenetic dysregulation and the tumor microenvironment (TME) in driving OSCC progression. We detail how key epigenetic mechanisms, including DNA methylation, histone modifications, and non-coding RNAs (ncRNAs), intrinsically transform cancer cells and actively orchestrate pro-tumorigenic TME. These alterations substantially contribute to resistance against conventional therapies. Furthermore, we discuss the therapeutic potential of targeting these pathways using epigenetic drugs (epi-drugs), such as DNA methyltransferase (DNMT) inhibitors and histone deacetylase (HDAC) inhibitors, as well as engineered extracellular vesicles (EVs). The primary objective of this review is to synthesize current knowledge on the epigenetic-TME axis, thereby providing a mechanistic foundation for developing novel therapeutic strategies. We emphasize that rational combinations of epigenetic-targeting agents with conventional treatments or immunotherapy hold significant promise for overcoming drug resistance and improving clinical outcomes in OSCC patients.

## Introduction

Oral squamous cell carcinoma (OSCC) represents a significant global health burden, accounting for over 90% of all oral malignancies. It is among the most common cancers worldwide, with incidence rates varying geographically and influenced by regional risk factors (1, 2). Major risk factors include tobacco use, heavy alcohol consumption, betel quid chewing, and infection with high-risk human papillomavirus (HPV) types (1, 3, 4). Clinically, OSCC often presents as non-healing ulcers, erythroplakia or leukoplakia, pain, or dysphagia, and diagnosis typically involves clinical examination, imaging, and histopathological confirmation via biopsy (5–7). Current treatment strategies primarily encompass surgery, radiotherapy, and chemotherapy, tailored to tumor stage and patient condition (8). However, these approaches are frequently hampered by limitations such as locoregional recurrence, metastasis, and the development of drug resistance, which collectively contribute to poor five-year survival rates, particularly in advanced cases (8, 9). The emergence of resistance to conventional therapies like cisplatin and 5-fluorouracil underscores the urgent need for novel therapeutic targets and improved diagnostic biomarkers (8, 10, 11).

The limited efficacy of these conventional therapies and the frequent emergence of resistance are not solely attributable to the cancer cells themselves. This resistance is fueled by the dynamic and supportive tumor microenvironment (TME), which is now recognized as an essential contributor to OSCC progression (12). The complex interplay between cancer cells and the surrounding stromal and immune cells within the TME acts as a major driver of tumor growth, immune evasion, and the development of therapy resistance (13, 14). In recent years, epigenetic mechanisms have garnered substantial attention for their pivotal role in OSCC pathogenesis and progression (3, 15). Epigenetics refers to heritable changes in gene expression that do not involve alterations to the underlying DNA sequence, primarily encompassing three key mechanisms: DNA methylation, histone modifications, and regulation by non-coding RNAs (ncRNAs) (3, 15, 16). In OSCC, aberrant DNA methylation is a frequent event, characterized by global hypomethylation leading to genomic instability and oncogene activation, as well as promoter-specific hypermethylation resulting in the silencing of tumor suppressor genes (17, 18). Histone modifications, including acetylation, methylation, and phosphorylation, alter chromatin structure and accessibility, thereby modulating the expression of genes critical for cell cycle control, apoptosis, and invasion (19–21). Additionally, ncRNAs, particularly microRNAs (miRNAs) and long non-coding RNAs (lncRNAs), function as post-transcriptional regulators or epigenetic modulators, influencing various oncogenic pathways (22–24). These epigenetic alterations collectively drive OSCC initiation, progression, and metastasis by disrupting normal cellular processes, and they also contribute to therapy resistance mechanisms (25).

Given the pivotal roles of these epigenetic mechanisms in OSCC pathogenesis, their dynamic and reversible nature makes them attractive targets for therapeutic intervention (26, 27). Epigenetic-targeted therapies, such as DNA methyltransferase (DNMT) inhibitors and histone deacetylase (HDAC) inhibitors, have demonstrated potential in preclinical OSCC models by reversing aberrant gene silencing, thereby reactivating tumor suppressor genes or suppressing oncogenes, and restoring normal cellular functions (27–29). Beyond small molecule inhibitors, the field is exploring innovative delivery platforms for epigenetic therapy. In this context, extracellular vesicles (EVs), natural carriers of epigenetic regulators like miRNAs, are emerging as promising therapeutic tools (30). Leveraging this innate biology, engineered EVs represent a representative strategy for the precise delivery of epigenetic therapeutics (31).

**Figure 1:**
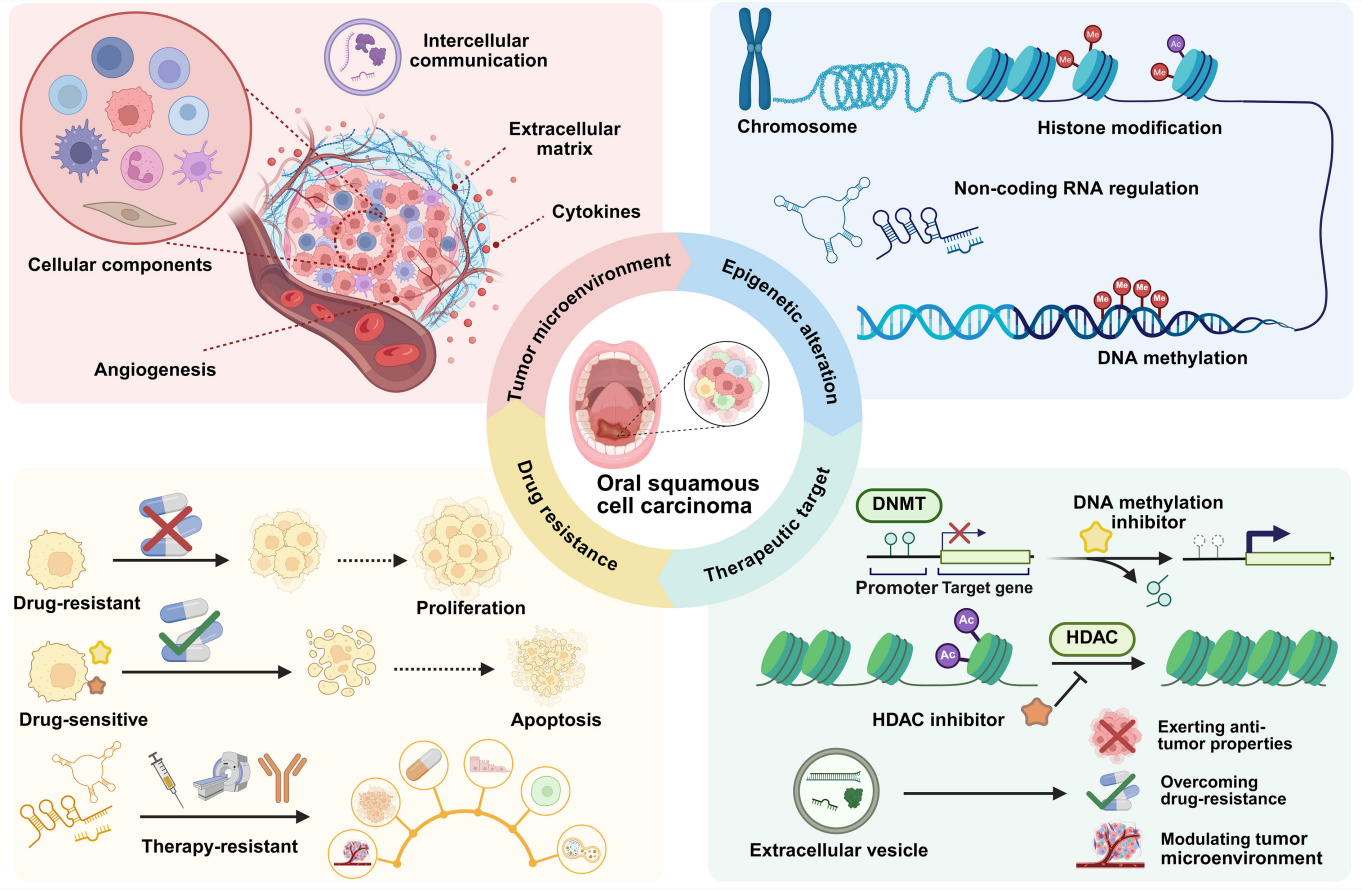
Schematic representations of roles of epigenetic regulation in OSCC. DNA methylation, histone modifications and ncRNAs regulate OSCC progression and pro-tumor TME remodeling, support therapeutic targets and mediate drug resistance. Abbreviations: DNMT: DNA methyltransferase; HDAC:histone deacetylase; TME: Tumor microenvironment.

Although the contributions of epigenetic alterations and the TME to OSCC pathogenesis are increasingly recognized, a key unknown is how their dynamic interplay, particularly the epigenetic orchestration of a pro-tumorigenic TME, contributes to therapy resistance. Therefore, this review aims to bridge this knowledge gap by comprehensively exploring the intricate interplay between epigenetic regulations and the TME of OSCC. We delve into the mechanisms by which DNA methylation, histone modifications, and ncRNAs not only drive intrinsic cancer cell pathways but also orchestrate a pro-tumorigenic TME. By integrating these insights, we further discuss the implications of these epigenetic mechanisms for developing novel therapeutic strategies, with a specific focus on overcoming drug resistance by targeting the epigenetic-TME axis, ultimately paving the way for more effective combination therapies in OSCC (**Figure 1**).

## 2 TME in OSCC

**Figure 2:**
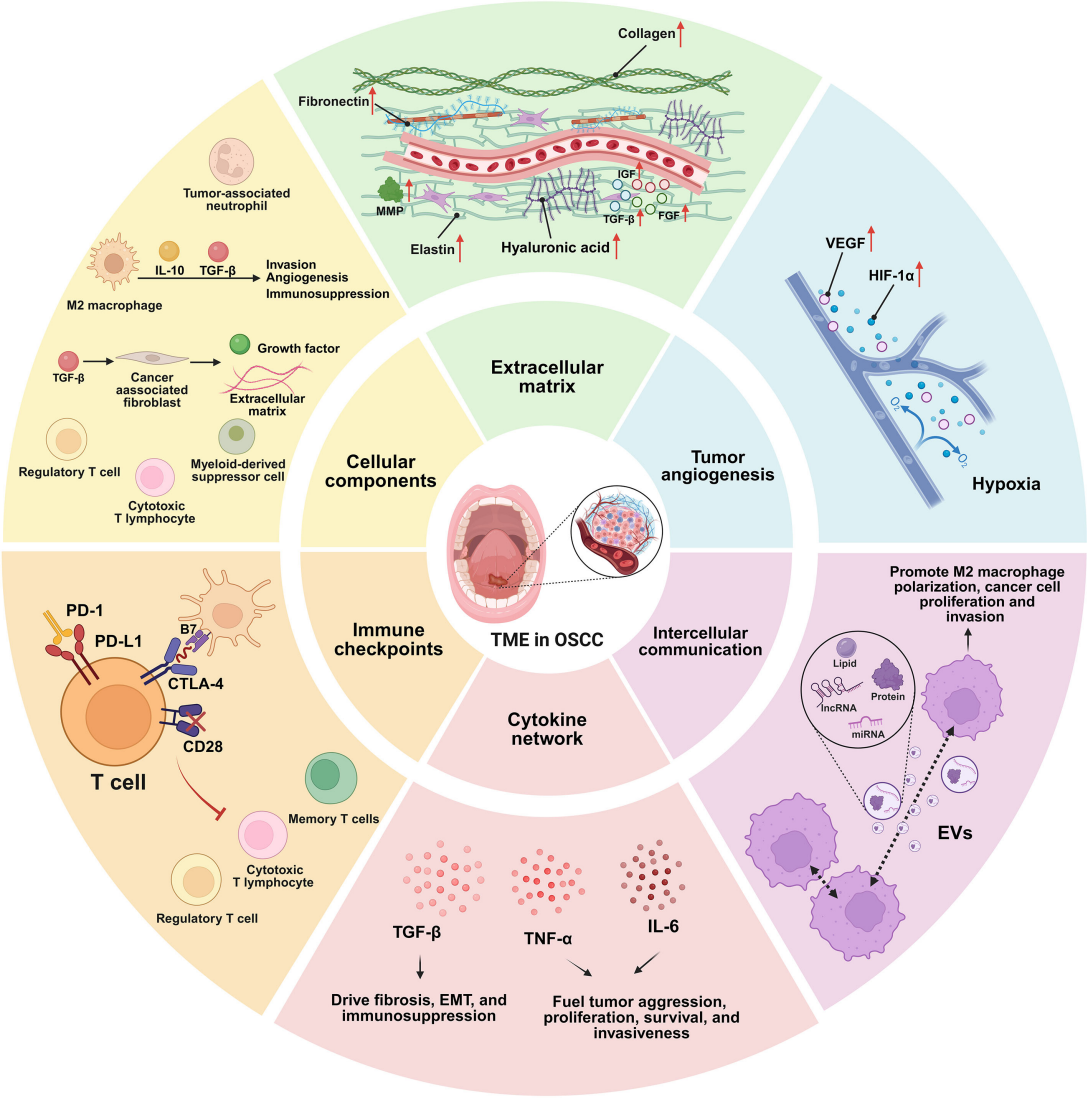
The TME of OSCC. The TME is a dynamic ecosystem critical for OSCC initiation, progression, and therapy resistance (33). It comprises diverse cellular and non-cellular components that interact to foster a pro-tumorigenic niche (34). Cellular components: Key residents include tumor-associated macrophages (TAMs), particularly the M2-polarized subtype recruited by C-C motif chemokine ligand 2 (CCL2) (35) and colony stimulating factor 1 (CSF-1) (35), which promote invasion, angiogenesis, and immunosuppression via interleukin 10 (IL-10) (36) and transforming growth factor beta (TGF-β) (37). Cancer-associated fibroblasts (CAFs), activated by tumor-derived TGF-β, remodel the stroma, secrete extracellular matrix (ECM) and growth factors, and support cancer stemness and therapy resistance (38, 39). The immune landscape includes cytotoxic T lymphocytes (CTLs) (40) whose function is suppressed, alongside immunosuppressive regulatory T cells (Tregs) (41) and myeloid-derived suppressor cells (MDSCs) (42) that inhibit effector T-cells and promote immune tolerance (41, 42). Extracellular matrix (ECM): The ECM provides structural support and biochemical signaling (43–45). It is composed of collagens (43), fibronectin (44), elastin (45), and hyaluronic acid (46). Remodeling by stromal cells, via enzymes like matrix metalloproteinases (MMPs) (47), releases sequestered growth factors (e.g., IGF, FGF, TGF-β), facilitating invasion, angiogenesis, and tumor progression (48). Tumor angiogenesis: Driven by hypoxia-inducible factor 1-alpha (HIF-1α) stabilization and subsequent VEGF overexpression, angiogenesis supplies nutrients and oxygen (49, 50). The resulting vasculature is often disorganized and leaky, hindering drug delivery and facilitating metastasis (51, 52). Intercellular communication: Crosstalk occurs via direct contact, secreted factors, and extracellular vesicles (EVs) such as exosomes (53, 54). Tumor and CAF-derived exosomes transfer proteins, lipids, and nucleic acids (e.g., lncRNAs, miRNAs) to reprogram recipient cells, promoting traits like M2 macrophage polarization or enhanced cancer cell proliferation and invasion (54–56). Cytokine network: A pathogenic network of cytokines sustains chronic inflammation and immunosuppression (48). TGF-β drives fibrosis, EMT, and immunosuppression (37). Pro-inflammatory cytokines like IL-6 (activating STAT3) (57) and tumor necrosis factor alpha (TNF-α) (58) directly fuel tumor aggression, proliferation, survival, and invasiveness. Immune checkpoints: OSCC cells exploit regulatory pathways like programmed death 1 (PD-1)/programmed death ligand 1 (PD-L1) and cytotoxic T-lymphocyte-associated protein 4 (CTLA-4) to evade immune destruction (59, 60). PD-L1 binding to PD-1 on T cells induces inhibitory signaling and exhaustion, while CTLA-4 competitively inhibits T-cell activation. Their upregulation is a key mechanism of immune resistance (60).

TME is a critical determinant in the initiation, progression, and metastasis of OSCC (12, 32). It is a complex and dynamically evolving ecosystem comprising various cellular and non-cellular components that interact dynamically to either suppress or, more commonly, promote tumorigenesis (**Figure 2**) (13). This evolution involves a shift from an initially pro-inflammatory state towards a profoundly immunosuppressive and pro-angiogenic landscape. Critically, the TME also plays a major role in treatment response by hindering drug delivery and suppressing anti-tumor immunity, thereby fostering therapy resistance (14). This pro-tumorigenic and therapy-resistant functionality is not a static feature, but is actively shaped and maintained through sustained signaling and intercellular crosstalk. Consequently, understanding the composition and function of the TME is essential for developing novel therapeutic strategies (10).

## 3 Epigenetic alterations in TME of OSCC

**Figure 3:**
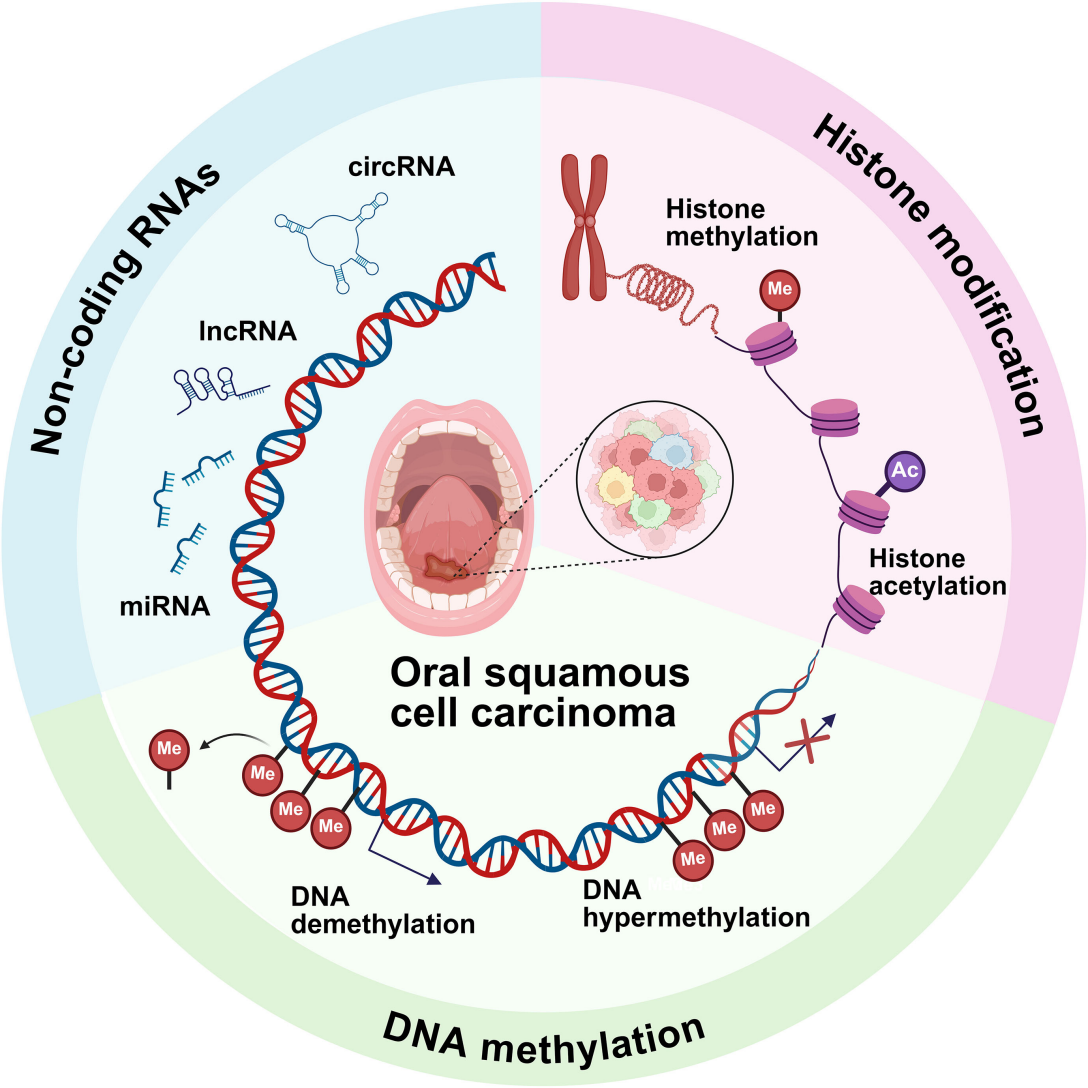
Key epigenetic alterations in OSCC. DNA methylation (hypomethylation/hypermethylation), histone modifications (methylation, acetylation), and ncRNA regulation (lncRNAs, miRNAs, circRNAs) drive OSCC pathogenesis and TME remodeling.

The progression of OSCC is significantly driven by epigenetic dysregulation, encompassing aberrant DNA methylation, histone modifications, and ncRNA function (**Figure 3**) (3). These mechanisms collectively orchestrate key oncogenic processes by modulating genes critical to DNA repair, cell cycle control, apoptotic signaling, and metastatic dissemination (15). Importantly, growing evidence indicates that epigenetic alterations are pivotal in reshaping the TME and modulating anti-tumor immunity, thereby influencing tumor progression and therapy response (61). Consequently, targeting these epigenetic drivers presents a promising avenue for developing innovative diagnostic biomarkers and therapeutic interventions, offering the potential to enhance clinical management for OSCC patients.

### 3.1 DNA methylation in OSCC

**Figure 4:**
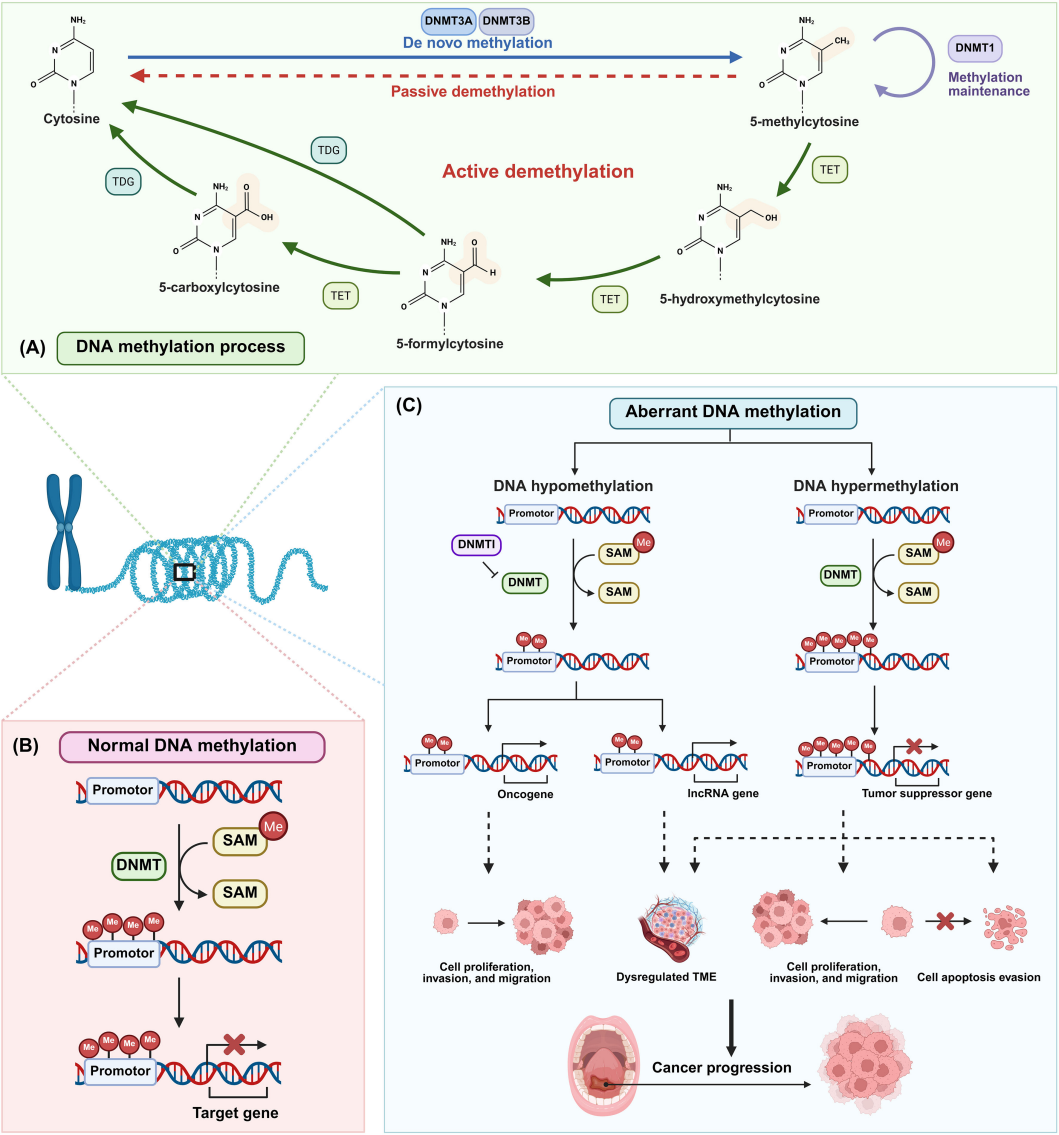
Normal and aberrant DNA methylation in OSCC. **(A)** DNA methylation process: *De novo* methylation establishes new DNA methylation patterns during early development. Demethylation removes methyl groups from the DNA molecule. **(B)** Normal DNA methylation: This process is catalyzed by DNMT and needs methyl provided by SAM. Promoter methylation typically leads to transcriptional silencing, effectively turning genes off. **(C)** Aberrant DNA methylation: Hypermethylation of tumor suppressor gene promoters leads to their silencing, impairing their ability to regulate cell growth and suppress tumor progression. Conversely, hypomethylation of oncogene promoters activates these genes, promoting abnormal cell growth and tumor progression. Abbreviations: DNMT: DNA methyltransferase; DNMTI: DNA methyltransferase inhibitor; SAM: S-Adenosylmethionine; TET: Ten-eleven translocation methylcytosine dioxygenase; TDG: Thymine-DNA glycosylase.

DNA methylation is a fundamental epigenetic modification involving the addition of a methyl group to a cytosine base in DNA, primarily at CpG sites (62). This process is catalyzed by DNMTs, which establish and maintain these methylation patterns through cell division (63). The primary functional consequence of DNA methylation is the regulation of gene expression (**Figure 4**) (64). Promoter hypermethylation typically leads to transcriptional silencing, effectively turning genes off (65). In contrast, global hypomethylation can result in genomic instability and the inappropriate activation of genes (66).

However, in the neoplastic context of OSCC, this precise regulation is profoundly disrupted. The genome undergoes widespread hypomethylation, promoting genomic instability, while specific promoter-associated CpG islands become subject to hypermethylation, leading to the transcriptional silencing of critical tumor suppressor genes (3). This dual dysregulation is a hallmark of OSCC, fundamentally contributing to its initiation and malignant progression (67). Critically, both hypermethylation and hypomethylation events can alter the expression of immunomodulatory genes and cancer-associated signaling pathways, actively sculpting the immunosuppressive TME (**Figure 4**) (3).

#### 3.1.1 Hypomethylated genes involving OSCC

In OSCC, DNA hypomethylation drives tumorigenesis by activating oncogenes and pro-metastatic factors, dysregulating key signaling pathways, and remodeling the TME (17). The following sections detail the roles of specific hypomethylated genes in these processes.

Dysregulated DNA hypomethylation plays a crucial role in reshaping the immunosuppressive TME (68). A key mechanism involves tumor-derived factors that actively reprogram the epigenome of immune cells. For instance, calnexin, an endoplasmic reticulum chaperone upregulated in OSCC, is expressed on the tumor cell membrane. It impairs antitumor immunity by interacting with T cells and inducing DNA hypomethylation of the programmed cell death protein 1 (PD-1) promoter CpG island. This epigenetic alteration leads to increased PD-1 expression on CD4^+^ and CD8^+^ T cells, driving their functional exhaustion and suppressing cytokine production. Consequently, calnexin-expressing tumors exhibit reduced T-cell infiltration and poorer patient survival, illustrating how a tumor cell surface protein can exploit DNA demethylation to enforce an immunosuppressive TME (69).

A primary oncogenic effect of DNA hypomethylation is the transcriptional reactivation of genes that drive tumor aggression and metastasis. For instance, the promoter of Wnt1 inducible signaling pathway protein 1 (WISP1) is significantly hypomethylated in OSCCs with lymph node metastasis, leading to its high expression which promotes cancer spread and correlates with poorer survival (70). Similarly, hypomethylation at the homeobox protein CDX-1 (CDX1) motif reactivates the transcription factor gene homeobox C9 (HOXC9) in OSCC. HOXC9 upregulation promotes tumor invasion and metastasis by driving the expression of MMP13 through the ITGA6/PI3K/Akt signaling axis and is associated with advanced disease stages (71). However, the proposed HOXC9-driven PI3K-Akt/MMP13 axis represents a potential pathological association requiring further direct validation, and the regulatory interplay between HOXC9 and upstream factors remains to be fully elucidated.

The activation of pivotal oncogenic signaling pathways is another major consequence of gene-specific hypomethylation. For instance, in higher-grade OSCC samples, the dickkopf Wnt signaling pathway inhibitor 2 (DKK2) and DKK4 genes, which are inhibitors of the Wnt signaling pathway, were found to be hypomethylated. This hypomethylation is postulated to lead to their increased expression, potentially facilitating tumor cell invasion and progression through the modulation of the Wnt pathway, a key driver in oral carcinogenesis (72). Furthermore, in OSCC associated with oral lichen planus, promoter hypomethylation drives the overexpression of SRY-box transcription factor 11 (Sox11), which in turn activates the PI3K/AKT signaling pathway and enhances glycolysis to promote tumor growth (73). Integrated bioinformatic analyses corroborate that numerous hypomethylated and upregulated genes in OSCC are key components of the PI3K/AKT and epithelial-mesenchymal transition (EMT) pathways (74). Therefore, identifying additional oncogenic pathways activated by this mechanism remains a crucial goal for future research.

Beyond the regulation of individual genes, widespread hypomethylation exerts a profound influence on the TME. Global DNA hypomethylation, measurable through long interspersed element 1 (LINE-1) repetitive elements, is a common feature of OSCC, and low LINE-1 methylation levels in pre-malignant lesions predict a higher risk of progression to cancer (75). This hypomethylated state extends to immune-related genes. Genome-wide studies have identified unique sets of hypomethylated promoters enriched for immune response genes, suggesting that DNA hypomethylation can facilitate lymphocyte infiltration and modulate anti-tumor immunity (76). This is supported by findings that promoters of various immune genes are significantly more hypomethylated in OSCC tissues compared to normal mucosa, irrespective of HPV status (77).

The consistent pattern of DNA hypomethylation in OSCC offers considerable potential for clinical translation into biomarkers (78). Studies on oral brushing samples have identified hypomethylated genes like miR-296 and telomerase reverse transcriptase (TERT) in OSCC and pre-malignant lesions, demonstrating their utility for early and non-invasive detection (79). In mouse models, hypomethylation and overexpression of Fgf3 occur during the early stages of oral carcinogenesis, marking it as a potential early detection biomarker (80). From a prognostic perspective, hypomethylation of the ornithine aminotransferase (OAT) gene promoter is associated with a radio-resistant TME and poorer survival after radiotherapy (81), whereas the hypomethylated state of the tubulin polymerization promoting protein family member 3 (TPPP3) promoter, which favors its tumor-suppressive expression, is an indicator of good prognosis (82). The lncRNA H19 also exhibits promoter hypomethylation and high expression in OSCC, which is associated with a significantly lower 5-year survival rate, underscoring its role in disease progression (83). It promotes disease progression through diverse mechanisms, including acting as a competitive endogenous RNA (ceRNA) to sponge miRNAs such as miR-138, miR-29b, and let-7a. This sponge activity leads to the upregulation of downstream targets like zeste homolog 2 (EZH2), zinc finger E-box binding homeobox 1 (ZEB1), and 6-phosphofructo-2-kinase/fructose-2,6-biphosphatase 3 (PFKFB3), thereby driving EMT, enhancing cell proliferation, invasion, and glycolysis, and contributing to immunosuppressive TME (84).

In conclusion, DNA hypomethylation is a pervasive and driving force in OSCC pathogenesis. It functions not only by activating specific oncogenes and pathways that enhance tumor cell proliferation, invasion, and metastasis, but also by reshaping the global genomic and immune landscape, most notably by reshaping immunosuppressive TME, to ultimately favor tumor survival and progression. The wealth of hypomethylated genes and loci, many with clear clinical correlations, positions DNA hypomethylation as a rich source of mechanistic insights and a promising foundation for developing diagnostic, prognostic, and therapeutic strategies against OSCC.

#### 3.1.2 Hypermethylated genes involving OSCC

DNA hypermethylation of tumor suppressor genes is a fundamental epigenetic mechanism driving OSCC pathogenesis (85). A key consequence of this silencing is the blunting of anti-tumor immune responses and the fostering of immunosuppressive TME, which is increasingly recognized as a critical step in OSCC progression (86). Recent studies have identified a range of genes silenced by this mechanism, which can be broadly grouped by their disrupted cellular functions, underscoring the critical role of epigenetic dysregulation in OSCC.

A primary mechanism by which promoter hypermethylation directly establishes immunosuppressive TME in OSCC is the silencing of genes that regulate key immune checkpoint molecules (87). The paired box 1 (PAX1) gene is downregulated due to arecoline-induced hypermethylation. This loss of PAX1 function is a pivotal event that not only enhances cancer stem cell (CSC) -like properties but also actively promotes an immunosuppressive TME by upregulating interferon induced protein with tetratricopeptide repeats 1 (IFIT1) and the immune checkpoint programmed death ligand 1 (PD-L1), thereby facilitating tumor immune evasion. Future validation in an orthotopic animal model is needed to confirm the involvement of the IFIT1/PD-L1 signaling pathway (88). Similarly, promoter hypermethylation-induced downregulation of the tumor suppressor miRNA miR-34b/c drives OSCC aggressiveness and is a marker of adverse clinical outcomes, including shortened survival (89). Given the established role of the miR-34 family in directly targeting and suppressing immune checkpoints such as PD-L1 in other cancers (90), its silencing in OSCC likely contributes directly to immune dysregulation within the TME. This epigenetic event is strongly associated with advanced tumor stage, nodal metastasis, cancer recurrence, and poor survival outcomes particularly in HPV-negative OSCC patients (89). The silencing of PAX1 and miR-34b/c exemplifies how hypermethylation can be leveraged by OSCC to directly suppress anti-tumor immunity.

A significant group of hypermethylated genes disrupts apoptosis and cell proliferation, which can indirectly alter immune surveillance. The pro-apoptotic gene homeobox A5 (HOXA5) is frequently hypermethylated in OSCC tissues leading to its downregulation, contributing to reduced cell death. Reactivation of HOXA5 expression not only induces cell death but also enhances chemosensitivity both *in vitro* and *in vivo* (91). Another key gene, PAX9, a differentiation-associated tumor suppressor, is silenced through hypermethylation. Pharmacological inhibition of DNMTs can reactivate PAX9 expression, which in turn triggers apoptosis, inhibits cell growth, and suppresses cancer stemness through an autophagy-dependent pathway (92). The evasion of apoptosis and enhanced stemness mediated by the silencing of these genes contribute to a tumor cell population that is resistant to immune cell-mediated killing and fosters a pro-tumorigenic niche.

Another critical pathway affected by hypermethylation involves genes that control cell invasion, metastasis, and stemness. Methylation-mediated silencing of miR-124–3 represents one mechanism. The suppression of this miRNA leads to the overexpression of its target, the oncogene leucine rich repeat containing 1 (LRRC1), which subsequently drives OSCC cell proliferation and migration (93). The homeobox gene HOXA3 also exhibits epigenetic regulation, with its expression showing an inverse correlation with promoter methylation levels. Hypermethylation of its 3’ untranslated region is notably associated with poor overall survival in advanced-stage OSCC patients (94). Moreover, HOXA3 expression may also be subject to post-transcriptional regulation by ncRNAs and RNA-binding proteins, although this complex regulatory network requires definitive experimental confirmation. Furthermore, the tumor suppressor gene transglutaminase 3 (TGM-3) shows significant promoter hypermethylation in OSCC. This epigenetic alteration is quantitatively associated with advanced tumor stage and higher histological grade, highlighting its role in cancer progression (95). The concerted hypermethylation of this group of genes thus equips OSCC cells with enhanced invasive, metastatic, and stem-like capabilities, all of which are hallmarks of an aggressive TME that supports tumor dissemination and therapy resistance.

Beyond the silencing of protein-coding genes and miRNAs, hypermethylation also affects genes that regulate the epigenetic machinery itself. The methylation status of DNMT3A and tet methylcytosine dioxygenase 2 (TET2), a key demethylation-initiating enzyme, is aberrant in OSCC. Functionally, knockdown of DNMT3A and overexpression of TET2 can inhibit the proliferation and migration of OSCC cells, indicating their crucial roles in maintaining epigenetic balance (96). On a different level, the protein methyltransferase SET domain containing 6 (SETD6) is upregulated in OSCC. Silencing SETD6 inhibits OSCC tumorigenesis by reducing the promoter methylation of its substrate proteins P21 (RAC1) activated kinase 4 (PAK4) and RELA proto-oncogene, NF-κB subunit gene (RelA), thereby counteracting their pro-tumorigenic activities (97). This category reveals a self-reinforcing loop in OSCC, where the epigenetic regulators themselves become targets of dysregulation, further amplifying the global epigenetic disruption that underpins a malignant and immunosuppressive TME.

In summary, the landscape of hypermethylated genes in OSCC is diverse, encompassing key regulators of apoptosis, invasion, stemness, drug resistance, and the epigenetic apparatus itself, which significantly reshapes the TME of OSCC (**Table 1**). The collective evidence from these studies strongly supports the potential of these hypermethylated genes as valuable biomarkers for diagnosis and prognosis, as well as promising targets for epigenetic therapy in OSCC.

**Table 1:** The role of DNA methylation in OSCC onset and progression.

Methylation regulation	Gene	Locus	Function	Ref.
Hypomethylated genes	PD-1	2q37.3	The hypomethylation of the PD-1 promoter CpG island upregulates PD-1 expression on T cells, thereby driving T cell exhaustion and enforcing immunosuppressive TME.	(69)
	WISP1	8q24.22	Promoter of WISP1 leads to lymph node metastasis and correlates with poorer survival.	(70)
	CDX1	5q32	Hypomethylation at the CDX1 motif reactivates HOXC9 transcription, thereby promoting tumor invasion and metastasis via the ITGA6/PI3K/Akt/MMP13 axis.	(71)
	lncRNA H19	11p15.5	Promoter hypomethylation of lncRNA H19 contributes to TME dysregulation and is associated with a significantly lower 5-year survival rate.	(83)
	DKK2/4	4q25/ 8p11.21	DKK2/4 hypomethylation facilitates tumor cell invasion and progression.	(72)
	Sox11	2p25.2	Promoter hypomethylation of Sox11 enhances glycolysis to promote tumor cell proliferation, invasion, and migration.	(73)
	LINE-1	Widespread	Low LINE-1 methylation levels in pre-malignant lesions predict a higher risk of progression to cancer	(75)
	miR-296	20q13.32	Detects for early and non-invasive detection.	(79)
	TERT	5p15.33		(79)
	Fgf3	11q13.3	Hypomethylation and overexpression of Fgf3 function as a potential early biomarker for OSCC detection.	(80)
	OAT	10q26.13	Hypomethylation of the OAT promoter is associated with a radio-resistant TME and poorer survival after radiotherapy	(81)
	TPPP3	16q22.1	Hypomethylation of TPPP3 promoter favors its tumor-suppressive expression.	(82)
	TET2	4q24	TET2 hypomethylation suppresses OSCC progression.	(96)
Hypermethylated genes	PAX1	20p11.22	PAX1 hypermethylation promotes cancer stemness and immunosuppressive TME.	(88)
	miR-34b/c	11q23.1	Hypermethylation of miR-34b/c promoter promotes immune dysregulation within the TME.	(89)
	HOXA5	7p15.2	Hypermethylation and downregulation of HOXA5 Suppresses cell death and confers cisplatin resistance in OSCC.	(91)
	PAX9	14q13.3	PAX9 hypermethylation promotes evasion of apoptosis and enhances cancer stemness.	(92)
	miR-124-3	20q13.3	Hypomethylation of miR-124-3 leads to the overexpression of LRRC1, driving OSCC cell proliferation and migration.	(93)
	HOXA3	7p15.2	HOXA3 hypermethylation is associated with poor overall survival in advanced-stage OSCC patients.	(94)
	TGM-3	20q11.23	Promoter hypermethylation of TGM-3 is associated with advanced tumor stage and higher histological grade	(95)
	DNMT3A	2p23.3	Knockdown of DNMT3A promotes OSCC cell proliferation and migration.	(96)
	PAK4	19q13.2	Promoter hypermethylation of PAK4 and RelA enhances its pro-tumorigenic activities and promotes OSCC tumorigenesis.	(97)
	RelA	11q13.1		

PD-1, Programmed cell death 1; WISP1, WNT1-inducible signaling pathway protein 1; TME, Tumor microenvironment; DKK2/4, Dickkopf WNT signaling pathway inhibitor 2/4; Sox11, SRY-box transcription factor 11; LINE-1, Long interspersed nuclear element-1; TERT, Telomerase reverse transcriptase; Fgf3, Fibroblast growth factor 3; OAT, Ornithine aminotransferase; TPPP3, Tubulin polymerization promoting protein 3; TET2, Ten-eleven translocation 2; PAX1, Paired box gene 1; HOXA5, Homeobox A5; LRRC1, Leucine rich repeat containing 1; TGM-3, Transglutaminase 3; DNMT3A, DNA methyltransferase 3 alpha; PAK4, P21-activated kinase 4; RelA, Reticuloendotheliosis viral oncogene homolog A.

### 3.2 Histone modifications in OSCC

**Figure 5:**
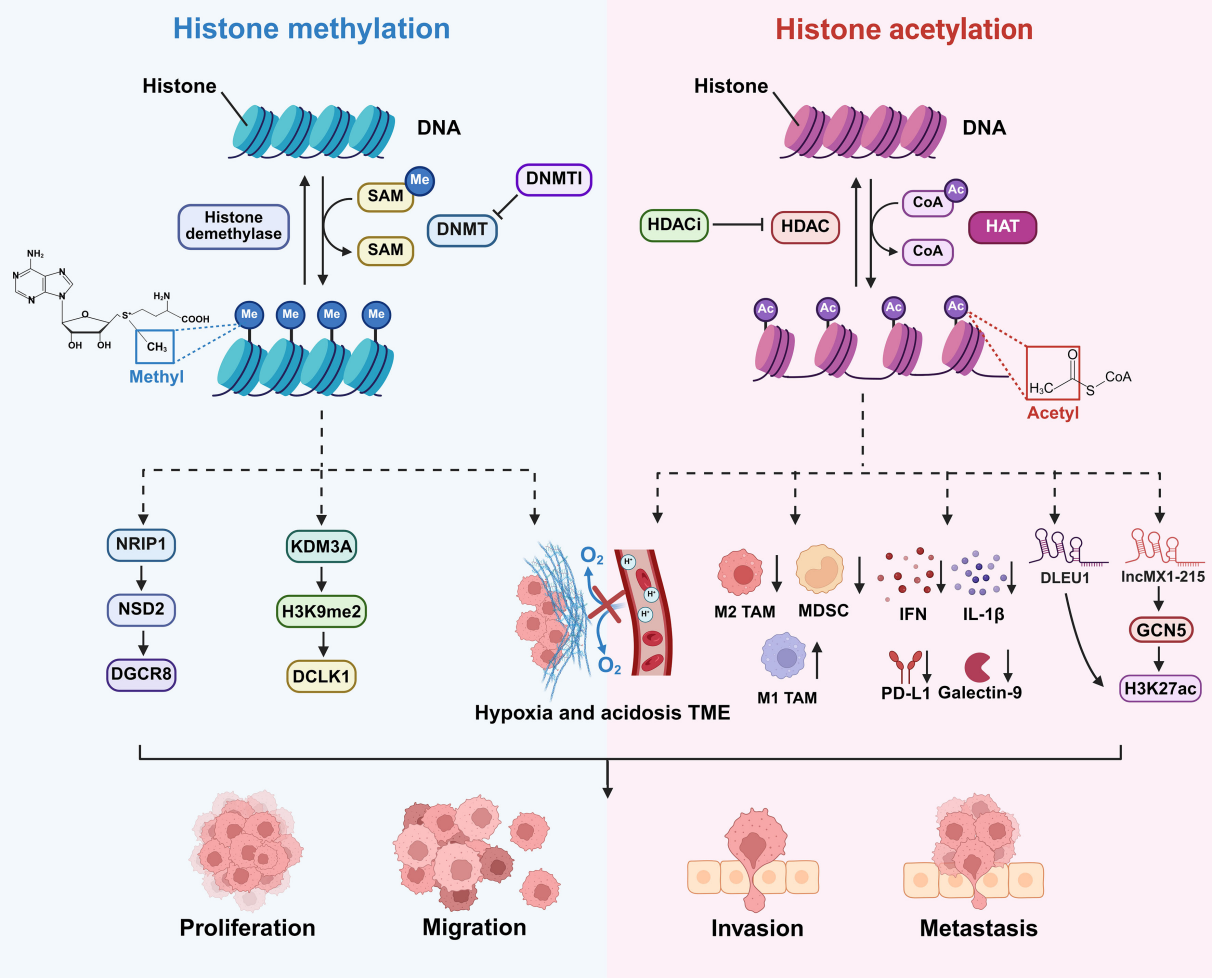
Histone modifications and their roles in OSCC progression. Histone methylation and acetylation drive OSCC progression via: NRIP1/NSD2/DGCR8 axis, KDM3A/H3K9me2/DCLK1 axis, TME hypoxia/acidosis, immunosuppressive molecules reduction and lncMX1-215/GCN5/H3K27ac axis, leading to tumor cell proliferation, migration, invasion, and metastasis. Abbreviations: SAM: S-Adenosylmethionine; DNMT: DNA methyltransferase; DNMTI: DNA methyltransferase inhibitor; HAT: Histone acetyltransferase; HDAC: histone deacetylase; NRIP1: Nuclear receptor interacting protein 1; NSD2: Nuclear receptor binding SET domain protein 2; DGCR8: DiGeorge syndrome chromosomal region 8; TME: Tumor microenvironment; TAM: Tumor-associated macrophage; MDSC: Myeloid-derived suppressor cell; IFN: Interferon; IL-1β: Interleukin-1beta; GCN5: General control non-repressed 5; H3K27ac: Histone 3 K lysine 27 acetylation.

Histone modifications represent a fundamental layer of epigenetic regulation that controls chromatin architecture and gene expression without altering the underlying DNA sequence (98). These chemical alterations including acetylation methylation phosphorylation and ubiquitination occur on the N-terminal tails of histone proteins (99). They are dynamically orchestrated by specific writer and eraser enzymes which add or remove these marks respectively. In OSCC, the precise balance of histone modifications is profoundly disrupted, leading to the aberrant silencing of tumor suppressor genes and the inappropriate activation of oncogenic pathways (100). Critically, emerging evidence indicates that histone modifications also govern the expression of immunomodulatory genes within cancer cells and stromal cells thereby actively shaping an immunosuppressive TME that facilitates immune evasion and disease progression (**Figure 5**) (101). Among the various types of histone modifications, methylation and acetylation are the most extensively studied in OSCC and will be the focus of the following sections.

#### 3.2.1 Histone methylation in OSCC

Histone methylation, a dynamic process regulated by methyltransferases and demethylases, plays a pivotal role in OSCC pathogenesis (102). Beyond driving cell-autonomous malignant behaviors, emerging evidence underscores its profound impact on shaping the TME, particularly in fostering immunosuppression and therapeutic resistance (103). The dysregulation of this epigenetic mechanism directly influences cellular responses to TME stressors and activates pathways that collectively mold a pro-tumorigenic niche (104).

A primary mechanism through which histone methylation influences OSCC involves direct modulation of the hypoxic and acidotic TME, a key driver of immunosuppression (105). This TME in OSCC, primarily regulated by hypoxia-inducible factor 1-alpha (HIF-1α), impacts the activity of histone-modifying enzymes, thereby reshaping the histone modification landscape. A foundational study utilizing LC-MS-based proteomics directly demonstrated that hypoxic, acidotic, and combined stress conditions in the CAL27 OSCC cell line induce distinct, position-dependent alterations in histone methylation and acetylation marks, such as histone H3 trimethylation at Lys36 (H3K36me3), histone H2A lysine 9 acetylation (H2AK9Ac), and H4K16Ac (105). Building upon this, a subsequent mass spectrometry-based proteomic study revealed that the combination of the HDAC inhibitor vorinostat and the thioredoxin-1 (Trx-1) inhibitor PX-12 further alters a spectrum of histone methylation and acetylation marks under hypoxic conditions (106). These findings position histone methylation as a crucial epigenetic interface between the hypoxic and acidotic TME and cancer cell adaptability, suggesting that targeting these modifications may reverse TME-mediated therapy resistance.

Beyond the direct hypoxic response, histone methylation fuels OSCC progression by activating specific oncogenic axes that enhance tumor aggressiveness and indirectly reshape the TME. A compelling signaling cascade involves the nuclear receptor interacting protein 1 (NRIP1)/nuclear receptor binding SET domain protein 2 (NSD2)/DiGeorge critical region 8 (DGCR8) axis. NRIP1, an aberrantly expressed transcription factor, activates the transcription of the methyltransferase NSD2, which in turn increases DGCR8 transcription by modulating histone methylation near its promoter. This axis significantly augments OSCC cell proliferation, migration, invasion, and *in vivo* metastatic potential (107). However, a key limitation of this work is that the critical downstream effectors of DGCR8, which are most likely specific miRNAs or signaling pathways, need to be further elucidated. Similarly, lysine demethylase 3A (KDM3A) facilitates OSCC proliferation and invasion by removing the repressive H3K9me2 mark from the promoter of doublecortin like kinase 1 (DCLK1), a CSC marker, thereby upregulating its expression (108). However, this established link is likely to represent a part of a broader regulatory network. A critical next step is to investigate the full spectrum of transcription factors targeted by KDM3A and to unravel the downstream signaling mechanisms through which DCLK1 executes its oncogenic functions. By promoting this stem-like phenotype, the KDM3A-H3K9me2-DCLK1 axis indirectly sustains the immunosuppressive TME, a consequence of the well-documented immunomodulatory and therapy-resistant properties of CSCs.

Specific histone methylation marks have clinical and prognostic significance in OSCC, indicating their role in the TME. Immunohistochemical analyses have consistently linked aberrant histone methylation marks to aggressive disease and poor survival. The upregulation of the heterochromatin mark H3K9me3 in OSCC tissues is associated with advanced disease features like depth of invasion (109). Future studies should dissect the functional relationship between these epigenetic marks and key oncogenic pathways, particularly the poorly explored link with PI3K/AKT signaling in OSCC. Furthermore, high levels of the repressive mark H3K27me3, either alone or in combination with the active mark H3K27ac, are powerful predictors of shorter survival in OSCC patients (110). The prognostic value of these marks underscores their potential as biomarkers and therapeutic targets.

In summary, histone methylation drives OSCC progression through dual mechanisms: directly by modulating cellular responses to the TME, such as hypoxia, and indirectly by activating oncogenic pathways that remodel the tumor stroma. Key marks like H3K9me3 and H3K27me3 are linked to poor prognosis and likely foster an immunosuppressive TME. Targeting these epigenetic regulators presents a promising strategy to simultaneously curb tumor growth and counteract immunosuppression in OSCC.

#### 3.2.2 Histone acetylation in OSCC

Histone acetylation serves as a critical epigenetic mechanism in OSCC pathogenesis, with its dysregulation extending beyond cell-autonomous effects to actively shape the TME and immune response (111). Evidence increasingly shows that targeting acetylation pathways can directly reverse immunosuppressive networks and overcome therapy resistance.

Epigenetic modulation of histone acetylation can directly reshape the immune landscape of the TME (112). Pharmacological inhibition of HDAC6 by tubastatin A (TSA) presents a compelling case. It suppresses the secretion of the pro-tumorigenic cytokine interleukin 1β (IL-1β) and concurrently reprograms the immune landscape. This is evidenced by a reduction in myeloid-derived suppressor cells (MDSCs) and M2-type tumor-associated macrophages (TAM) alongside an increase in M1-type TAM, effectively alleviating immunosuppression (113). Furthermore, the oncogenic lncRNA deleted in lymphocytic leukemia 1 (DLEU1) influences immune signaling by modulating the active enhancer mark H3K27ac. DLEU1 knockdown reduces H3K27ac levels and suppresses interferon-stimulated genes, linking this specific acetylation mark to the regulation of JAK/STAT signaling and TME-associated immune responses (114). However, the mechanism behind the concomitant downregulation of genes unrelated to interferon signaling remains an open question, hinting at a broader epigenetic function for DLEU1 beyond the JAK/STAT pathway.

The regulatory network of histone acetylation involves complex interactions with lncRNAs, which can act as critical modulators of specific histone marks (115). As mentioned previously, the lncRNA DLEU1 contributes to OSCC by maintaining H3K27ac levels to activate oncogenic transcriptional programs (114). Conversely, a separate study in head and neck squamous cell carcinoma identified the interferon-alpha-induced lncRNA lncMX1-215, which negatively regulates immunosuppression. This lncRNA exerts its function by directly binding to the histone acetyltransferase general control non-depressible 5 (GCN5), a known writer of the H3K27ac mark. This interaction interrupts the binding of GCN5 to H3K27ac sites on the promoters of immunosuppressive molecules such as PD-L1 and galectin-9, thereby inhibiting their transcription (116). These findings demonstrate that lncRNAs can directly interface with the histone acetylation machinery, either by facilitating or by obstructing the deposition of specific acetyl marks to control gene expression programs in OSCC.

The clinical significance of these mechanisms is underscored by specific acetyl marks serving as robust prognostic biomarkers. Hyperacetylation of H3K18 and H3K9 is linked to advanced invasion and high T stage (109), while elevated H3K27ac levels are associated with shorter patient survival (110). These marks epitomize the aggressive, TME-shaped tumor phenotype. Notably, the expression and therapeutic response may exhibit sex-related differences, as female mice showed distinct dynamics of H3K9ac and H3K14ac during carcinogenesis (117), pointing to personalized therapeutic considerations.

Evidence indicates histone methylation and acetylation interact closely in OSCC (110). The lysine methyltransferase 2D (KMT2D), which catalyzes H3K4me1, also promotes H3K27ac enrichment at enhancers of key oncogenes like KLF transcription factor 7 (KLF7), activating their transcription (118). This shows one histone modifying enzyme can directly influence the deposition of another mark. This dynamic crosstalk represents a key epigenetic feature of OSCC, suggesting combination therapies targeting this network may be more effective than single agents.

In summary, histone acetylation is crucially implicated in molding the immunosuppressive TME of OSCC. It operates by directly reprogramming immune cells, mediating adaptation to TME stressors, and fueling the chemo-resistant CSC niche. The potent anti-tumor and immune-modulating effects of HDAC inhibitors, evidenced by both preclinical and clinical investigations (119), solidifying the targeting of acetylation pathways as a promising strategy to disrupt the pro-tumorigenic TME and improve OSCC treatment outcomes.

### 3.3 ncRNAs in OSCC

ncRNAs constitute a major category of functional transcripts that govern gene expression and cellular functions without encoding proteins (120). Key ncRNAs such as miRNAs, lncRNAs, and circRNAs are integral to the molecular circuitry of OSCC (121). Their dysregulation directly contributes to tumor development and progression (122, 123). Critically, ncRNAs exert profound influence over the composition and function of the TME, including the modulation of immune cell activity and stromal interactions. These molecules are central to establishing an immunosuppressive milieu that facilitates immune evasion and tumor persistence (124). The subsequent sections will explore mechanisms by which specific ncRNAs drive these processes within the TME of OSCC.

#### 3.3.1 lncRNAs in OSCC

**Figure 6:**
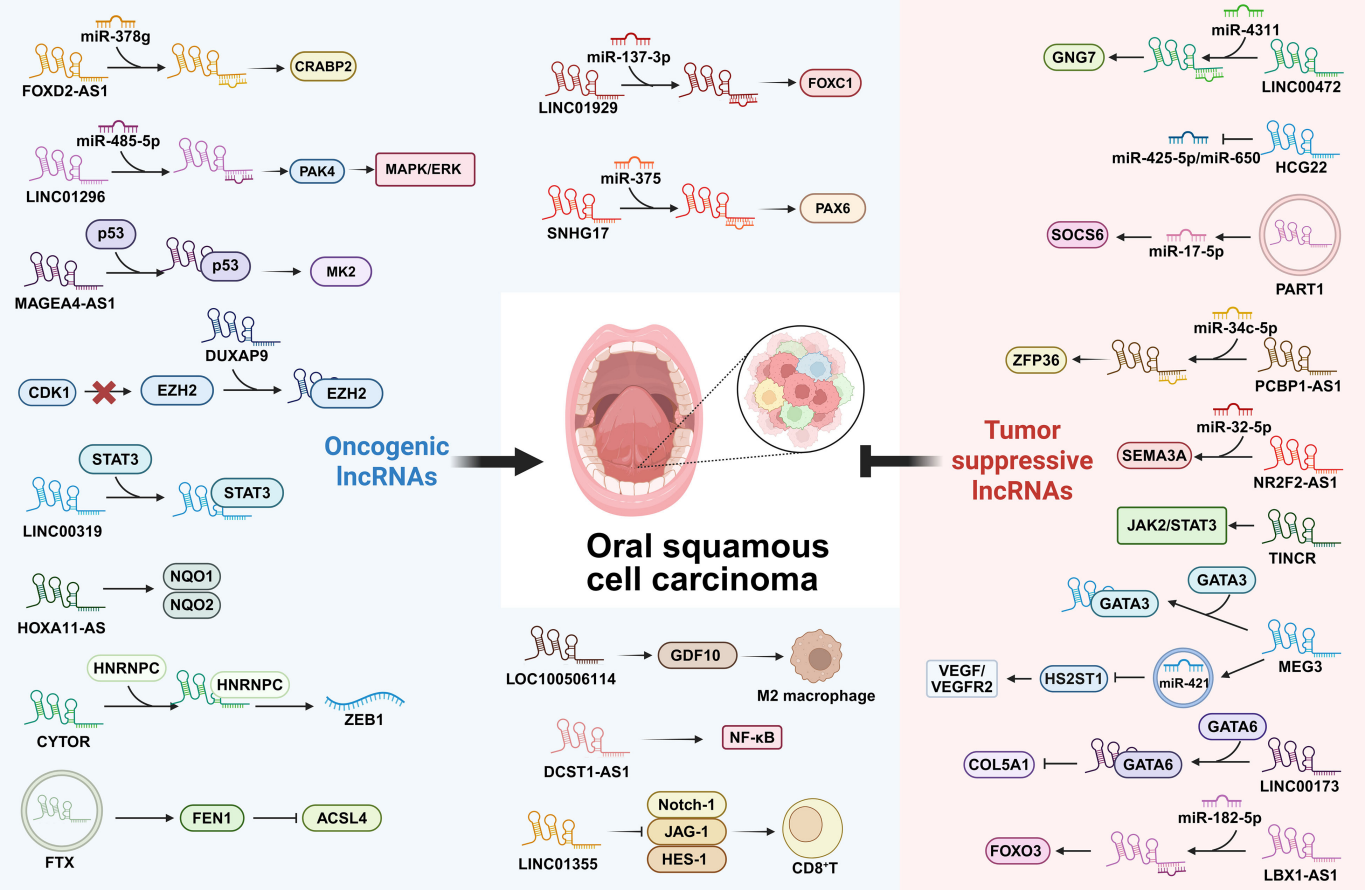
Regulatory network of long non-coding RNAs (lncRNAs) in OSCC. lncRNAs interact through multiple mechanisms, such as the competitive endogenous RNA network where lncRNAs sponge miRNAs to coordinately regulate gene expression. They exert oncogenic or tumor suppressive effects on tumor cell proliferation, apoptosis, invasion and stemness, and dynamically remodel the tumor microenvironment (TME).

lncRNAs are a class of transcripts longer than 200 nucleotides with limited or no protein-coding potential (125). They have emerged as critical regulators of gene expression at epigenetic, transcriptional, and post-transcriptional levels (126). In cancer, lncRNAs play pivotal roles in diverse biological processes, including cell proliferation, apoptosis, metastasis, and immune responses, functioning either as oncogenes or tumor suppressors (127, 128). In the context of OSCC, numerous lncRNAs have been identified to be dysregulated and contribute significantly to tumor initiation and progression (**Figure 6**).

##### 3.3.1.1 Oncogenic lncRNAs

lncRNAs act as a ceRNA to sequester miRNAs, thereby derepressing key oncogenes and promoting cancer hallmarks such as enhanced proliferation, invasion, and metastasis (129). A growing body of evidence highlights the prevalence of the ceRNA network in OSCC pathogenesis. For instance, LINC01296 functions as a ceRNA for miR-485-5p, upregulating PAK4 and activating the MAPK/ERK pathway to promote tumor progression (130). Similarly, LINC01929 accelerates OSCC progression by functioning as a ceRNA that sponges miR-137-3p to upregulate FOXC1 expression (131), while small nucleolar RNA host gene 17 (SNHG17) exerts its oncogenic role by sponging miR-375 to upregulate PAX6 expression (132). Further demonstrating the functional breadth of this mechanism, FOXD2 antisense RNA 1 (FOXD2-AS1) promotes cancer cell proliferation, migration, and invasion by sponging miR-378g to upregulate cellular retinoic acid binding protein 2 (CRABP2) (133). Although these findings establish its role in core malignant behaviors, the specific impact of the FOXD2-AS1/miR-378g/CRABP2 axis on chemosensitivity and EMT remains an open and clinically significant question for future research.

Beyond ceRNA networks, several oncogenic lncRNAs exert their effects by directly interacting with key proteins to alter their stability or function (134). MAGEA4 antisense RNA 1 (MAGEA4-AS1) binds to the p53 protein and enhances the transcription of MAPK activated protein kinase 2 (MK2), thereby promoting proliferation and metastasis (135). DUXAP9, whose expression is driven by Yin Yang 1 factor (YY1), binds to enhancer of EZH2 and blocks its cyclin-dependent kinase 1 (CDK1)-mediated degradation. This interaction stabilizes the EZH2 protein and fuels tumor growth (136). Additionally, LINC00319 promotes malignancy by directly binding to and activating STAT3 signaling (137). These findings underscore that direct lncRNA-protein interactions constitute a crucial mechanism through which oncogenic lncRNAs regulate key signaling molecules to drive OSCC progression.

Remodeling the metabolic landscape of the TME is another critical function of oncogenic lncRNAs (138). HOXA11 antisense RNA (HOXA11-AS) enhances metastatic potential by differentially regulating NAD(P)H quinone dehydrogenase 1 (NQO1) and NQO2, thereby rewiring cellular energy production from glycolysis towards glutaminolysis to support survival (139). Likewise, cytoskeleton regulator RNA (CYTOR) drives aberrant glycolysis and mitochondrial respiration by interacting with heterogeneous nuclear ribonucleoprotein C (HNRNPC) to stabilize ZEB1 mRNA (140). These examples illustrate how oncogenic lncRNAs reprogram cellular metabolism within the TME to fuel OSCC progression.

Notably, oncogenic lncRNAs can actively remodel TME by targeting and reprogramming key tumor associated cells, such as cancer-associated fibroblasts (CAFs) and TAMs (141). CAFs-derived exosomal lncRNA FTX can be transferred to OSCC cells, where it binds to and upregulates flap structure-specific endonuclease 1 (FEN1) by recruiting TET2 to demethylate the FEN1 promoter. The FTX/FEN1 complex then transcriptionally represses acyl-CoA synthetase long chain family member 4 (ACSL4), thereby promoting cell motility (142). Another lncRNA, LOC100506114, which is expressed in CAFs, promotes stromal fibroblast activation and tumor progression by upregulating growth differentiation factor 10 (GDF10) secretion (143). Furthermore, DCST1 antisense RNA 1 (DCST1-AS1) promotes immunosuppressive TME by driving M2 macrophage polarization through activation of the NF-κB signaling pathway (144). These findings highlight the crucial role of oncogenic lncRNAs in shaping a pro-tumorigenic TME by directly modulating the functions of stromal and immune cells.

Beyond modulating tumor associated cells, certain lncRNAs directly target and impair the function of cytotoxic T lymphocytes (CTLs), a key anti-tumor immune component within the TME, thereby facilitating immune evasion. The lncRNA LINC01355 also contributes to an immunosuppressive TME by inhibiting CD8^+^ T cell activity. Silencing LINC01355 in OSCC was shown to repress tumor growth by enhancing CD8^+^ T cell immune responses. Specifically, downregulation of LINC01355 restrained CD8^+^ T cell apoptosis, increased the percentage of CD8^+^ T cells, and enhanced their cytolytic activity when co-cultured with OSCC cells. This effect is mediated through the Notch signaling pathway, as loss of LINC01355 inactivates Notch signaling, which is known to repress CD8^+^ T cell activity in cancer (145). Thus, the direct suppression of CTLs by specific lncRNAs represents a key mechanism for establishing immunosuppressive TME and facilitating immune evasion in OSCC.

##### 3.3.1.2 Tumor suppressive lncRNAs

A distinct subset of lncRNAs functions as tumor suppressors in OSCC, and their frequent downregulation contributes to tumor progression. These lncRNAs employ diverse mechanisms to exert their anticancer effects, primarily through acting as molecular sponges for miRNAs, interacting with proteins to modulate key signaling pathways, and influencing cellular differentiation states.

Several tumor suppressive lncRNAs operate through the ceRNA mechanism, where they sequester oncogenic miRNAs and prevent them from repressing their tumor suppressive target genes. For instance, LINC00472 is downregulated in OSCC and acts as a sponge for miR-4311, thereby positively regulating the expression of G protein subunit gamma 7 (GNG7) to inhibit tumor progression (146). Similarly, HLA complex group 22 (HCG22) exerts its inhibitory effects on proliferation, invasion, and migration by downregulating both miR-425-5p and miR-650 (147). The lncRNA prostate androgen-regulated transcript 1 (PART1), which can be packaged into exosomes, suppresses malignant progression by functioning as a sponge for miR-17-5p, which leads to the upregulation of suppressor of cytokine signaling 6 (SOCS6) expression (148). Besides, PCBP1 antisense RNA 1 (PCBP1-AS1) suppresses OSCC cell growth by acting as a molecular sponge for miR-34c-5p, which consequently increases the expression of the miR-34c-5p target gene ZFP36 ring finger protein (ZFP36) (149). Collectively, these examples underscore the functional importance of tumor-suppressive lncRNAs operating through the ceRNA network to restrain OSCC progression.

Another crucial mechanism involves lncRNAs that directly interact with proteins or transcription factors to disrupt oncogenic signaling (134). Maternally expressed 3 (MEG3) is frequently downregulated in OSCC and inhibits cancer progression by interacting with the transcription factor GATA binding protein 3 (GATA3) (150). Terminal differentiation-inducing non-protein coding RNA (TINCR) induces cell differentiation and suppresses tumorigenesis by modulating the JAK2/STAT3 signaling pathway, with its downregulation predicting poor prognosis (151). LINC00173 exerts its tumor suppressive function by binding to GATA6 and blocking its ability to transcriptionally activate collagen type V alpha 1 chain (COL5A1), a promoter of malignancy (152). Thus, the direct binding of tumor-suppressive lncRNAs to key regulatory proteins or transcription factors constitutes an effective mechanism for disrupting oncogenic signaling in OSCC.

The regulation of tumor angiogenesis, a critical process in TME remodeling, is also modulated by tumor suppressive lncRNAs through distinct molecular axes (153). Research has demonstrated that the enforced expression of MEG3 in OSCC cells reduces the levels of exosomal miR-421. This exosomal miR-421, when transferred to human umbilical vein endothelial cells (HUVECs), targets and downregulates heparan sulfate 2-O-sulfotransferase 1 (HS2ST1). The suppression of HS2ST1 activates the vascular endothelial growth factor (VEGF)/vascular endothelial growth factor receptor-2 (VEGFR2) signaling pathway, specifically promoting ERK and AKT phosphorylation, which drives endothelial cell migration, invasion, and tube formation. Therefore, MEG3 acts as a tumor suppressor by attenuating this exosomal miR-421/HS2ST1 mediated pro angiogenic signaling cascade (154). In a separate mechanism, the lncRNA NR2F2 antisense RNA 1 (NR2F2-AS1) exerts its anti-angiogenic effect by functioning as a ceRNA. NR2F2-AS1 directly binds to and sequesters miR-32-5p, which leads to the upregulation of semaphorin 3A (SEMA3A), a known inhibitor of angiogenesis. The overexpression of NR2F2-AS1, through the miR-32-5p/SEMA3A axis, consequently inhibits the tube formation ability of HUVECs, thereby suppressing angiogenesis (155). These findings underscore that tumor suppressive lncRNAs can impede OSCC progression by directly interfering with key signaling pathways that govern vascular expansion within the TME.

The tumor suppressive role of lncRNAs extends beyond the cancer cells themselves to encompass intercellular communication within the TME. A notable example is exosomal lncRNA LBX1 antisense RNA 1 (LBX1-AS1), which is derived from recombination signal binding protein for immunoglobulin kappa J region (RBPJ) overexpressed macrophages. Upon transfer to OSCC cells, this exosomal lncRNA inhibits tumor progression by sponging miR-182-5p and upregulating the expression of forkhead box O3 (FOXO3) (156). Thus, lncRNAs can exert tumor-suppressive effects across cellular boundaries within the TME through mechanisms such as exosomal transfer.

In summary, lncRNAs have emerged as central regulators in OSCC pathogenesis, functioning as potent oncogenes or tumor suppressors. They act as ceRNAs, modulating proteins and transcription factors, and reprogramming TME metabolism. Critically, lncRNAs actively reshape the TME by targeting stromal and immune components, such as CAFs, TAMs, and CTLs, thereby influencing processes like angiogenesis and immune evasion. Their frequent dysregulation within these intricate networks underscores their potential as diagnostic and prognostic biomarkers, as well as therapeutic targets for OSCC.

#### 3.3.2 miRNAs in OSCC

**Figure 7:**
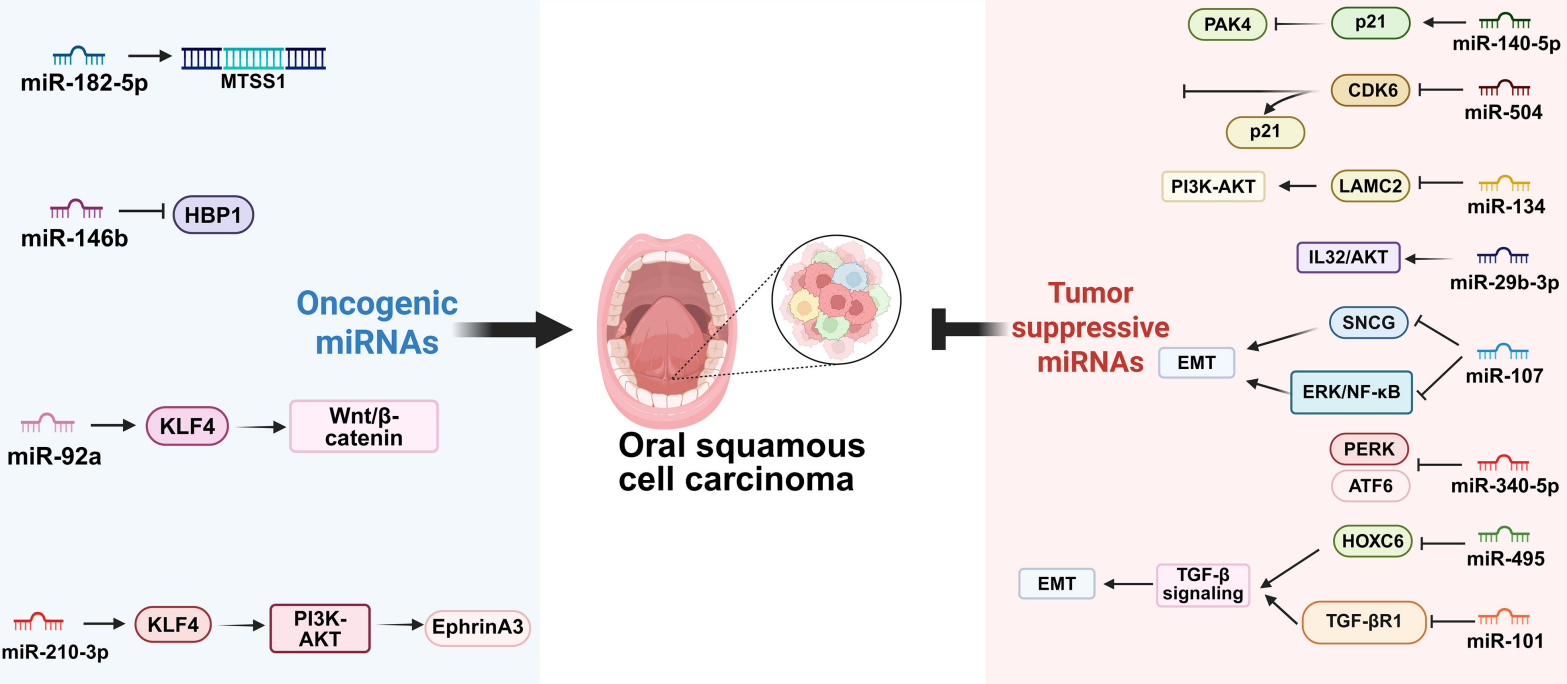
Regulatory network of microRNAs (miRNAs) in OSCC. miRNAs function as potent oncogenes or tumor suppressors to coordinately regulate gene expression. They exert oncogenic or tumor suppressive effects on tumor cell proliferation, apoptosis, invasion and stemness, and dynamically remodel the tumor microenvironment (TME).

miRNAs function as pivotal regulators in the pathogenesis of OSCC by modulating key oncogenic and tumor-suppressive pathways (**Figure 7**) (157). Their expression is frequently dysregulated in OSCC, influencing critical cellular processes such as proliferation, apoptosis, invasion, and angiogenesis (158).

##### 3.3.2.1 Oncogenic miRNAs

In OSCC, the aberrant overexpression of specific miRNAs promotes aggressive malignant phenotypes, such as migration and invasion, through the direct targeting of critical tumor suppressor genes. miR-182-5p is upregulated in OSCC and promotes migration and invasion by directly targeting the MTSS I-BAR domain containing 1 (MTSS1) gene (159). Similarly, miR-146b also functions as an oncogene in OSCC by targeting HMG-box transcription factor 1 (HBP1), and its inhibition decreases OSCC cell proliferation, migration, and invasion (160).

Oncogenic miRNAs often exert their effects by activating critical signaling pathways that drive cancer progression. miR-92a, which is highly expressed in OSCC cell lines, promotes proliferation and inhibits apoptosis by targeting KLF transcription factor 4 (KLF4) and activating the Wnt/β-catenin signaling pathway (161). Another example is miR-210-3p, which targets EphrinA3 and regulates OSCC progression through the PI3K/AKT axis, influencing EMT and other malignant behaviors (162).

##### 3.3.2.2 Tumor-suppressive miRNAs

Several miRNAs exert their tumor-suppressive functions by directly targeting genes that drive cell cycle progression and survival. For instance, miR-140-5p is notably downregulated in OSCC. It inhibits tumorigenesis by targeting PAK4, thereby suppressing cell proliferation and inducing cell cycle arrest and apoptosis (163). However, the regulatory effects of the miR-140-5p/PAK4 axis on OSCC metastasis remain to be fully elucidated, warranting further investigation in both *in vitro* and *in vivo* models. Similarly, miR-504 acts as a tumor suppressor by targeting CDK6. Its overexpression leads to the inhibition of OSCC cell proliferation, migration, and invasion, accompanied by an increase in the expression of the cell cycle inhibitor p21 (164). Nevertheless, the broader and more intricate regulatory networks connecting miR-504 to other cycle-related and autophagy-related genes remain to be fully elucidated.

The metastatic potential of OSCC is critically restrained by a subset of miRNAs. miR-134 inhibits the migration and invasion of OSCC tumor stem cells by targeting laminin subunit gamma 2 (LAMC2), which leads to the downregulation of the PI3K/AKT signaling pathway (165). miR-29b-3p suppresses OSCC cell migration and invasion via the IL32/AKT signaling pathway and is found to be downregulated in highly invasive cells (166). Furthermore, miR-107 modulates EMT progression by targeting synuclein gamma (SNCG) and inhibiting the ERK/NF-κB signaling pathways, thereby attenuating OSCC cell migration and invasion (167). miR-340-5p affects OSCC cell proliferation and invasion by targeting endoplasmic reticulum stress proteins PERK and ATF6 (168). Collectively, these miRNAs constitute a key regulatory network that restrains OSCC metastasis by targeting multiple signaling pathways.

Angiogenesis is a vital process for tumor growth and is negatively regulated by specific miRNAs. miR-378a-5p inhibits angiogenesis in OSCC by targeting kallikrein-related peptidase 4 (KLK4). Its inhibition reduces tube formation of HUVECs and newly formed microvessels, an effect that can be reversed by KLK4 overexpression (169).

Transforming growth factor beta (TGF-β) signaling pathway is a pivotal regulator within the TME, influencing cancer cell stemness, immune evasion, and stromal activation (170). Tumor-suppressive miRNAs can impede OSCC progression by directly targeting key components of this pathway. Specifically, miR-495 is significantly downregulated in OSCC. It exerts its inhibitory function by directly targeting and suppressing HOXC6. This action leads to the inhibition of the TGF-β signaling pathway, resulting in suppressed EMT, reduced proliferation, migration, and invasion of CSCs, and promoted apoptosis both *in vitro* and *in vivo* (171). Given the constraints imposed by the relatively small patient cohort, the precise mechanistic role of miR-495 remains incompletely understood, necessitating future large-scale investigations to fully delineate its underlying regulatory pathways. In a parallel mechanism, miR-101, which is also markedly downregulated in OSCC, directly targets the transforming growth factor-β receptor 1 (TGF-βR1). By downregulating TGF-βR1, miR-101 inhibits OSCC cell proliferation, migration, invasion, and pro-angiogenic capacity while inducing cell apoptosis (172). These findings highlight that miRNAs such as miR-495 and miR-101 function as critical negative regulators of the oncogenic TGF-β axis, thereby disrupting key TME-facilitated processes that drive OSCC malignancy.

In summary, miRNAs are pivotal regulators in OSCC, functioning as potent oncogenes or tumor suppressors. They exert profound influence over tumorigenesis by fine-tuning the expression of key genes involved in critical cellular processes such as proliferation, apoptosis, metastasis, angiogenesis and TGF-β signaling pathway. Their dysregulation often occurs through intricate interactions within larger regulatory networks, positioning them as promising candidates for both diagnostic biomarkers and novel therapeutic targets in OSCC.

#### 3.3.3 circRNAs in OSCC

**Figure 8:**
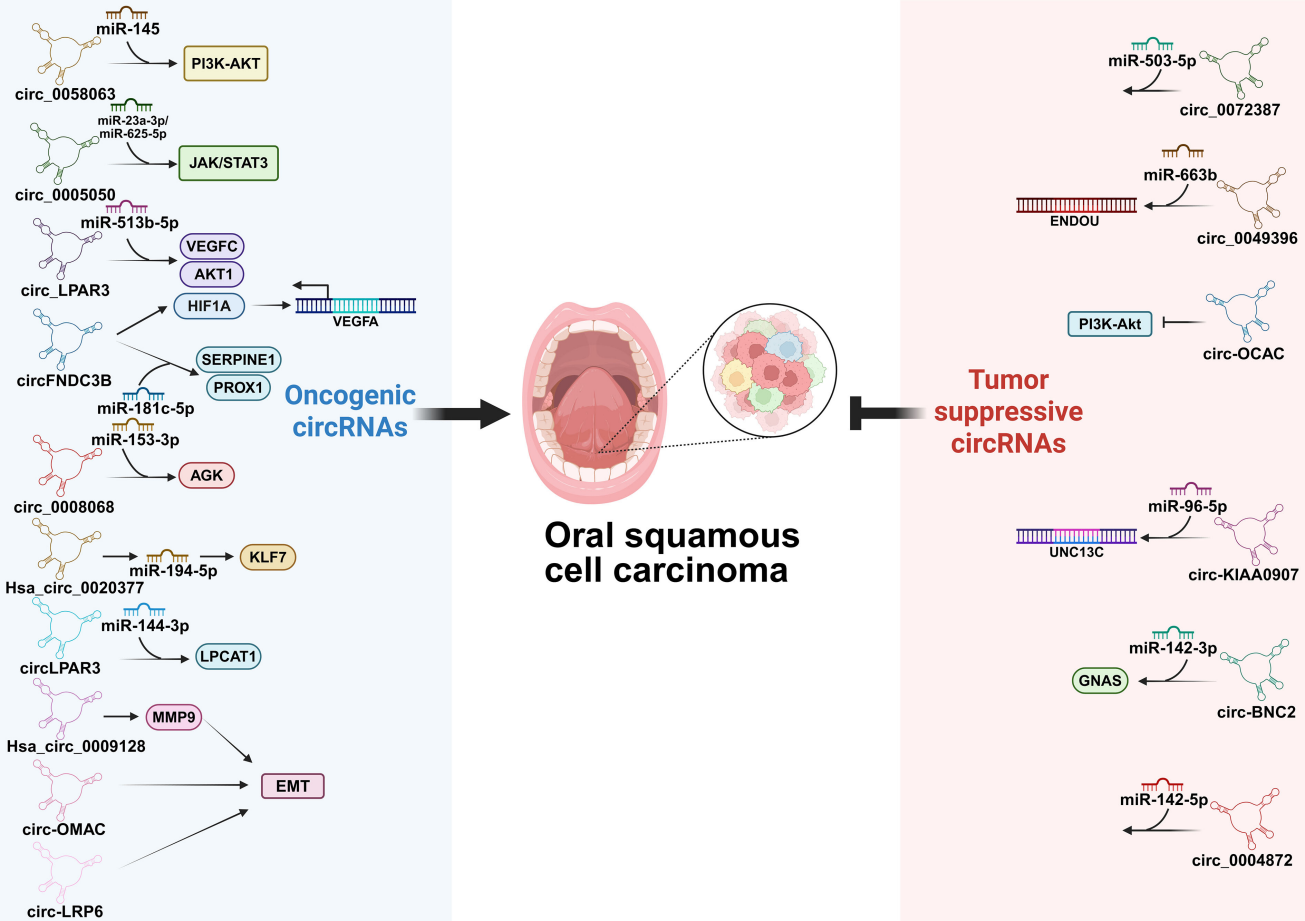
Regulatory network of circular RNAs (circRNAs) in OSCC. circRNAs interact through multiple mechanisms, such as the competitive endogenous RNA network where circRNAs sponge miRNAs to coordinately regulate gene expression. They exert oncogenic or tumor suppressive effects on tumor cell proliferation, apoptosis, invasion and stemness, and dynamically remodel the tumor microenvironment (TME).

circRNAs are a novel class of endogenous ncRNAs that have emerged as pivotal regulators in OSCC (173). They frequently function as molecular sponges for miRNAs, thereby derepressing miRNA targets and influencing various aspects of tumor biology (174). Functioning as key miRNA sponges, specific circRNAs are central regulators in OSCC. Their dysregulation promotes hallmark malignant phenotypes, including proliferation, metabolic reprogramming, metastasis, and therapy resistance, by orchestrating oncogenic signaling and remodeling the TME, notably through angiogenesis (**Figure 8**) (175).

##### 3.3.3.1 Oncogenic circRNAs

A substantial number of circRNAs are upregulated in OSCC and contribute to tumor aggressiveness. These molecules often exert their effects by activating core oncogenic signaling cascades. For instance, circ_0058063 is highly expressed in OSCC and promotes tumor development by sponging miR-145, which leads to the activation of the PI3K/AKT pathway and enhances cell viability, adhesion, migration, and EMT (176). Given that miR-145-5p is known to target multiple mRNAs beyond this axis, future research direction is to systematically identify the full repertoire of downstream effector genes regulated by the circ_0058063/miR-145-5p network. Similarly, circ_0005050 drives OSCC proliferation and inhibits apoptosis by competitively binding to both miR-23a-3p and miR-625-5p, resulting in the upregulation of STAT3 and consequent activation of the JAK/STAT3 signaling pathway (177). Thus, upregulated circRNAs in OSCC primarily drive tumor progression by sponging miRNAs and subsequently activating key oncogenic signaling pathways.

Beyond intracellular signaling, oncogenic circRNAs actively remodel the TME to support tumor growth and dissemination. The induction of angiogenesis is a critical step in this process. circ_LPAR3, which is elevated in OSCC, facilitates tumor growth and angiogenesis by acting as a sponge for miR-513b-5p, thereby increasing the expression of VEGF-C and AKT serine/threonine kinase 1 (AKT1) (178). circFNDC3B is upregulated in OSCC and acts as a central regulator of both angiogenesis and lymphangiogenesis. Mechanistically, circFNDC3B promotes the transcription of the key pro-angiogenic factor VEGFA by enhancing HIF1A stability. Simultaneously, it functions as a ceRNA by sequestering miR-181c-5p, leading to the upregulation of serpin family E member 1 (SERPINE1) and prospero homeobox 1 (PROX1), which in turn drives lymphangiogenesis. This dual function of circFNDC3B in stimulating blood and lymphatic vessel formation creates a pro-metastatic microenvironment that facilitates tumor cell dissemination and lymph node metastasis, highlighting the profound influence of ncRNAs on the TME’s vascular compartment (179). Collectively, oncogenic circRNAs critically shape a pro-metastatic TME by driving the formation of new blood and lymphatic vessels.

Metabolic reprogramming towards glycolysis, known as the Warburg effect, is another hallmark of cancer that is regulated by circRNAs. circ_0008068 accelerates OSCC development by sequestering miR-153-3p, which elevates the expression of acylgycerol kinase (AGK) and subsequently enhances glycolysis in addition to promoting proliferation and invasion (180). Likewise, hsa_circ_0020377 facilitates tumor cell malignant behaviors and glycolysis by sponging miR-194-5p, which leads to the consequent upregulation of its target gene KLF7 (181). Furthermore, circLPAR3 promotes OSCC progression by functioning as a sponge for miR-144-3p, which results in the upregulation of lysophosphatidylcholine acyltransferase 1 (LPCAT1). Silencing circLPAR3 was shown to repress OSCC cell glycolysis, highlighting its role in modulating this TME-associated metabolic pathway (182). These findings collectively illustrate that circRNAs act as pivotal ceRNAs to enhance glycolytic metabolism, thereby fueling a metabolic TME conducive to OSCC progression.

The promotion of metastasis is a central function of many oncogenic circRNAs, primarily through the induction of EMT. Hsa_circ_0009128 is upregulated in OSCC and correlates with advanced TNM stage and lymph node metastasis. Its knockdown inhibits cell migration and suppresses EMT by downregulating the expression of MMP-9 (183). Another circRNA, circ-OMAC, is aberrantly elevated in metastatic lymph nodes and is associated with poor prognosis. It was demonstrated to promote OSCC metastasis via the initiation of EMT signaling pathways (184). Nevertheless, the precise molecular mechanisms underpinning circ-OMAC’s oncogenic functions, such as its interacting miRNAs or proteins, remain to be fully elucidated. Furthermore, circ-LRP6 was found to mediate both EMT and autophagy in OSCC, with increased autophagy being able to rescue the EMT process inhibited by circ-LRP6 knockdown, highlighting a complex interplay in driving metastasis (185). These studies demonstrate that promoting EMT is a major mechanism through which oncogenic circRNAs drive metastasis in OSCC.

##### 3.3.3.2 Tumor-suppressive circRNAs

In contrast to their oncogenic counterparts, a distinct group of circRNAs functions as tumor suppressors in OSCC. These molecules are frequently downregulated, and their restoration presents a promising therapeutic avenue (186). Their protective effects are mediated through the inhibition of various cancer-promoting processes.

A subset of tumor-suppressive circRNAs in OSCC exerts inhibitory effects primarily by regulating key cellular proliferation and apoptotic pathways. For instance, circ_0049396 is downregulated in OSCC and functions by sequestering miR-663b, which leads to the upregulation of its target endonuclease (ENDOU). This circ_0049396/miR-663b/ENDOU axis potently suppresses OSCC cell proliferation and migration while promoting apoptosis, as further validated *in vivo* (187). Similarly, circ-KIAA0907 impedes tumor growth and enhances radiosensitivity by competitively binding to miR-96-5p, thereby augmenting the expression of unc-13 homolog C (UNC13C) (188). In summary, these tumor-suppressive circRNAs form a regulatory network that constrains OSCC growth by critically modulating cell proliferation and apoptotic fate.

Another key mechanism involves the direct inhibition of core oncogenic signaling pathways. circ-OCAC is significantly downregulated in OSCC samples. Functional studies revealed that it inhibits OSCC growth and metastasis by blocking the PI3K/AKT signaling pathway, a central driver of tumorigenesis. The translational potential of this finding was highlighted by the development of a pH-responsive nanoparticle that efficiently delivered circ-OCAC, resulting in significant tumor suppression *in vivo* (189).

Another group of circRNAs impedes OSCC progression by suppressing invasion, metastasis, and EMT. circ-BNC2, which is downregulated in OSCC tissues, acts as a molecular sponge for miR-142-3p to upregulate the tumor suppressor GNAS. This mechanism inhibits cell proliferation, migration, and invasion, while inducing apoptosis and oxidative stress (190). In a related mechanism, the downregulation of circ_0072387 is associated with poor OSCC progression. Its overexpression suppresses proliferation, metastasis, and EMT by sponging miR-503-5p (191). By functioning as miRNA sponges, these circRNAs establish a regulatory axis that effectively constrains OSCC invasion and metastasis.

Furthermore, the role of circRNAs in modulating cancer metabolism, particularly glycolysis, represents a distinct functional layer in OSCC suppression. circ_0004872 is significantly downregulated in OSCC and inhibits cell proliferation, invasion, and the glycolytic pathway by acting as a sponge for miR-424-5p (192). This metabolic regulatory function is shared by circ_0072387, which also suppresses the Warburg effect (glycolytic metabolism) in OSCC cells (191). Collectively, these findings highlight a novel metabolic regulatory layer by which circRNAs suppress OSCC progression.

In summary, circRNAs constitute a critical layer of regulatory network in OSCC. They function primarily through the ceRNA mechanism to modulate the activity of key oncogenic or tumor-suppressive pathways. Their involvement in vital processes such as cell proliferation, apoptosis, metabolism, angiogenesis, and metastasis underscore their potential as both valuable diagnostic and prognostic biomarkers and promising therapeutic targets for OSCC.

The intricate roles of ncRNAs in OSCC extend beyond the aforementioned epigenetic mechanisms, forming a complex regulatory network that governs tumorigenesis and treatment response (**Figures 6**-**8**). **Table 2** summarizes the diverse mechanisms and functions of key ncRNAs in OSCC.

**Table 2:** The role of ncRNAs in OSCC onset and progression.

ncRNA	Gene	Regulation	Target	Mechanism	Function	Ref.
lncRNA	LINC01296	Upregulated	miR-485-5p/PAK4/ MAPK/ERK	LINC01296 acting as a ceRNA sequesters miR-485-5p, leading to PAK4 upregulation and subsequent activation of the MAPK/ERK signaling pathway.	Promotes the cell cycle, proliferation, migration and invasion, and inhibit apoptosis of OSCC cells.	(130)
	LINC01929	Upregulated	miR-137-3p/FOXC1	LINC01929 acting as a ceRNA by competitively binding to and sequestering miR-137-3p, which leads to the upregulation of the oncogenic transcription factor FOXC1.	Accelerates OSCC cell proliferation, migration and invasion, and suppression of apoptosis.	(131)
	SNHG17	Upregulated	miR-375/PAX6	SNHG17 acting as a molecular sponge for tumor-suppressive miR-375, leading to the upregulation of PAX6.	Accelerates proliferation and metastasis of OSCC cells, while reducing apoptosis.	(132)
	FOXD2-AS1	Upregulated	miR-378g/CRABP2	FOXD2-AS1 acts as a molecular sponge for miR-378g, thereby upregulating the expression of CRABP2.	Enhances OSCC malignant cell behaviors.	(133)
	MAGEA4-AS1	Upregulated	p53/MK2	MAGEA4-AS1 forms a complex with p53 that binds to the MK2 promoter, enhancing MK2 transcription and activating downstream oncogenic signaling pathways.	Promotes the proliferation and metastasis of OSCC cells.	(135)
	DUXAP9	Upregulated	EZH2	DUXAP9 interacts with and stabilizes EZH2 to suppress EZH2 phosphorylation and subsequent nuclear-to-cytoplasmic translocation, thereby preventing EZH2 degradation.	Promotes OSCC cell proliferation, migration, invasion, and xenograft tumor growth and metastasis.	(136)
	LINC00319	Upregulated	STAT3	LINC00319 directly binds to STAT3 and facilitates its phosphorylation at Tyr705, leading to constitutive activation of the STAT3 signaling pathway.	Enhances the proliferation, migration, invasion, and EMT of OSCC cells.	(137)
	HOXA11-AS	Upregulated	miR-494/NQO1/EZH2/NQO2	HOXA11-AS sponges miR-494 to upregulate NQO1 and recruits EZH2 to the NQO2 promoter.	Promotes proliferation, invasion, survival, and drug resistance.	(139)
	CYTOR	Upregulated	HNRNPC/ZEB1	CYTOR interacts with HNRNPC in the nucleus and stabilizes ZEB1 mRNA by inhibiting its nondegradative ubiquitination.	Promotes migration, invasion and EMT in oral cancer cells.	(140)
	FTX	Upregulated	FEN1/TET2/ACSL4	FTX forms an RNA-protein complex with FEN1 to recruit TET2 to induce promoter demethylation of FEN1. The FTX/FEN1 axis subsequently transcriptionally represses ACSL4 to inhibit ferroptosis.	Promotes OSCC cells motility.	(142)
	LOC100506114	Upregulated	GDF10	LOC100506114 drives the activation of cancer-associated fibroblast, which in turn enhances tumor cell proliferation and migration through the secretion of GDF10.	Promotes tumor cell proliferation and migration.	(143)
	DCST1-AS1	Upregulated	NF-κB	DCST1-AS1 activates the NF-κB signaling pathway.	Promotes OSCC progression and M2 macrophage polarization.	(144)
	LINC01355	Upregulated	Notch signaling	LINC01355 activates the Notch signaling pathway (Notch-1/JAG-1/HES-1 axis), which represses CD8+ T cell activity in TME.	Downregulation of LINC01355 significantly restrained CD8^+^ T cell apoptosis, induced CD8^+^ T cell percentage, and enhanced the cytolysis activity when cocultured with OSCC cells.	(145)
	MEG3	Downregulated	H3K27me3/GATA3	MEG3 expression is silenced by H3K27me3 modification, while its tumor-suppressive function depends on binding to the transcription factor GATA3 to activate downstream genes that inhibit the Wnt signaling pathway.	Its over-expression can inhibit proliferation, migration, and invasion and promote apoptosis of OSCC cells.	(150)
			miR-421/HS2ST1	MEG3 reduces exosomal miR-421 transfer to endothelial cells, which relieves miR-421-mediated suppression of HS2ST1 and thereby activates the pro-angiogenic VEGF/VEGFR2/ERK/AKT pathway.	By inhibiting exosomal miR-421/HS2ST1-mediated angiogenesis, MEG3 suppresses endothelial cell migration, invasion, and tube formation, exerting a tumor-suppressive effect in OSCC.	(154)
	TINCR	Downregulated	JAK2/STAT3	TINCR inhibits the JAK2/STAT3 signaling pathway.	TINCR functions as a tumor suppressor by inducing cell differentiation.	(151)
	NR2F2-AS1	Downregulated	miR-32-5p/SEMA3A	NR2F2-AS1 acts as a ceRNA by sponging miR-32-5p, leading to the upregulation of the angiogenesis inhibitor SEMA3A.	The inhibitory effects of NR2F2-AS1 overexpression on EMT, migration, invasion of OSCC cells, and angiogenesis of HUVECs as well as tumor growth and metastasis in mice were mediated via the miR-32-5p/SEMA3A axis.	(155)
	LINC00472	Downregulated	miR-4311/ GNG7	LINC00472 acts as a molecular sponge for hsa-miR-4311, thereby upregulating the expression of GNG7.	Over-expression could suppress xenograft tumor growth *in vivo*.	(146)
	HCG22	Downregulated	miR-425-5p/miR-650	HCG22 directly binds to and sequesters oncogenic miR-425-5p.	Upregulation of the expression can inhibit the proliferation, migration, invasion, and EMT of OSCC cells.	(147)
	PART1	Downregulated	miR-17-5p/SOCS6	PART1 upregulates SOCS6 through sponging miR-17-5p.	Inhibits OSCC cell viabilities, migration, and invasiveness but facilitates OSCC cell apoptosis.	(148)
		Downregulated	miR-34c-5p/ZFP36	PCBP1-AS1 upregulates ZFP36 through interaction with miR-34c-5p.	Hampers cell proliferation and promotes cell apoptosis in OSCC.	(149)
	PCBP1-AS1	Downregulated	GATA6/COL5A	Blocks GATA6-mediated transcription of COL5A1.	Promotes proliferation, migration and invasiveness, apoptosis resistance, and pro-angiogenic ability of OSCC cells.	(152)
	LBX1-AS1	Upregulated	miR-182-5p/FOXO3	LBX1-AS1 acts as a ceRNA for miR-182-5p, leading to upregulation of FOXO3.	Inhibits tumor progression.	(156)
miRNA	miR-182-5p	Upregulated	MTSS1	miR-182-5p directly targets and suppresses MTSS1.	Promotes the migration and invasion of OSCC.	(159)
	miR-146b	Upregulated	HBP1	miR-146b directly targets and suppresses HBP.	Promotes proliferation, migration, and invasion of OSCC cells.	(160)
	miR-92a	Upregulated	KLF4/Wnt/β-catenin	miR-92a directly targets and suppresses KLF4, leading to activation of the Wnt/β-catenin signaling pathway.	Inhibits proliferation and promotes apoptosis of OSCC cells.	(161)
	miR-210-3p	Upregulated	EFNA3/PI3K/AKT	miR-210-3p targets EFNA3, leading to activation of the PI3K/AKT signaling pathway.	Promotes EMT.	(162)
	miR-140-5p	Downregulated	PAK4	miR-140-5p induces cell-cycle arrest by directly targeting and downregulating PAK4.	Over-expression suppresses cell proliferation, promotes cell apoptosis, and induces cell-cycle arrest in OSCC.	(163)
	miR-504	Downregulated	CDK6/E2F1/Cyclin D1/p21	miR-504 directly targets CDK6, leading to cell cycle arrest via downregulation of E2F1 and Cyclin D1, and upregulation of p21.	Inhibits cell proliferation, migration and invasion.	(164)
	miR-134	Downregulated	LAMC2/PI3K/AKT	miR-134 directly targets and downregulates LAMC2, leading to inhibition of the PI3K-AKT signaling pathway.	Inhibits tumor stem cell migration and invasion.	(165)
	miR-29b-3p	Downregulated	IL32/AKT	miR-29b-3p targets the IL32/AKT signaling pathway, serving as a guardian against tumor progression.	Suppresses cell migration and invasion.	(166)
	miR-378a-5p	Downregulated	KLK4	miR-378a-5p inhibits angiogenesis in OSCC by targeting KLK4.	Inhibits angiogenesis in OSCC	(169)
	miR-495	Downregulated	HOXC6/TGF-β	miR-495 directly targets and downregulates HOXC6, leading to the inhibition of the TGF-β signaling pathway.	Inhibit EMT, proliferation, migration, and invasion while promoting apoptosis of CSCs in OSCC.	(171)
	miR-101	Downregulated	TGF-βR1	miR-101 directly targets and downregulates TGF-βR1, a key receptor of the TGF-β signaling pathway.	miR-101 significantly abolishes the proliferation, motility, and proangiogenesis of OSCC cells and induces their apoptosis.	(172)
	miR-107	Downregulated	SNCG/ERK1/2/NF-κB	miR-107 directly targets SNCG, thereby inhibiting the ERK1/2 and NF-κB signaling pathways.	Attenuates migration and EMT.	(167)
	miR-340-5p	Downregulated	ATF6/ PERK	miR-340-5p directly targets endoplasmic reticulum stress regulators ATF6 and PERK.	Suppresses OSCC cell proliferation and invasion.	(168)
circRNA	circ_0058063	Upregulated	miR-145-5p/PI3K/AKT	circ_0058063 sponges miR-145-5p, leading to activation of the PI3K/AKT signaling pathway.	Decreases OSCC cell viability, adhesion, migration, and EMT.	(176)
	circ_0005050	Upregulated	miR-23a-3p/miR-625-5p/STAT3/JAK	circ_0005050 acts as a molecular sponge for both miR-23a-3p and miR-625-5p, leading to the upregulation of STAT3 and subsequent activation of the JAK/STAT3 signaling pathway.	Facilitates OSCC cell proliferation and inhibiting cell apoptosis.	(177)
	circ_LPAR3	Upregulated	miR-513b-5p/VEGFC/AKT1	circ_LPAR3 acts as a miR-513b-5p sponge, thereby upregulating VEGFC and enhancing AKT1 phosphorylation.	Decreases cell survival and mobility and in mice model.	(178)
	circFNDC3B	Upregulated	HIF1A/VEGFA/miR-181c-5p	circFNDC3B activates HIF1A/VEGFA signaling and sponges miR-181c-5p to upregulate SERPINE1 and PROX1.	Accelerates vasculature formation and metastasis.	(179)
	circ_0008068	Upregulated	miR-153-3p/AGK	circ_0008068 acts as a molecular sponge for miR-153-3p, leading to upregulation of AGK expression.	Stimulates cell apoptosis in OSCC.	(180)
	Hsa_circ_0020377	Upregulated	miR-194-5p/KLF7	Hsa_circ_0020377 sponges miR-194-5p to upregulate KLF7 expression.	Facilitates tumor cell malignant behaviors and glycolysis.	(181)
	circLPAR3	Upregulated	miR-144-3p/LPCAT1	circLPAR3 functions as a sponge for miR-144-3p, leading to the upregulation of LPCAT1.	circLPAR3 silencing represses cell proliferation, migration, invasion, angiopoiesis, glycolysis, and induces cell apoptosis in OSCC cells *in vitro*.	(182)
	Hsa_circ_0009128	Upregulated	MMP9	Hsa_circ_0009128 upregulates MMP9 to induce EMT.	Stimulates proliferation and migration in OSCC cells.	(183)
	circ-OMAC	Upregulated	E-cadherin/ N-cadherin/vimentin	circ-OMAC decreases the E-cadherin protein level, while leading to the upregulation of N-cadherin and vimentin.	Drives metastasis in OSCC.	(184)
	circ-LRP6	Upregulated	LC3B/vimentin/Zeb1	circ-LRP6 inhibits LC3B, vimentin and Zeb1.	Promotes EMT and autophagy of OSCC and increases autophagy.	(185)
	circ_0072387	Downregulated	miR-503-5p	circ_007238 acts as a molecular sponge for miR-503-5p.	Suppresses proliferation, metastasis, and glycolysis.	(191)
	circ_0049396	Downregulated	miR-663b/ENDOU	circ_0049396 sequesters miR-663b, which leads to the upregulation of the target gene ENDOU.	Suppresses OSCC cell malignancy.	(187)
	circ_KIAA0907	Downregulated	miR-96-5p/UNC13C	circ-KIAA0907 binds to miR-96-5p, thereby augmenting the expression of UNC13C.	Inhibits the progression of OSCC.	(188)
	circ-OCAC	Downregulated	PI3K/AKT	Circ-OCAC inhibits the PI3K/AKT signaling pathway.	Inhibits OSCC growth and metastasis.	(189)
	circ-BNC2	Downregulated	miR-142-3/GNAS	Circ-BNC2 sponges miR-142-3p, thereby upregulating GNAS expression.	Represses the proliferation, migration and invasion of OSCC cells but induces cell apoptosis and oxidative stress.	(190)
	circ_0004872	Downregulated	miR-424-5p	Circ_0004872 sponges miR-424-5p to inhibit cell proliferation.	Inhibits proliferation, invasion, and glycolysis.	(192)

PAK4, P21-activated kinase 4; MAPK, Mitogen-activated protein kinase; ERK, Extracellular signal-regulated kinase; FOXC1, Forkhead box C1; CRABP2, Cellular retinoic acid binding protein 2; PAX6, Paired box 6; MK2, MAPK-activated protein kinase 2 ; EZH2, Enhancer of zeste homolog 2; STAT3, Signal transducer and activator of transcription 3; NQO1/2, Quinone dehydrogenase 1/2; HNRNPC, Heterogeneous nuclear ribonucleoprotein c; ZEB1, Zinc finger e-box binding homeobox 1; FEN1, Flap ftructure-specific endonuclease 1; TET2, Tet methylcytosine dioxygenase 2; ACSL4, Acyl-CoA synthetase long chain family member 4; GDF10, Growth differentiation factor 10; TIM-3, T cell immunoglobulin and mucin domain-containing protein3; H3K27me3, Histone h3 lysine 27 trimethylation; GATA3, GATA binding protein 3; HS2ST1, Heparan sulfate 2-O-sulfotransferase 1; JAK2, Janus kinase 2; SEMA3A, Semaphorin 3A; GNG7, G protein subunit gamma 7; SOCS6, Suppressor of cytokine signaling 6; ZFP36, ZFP36 ring finger protein; GATA6, GATA binding protein 6; COL5A, Collagen type V alpha 1 chain; KLF10, Kruppel-like factor 10; FOXO3, Forkhead box o3; CDK6, Cyclin dependent kinase 6; E2F1, E2F transcription factor 1; LAMC2, Laminin subunit gamma 2; PI3K, Phosphoinositide 3-Kinase; AKT, AKT serine/threonine kinase; TGF-β, Transforming growth factor beta; TGF-βR1, Transforming Growth Factor Beta Receptor 1; SNCG, Synuclein gamma; NF-κB, Nuclear factor kappa B; ATF6, Activating transcription factor 6; PERK, PKR-like endoplasmic reticulum kinase; MTSS1, MTSS I-bar domain containing 1; HBP1, HMG-box transcription factor 1; KLF4, Kruppel-like factor 4; LPCAT1, Lysophosphatidylcholine acyltransferase 1; EFNA3, Ephrin A3; VEGFC, Vascular endothelial growth factor C; HIF1A, Hypoxia inducible factor 1 subunit alpha; AGK, Acylglycerol kinase; MMP9, Matrix metallopeptidase 9; EMT, Epithelial-mesenchymal transition; GNAS, GNAS complex locus; CRKL, CRK like proto-oncogene.

## 4 Therapeutic strategies targeting epigenetic mechanisms in OSCC

The dynamic and reversible nature of epigenetic alterations presents a unique therapeutic avenue for OSCC (3). Unlike genetic mutations, these changes can be potentially reversed by pharmacological agents or targeted molecular strategies, thereby restoring normal gene expression patterns and suppressing tumor growth (85). This understanding of the underlying mechanism has spurred the investigation of two major therapeutic approaches, including conventional epi-drugs and novel bio-engineered delivery systems (193). Current efforts are concentrated on DNA methylation inhibitors and HDAC inhibitors, which aim to reverse the malignant phenotype (17, 194). Complementing this, the emerging field of engineered EVs offers a highly targeted method to deliver epigenetic therapeutics directly to tumor cells (195). The strategic objective involves using these agents, either as monotherapies or in rational combinations with conventional treatments, to overcome drug resistance and improve clinical outcomes for OSCC patients.

### 4.1 Epi-drugs for OSCC treatment

**Figure 9:**
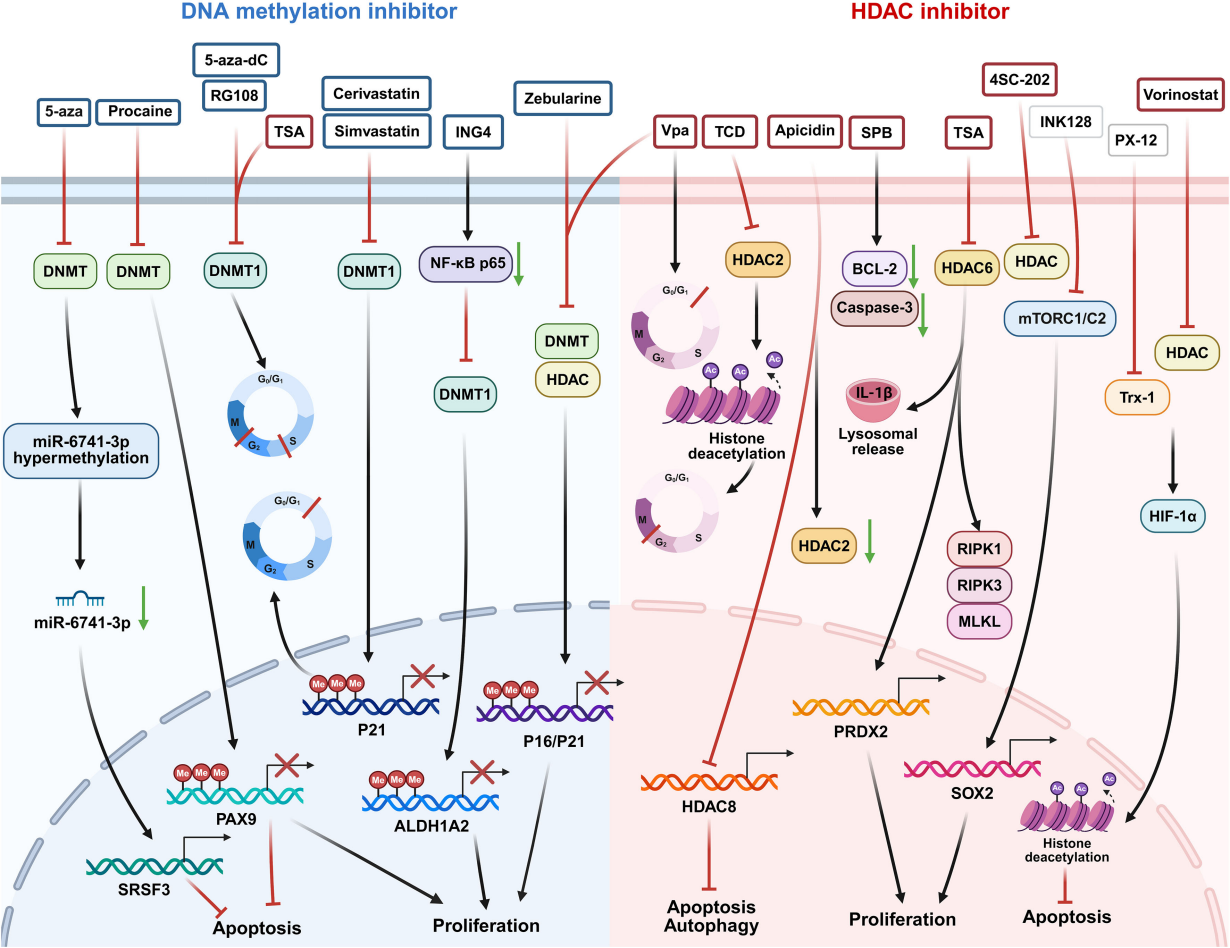
DNA methylation inhibitors and HDAC inhibitors as epi-drugs for OSCC. DNA methylation inhibitors functions primarily by inhibiting DNA methyltransferases (DNMTs). They directly reactivate silenced genes, synergize with other epigenetic or conventional therapies, and remodel the immunosuppressive tumor microenvironment (TME). This action reverses the aberrant hypermethylation of tumor suppressor genes and miRNAs, thereby leading to cancer cell apoptosis and impressing cell proliferation. HDAC inhibitors increase histone acetylation, thereby altering chromatin structure and gene expression. Key outcomes include induction of cell cycle arrest and apoptosis, inhibition of metastasis, and modulation of the TME. This action leads to cancer cell apoptosis, autophagy and impresses cell proliferation.

Epi-drugs represent the first generation of therapeutics designed to directly reverse aberrant epigenetic landscapes in cancer (196). Their mechanism of action involves the pharmacological inhibition of key enzymes responsible for DNA methylation (e.g., DNMTs) or histone deacetylation (e.g., HDACs), leading to the reactivation of silenced tumor suppressor genes and the repression of oncogenic pathways (197). In the context of OSCC, the most extensively investigated classes include DNMTs inhibitors and HDAC inhibitors (**Figure 9**) (15). The clinical potential of these agents is being explored both as single agents and, more promisingly, in combination with chemotherapy, radiotherapy, or other targeted drugs to synergistically enhance anti-tumor efficacy and circumvent resistance mechanisms (198).

#### 4.1.1 DNA methylation inhibitors

DNA methylation inhibitors represent a promising class of epi-drugs for OSCC. These agents primarily function by inhibiting DNMTs, thereby reversing the aberrant hypermethylation of tumor suppressor genes and restoring their anti-tumor functions (67). The exploration of these inhibitors spans from well-characterized drugs like 5-aza (5-azacytidine) to novel applications of existing compounds such as statins.

The foundational mechanism of DNA methylation inhibitors involves the reactivation of silenced tumor suppressor genes. Treatment with DNMT inhibitors like 5-aza leads to the demethylation and subsequent upregulation of critical tumor suppressive miRNAs. For instance, 5-aza treatment reactivated miR-6741-3p, which exerts its tumor suppressive function by directly targeting and downregulating the oncogene SRSF3 (199). Beyond nucleoside analogs, the local anesthetic procaine also acts as a DNMT inhibitor. It reactivated the differentiation gene PAX9 by suppressing DNMT activity, which in turn inhibited cell growth, triggered apoptosis, and reduced cancer stemness through an autophagy-dependent pathway both *in vitro* and *in vivo* (92). A key future direction is to delineate the upstream regulatory mechanism, specifically how nuclear factor E2-related factor-2 (NRF2) contributes to sustaining PAX9 expression and the ensuing antitumor response, which will provide a more complete understanding of procaine’s action.

Research has demonstrated that combining DNA methylation inhibitors with other therapeutic modalities can yield synergistic anti-cancer effects. The combination of DNMT inhibitors such as 5-aza-2’-deoxycytidine (5-aza-dC) or N-phthalyl-L-tryptophan (RG108) with the HDAC inhibitor TSA significantly decreased OSCC cell viability. This enhanced cytotoxicity was attributed to cell cycle arrest in the S and G2/M phases and an increase in DNA double-strand breaks (200). However, these findings from 2D culture models require validation in more physiologically relevant systems, such as 3D spheroids and *in vivo* models. Another study confirmed that the combination of the DNMT inhibitors zebularine and the HDAC inhibitors Valproic acid (Vpa) exhibited significant cytotoxic effects and upregulated tumor suppressor genes including P16 and P21 in OSCC cells. This combination also effectively reduced tumor volume in a xenograft mouse model of well-differentiated OSCC (198). Furthermore, the combination of 5-aza with spirulina-based photodynamic therapy (PDT) showed a synergistic effect, markedly enhancing cell death, apoptosis, and DNA damage while suppressing cell migration and invasion in OSCC cells (201). To advance these promising combination strategies, future efforts should focus on delineating the underlying signaling pathways and rigorously evaluating their efficacy and safety in advanced animal models and clinical trials. Additionally, exploring novel combinations, such as with immunotherapy, or developing more efficient photosensitizers could further unlock their full therapeutic potential.

Emerging evidence indicates that the antitumor effects of DNA methylation inhibition extend beyond cancer cells to modulate the TME. Inhibition of DNMT1 was found to improve the TME in immunocompetent mouse OSCC models. This improvement was characterized by a decrease in MDSCs and an increase in tumor-infiltrating T cells, which collectively contributed to delayed tumor growth (202). This suggests that DNMT1 inhibitors can potentiate antitumor immunity, offering a promising strategy for OSCC treatment.

Some therapeutic agents exert their anti-cancer effects in part through indirect inhibition of DNMTs. Statins, including cerivastatin and simvastatin, were shown to inhibit OSCC cell proliferation by suppressing DNMT1 expression. This suppression led to the upregulation of p21 and subsequent G0/G1 cell cycle arrest, highlighting a novel epigenetic mechanism underlying the anti-proliferative effect of statins (202). Additionally, the tumor suppressor inhibitor of growth family member 4 (ING4) was found to play a repressive role in OSCC by promoting the degradation of NF-κB p65, which subsequently reduced DNMT1 expression. This reduction in DNMT1 led to decreased methylation of the aldehyde dehydrogenase 1 family member A2 (ALDH1A2) gene, thereby inhibiting OSCC progression (203). These studies reveal an indirect epigenetic pathway through which certain agents inhibit OSCC by targeting DNMT activity.

Recent research aims to refine the application of DNMT1 targeting to enhance efficacy and minimize toxicity. A key study demonstrated that DNMT1 knockdown or inhibition in OSCC remodeled a specific global DNA hypomethylation pattern. This altered methylation landscape collaboratively hampered the activation of the PI3K/AKT and CDK2-Rb pathways while inactivating glycogen synthase kinase 3 beta (GSK3β). This targeted approach achieved superior anti-cancer efficacy *in vivo* compared to a PI3K inhibitor alone and circumvented the adverse effects such as blood glucose dysregulation associated with direct kinase inhibition (17).

In conclusion, DNA methylation inhibitors demonstrate considerable therapeutic potential against OSCC through multifaceted mechanisms. They directly reactivate silenced tumor suppressor genes and miRNAs, synergize with other epigenetic therapies or conventional treatments, and remarkably remodel the immunosuppressive TME. The ongoing exploration of DNA methylation inhibitors, both as monotherapies and as integral components of combination regimens, heralds a promising and evolving frontier in the precision epigenetic therapy of OSCC.

#### 4.1.2 HDAC inhibitors

HDAC inhibitors constitute a significant class of epi-drugs under investigation for OSCC (204). These agents function by increasing histone acetylation, which alters chromatin structure and gene expression, leading to various anti-tumor effects including cell cycle arrest, apoptosis induction, inhibition of metastasis, and modulation of the TME (29). Research spans from pan-HDAC inhibitors to isoform-selective agents, exploring their potential both as monotherapies and in combination regimens.

HDAC inhibitors demonstrate potent anti-proliferative and pro-apoptotic effects in OSCC models. Apicidin was shown to inhibit cell growth in murine OSCC cells both *in vitro* and *in vivo*, an effect linked to its selective suppression of HDAC8 expression and the subsequent induction of apoptosis and autophagy (205). Similarly, the natural compound trichodermin (TCD) suppressed OSCC cell proliferation, induced G2/M phase arrest, and triggered caspase-dependent apoptosis, also involving mitochondrial dysfunction and downregulation of HDAC-2 and its downstream signaling (206). Sodium phenylbutyrate (SPB) also inhibited OSCC cell viability and promoted apoptosis, accompanied by downregulation of the anti-apoptotic protein B-cell lymphoma 2 (BCL-2) and increased caspase-3 cleavage (207). These findings underscore that HDAC inhibitors counter OSCC through a convergence of mechanisms centered on arresting growth and inducing cell death.

Targeting specific HDAC isoforms, particularly HDAC6, has emerged as a promising therapeutic strategy. Pharmacological inhibition of HDAC6 by TSA was reported to overcome cisplatin resistance in OSCC by targeting CSCs. This effect was mediated through increased oxidative stress, DNA damage, and reduced expression of the antioxidant protein peroxiredoxin 2 (PRDX2) (208). Another study confirmed that HDAC6 inhibition with TSA attenuated tumor progression in a mouse model, which was associated with suppressed lysosomal exocytosis of the pro-tumorigenic cytokine IL-1β via tubulin acetylation (113). Furthermore, HDAC6 blockade with TSA was found to attenuate necroptotic signaling by suppressing the receptor interacting serine/threonine kinase 1 (RIPK1)/RIPK3/mixed lineage kinase domain like pseudokinase (MLKL) pathway, thereby reducing cell death and reprogramming the immunosuppressive TME to enhance anti-tumor immunity (209). These studies establish HDAC6 inhibition as a versatile therapeutic strategy that counteracts OSCC through multiple parallel mechanisms.

The combination of HDAC inhibitors with other therapeutic agents often yields superior anti-tumor efficacy (194). The co-administration of the Class I HDAC inhibitor 4SC-202 and the mTORC1/C2 inhibitor INK128 exhibited synergistic effects in suppressing OSCC cell growth, sphere formation, and tumor initiation. This combination repressed the expression of the stemness factor SOX2 through distinct mechanisms, namely miRNA-mediated mRNA degradation and inhibition of cap-dependent translation (29). The rationale for such drug conjugates was supported by the development of Roxyl-ZR, a single molecule combining HDAC and cyclin-dependent kinase (CDK) inhibitory motifs, which showed enhanced efficacy in suppressing OSCC proliferation and metastasis by inducing cell cycle arrest and apoptosis and inhibiting the JAK1/STAT3 pathway (210). Another synergistic interaction was observed between the HDAC inhibitor vorinostat and the Trx-1 inhibitor PX-12 under hypoxic conditions, which was more effective than either agent alone and was linked to specific alterations in histone acetylation and methylation marks (106, 211). Together, these findings demonstrate that HDAC inhibitor-based combinations exert synergistic anti-tumor effects in OSCC by engaging multiple targets and pathways.

HDAC inhibitors also show potential in targeting CSCs and overcoming therapy resistance (212). Research indicated that the HDAC inhibitor vorinostat and the NF-κB inhibitor emetine could both disrupt the CSC subpopulation in OSCC when used individually. Their combined administration did not yield further enhancement, suggesting that each agent independently targets CSCs through distinct pathways, namely increasing histone acetylation and inhibiting NF-κB signaling, respectively (213). Vpa was found to inhibit OSCC cell viability, induce G1 cell cycle arrest, and promote apoptosis, effects that were associated with the regulation of SUMOylation in both *in vitro* and *in vivo* models (214). The combination of the DNMT inhibitor zebularine and the HDAC inhibitor vpa also demonstrated significant cytotoxic effects and upregulated tumor suppressor genes in OSCC cells, effectively reducing tumor volume *in vivo* (198). However, as the xenograft model used cannot fully recapitulate the human oral cancer TME, future studies employing more representative models are warranted to strengthen the clinical relevance of these promising findings.

In conclusion, HDAC inhibitors demonstrate multi-faceted anti-tumor efficacy in OSCC, ranging from direct cytotoxicity and metastasis inhibition to overcoming chemoresistance and modulating immunosuppressive TME. The emergence of isoform-selective inhibitors, particularly targeting HDAC6, and rational combination strategies represent a promising direction for future therapeutic development.

### 4.2 Engineered EVs for OSCC treatment

**Figure 10:**
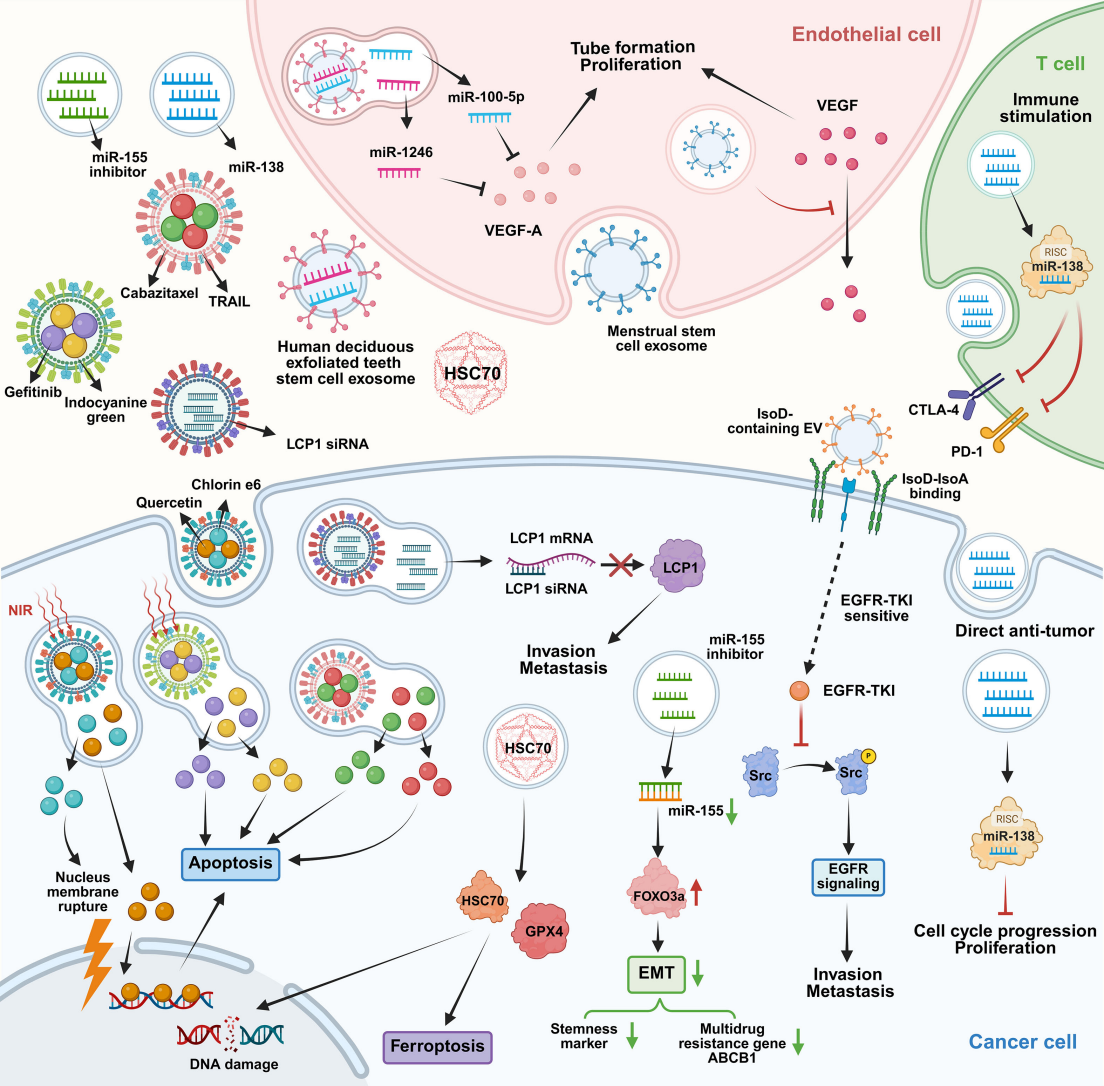
Engineered EVs for OSCC treatment. EVs can be loaded with diverse therapeutic cargoes, such as small-molecule drugs, miRNAs, siRNAs and proteins. In epigenetic therapy, EVs deliver drugs with improved bioavailability or transport tumor−suppressive miRNAs/siRNAs to directly reprogram the epigenetic circuitry of cancer cells, offering a precise and potent strategy for epigenetic modulation.

Building upon the foundation of epi-drugs, engineered EVs have emerged as a next-generation platform for targeted epigenetic therapy (215). EVs are natural nanoscale carriers that can be loaded with various therapeutic cargoes, such as small molecule drugs, RNAs (e.g., miRNAs, siRNAs), or proteins (216). By engineering their surface, EVs can be endowed with high specificity for OSCC cells, minimizing off-target effects (217). In the context of epigenetic therapy, EVs are being leveraged to deliver specific drugs with improved bioavailability, or to transport specific tumor-suppressive miRNAs and siRNAs that can directly reprogram the epigenetic circuitry of cancer cells (218). This approach holds significant promise for achieving precise and potent epigenetic modulation in OSCC (**Figure 10**).

One primary strategy involves using EVs as sophisticated delivery vehicles for small molecule drugs and nucleic acids. For instance, EVs co loaded with the photosensitizer chlorin e6 and the flavonoid quercetin achieved precise tumor targeting and light activated drug release effectively inhibiting OSCC proliferation (219). Similarly tumor exosome-based nanoparticles co formulating indocyanine green and gefitinib demonstrated deep tumor penetration and synergistic photothermal and molecular targeted therapy leading to efficient tumor ablation (220). Another study highlighted the use of tetrahedral DNA nanostructure modified EVs which showed improved biological stability and delivery efficiency ultimately inducing ferroptosis and DNA damage in OSCC cells by targeting glutathione peroxidase 4 (GPX4) (221). Furthermore, mesenchymal stem cell derived exosomes loaded with cabazitaxel and TNF-related apoptosis-inducing ligand (TRAIL) exhibited a powerful synergistic effect inducing apoptosis in drug resistant OSCC cells (222). Additionally, engineering exosomes to deliver specific tumor suppressive RNAi such as siRNA against lymphocyte cytosolic protein 1 (LCP1) has been proven to attenuate oral cancer progression effectively (223). These studies collectively underscore the efficacy of engineered EVs in enhancing drug delivery and enabling potent combination therapies.

A particularly promising application of engineered EVs lies in overcoming drug resistance and modulating the TME. Exosomes loaded with a miR-155 inhibitor were shown to reverse cisplatin resistance in OSCC by upregulating FOXO3a and suppressing stem like properties in tumor cells (224). In the realm of immunotherapy, γδ T cell derived EVs delivering miR-138 not only directly inhibited OSCC tumor growth but also stimulated CD8^+^ T cell activity by targeting immune checkpoints like PD-1 and CTLA-4. This dual function highlights the potential of EV based platforms to simultaneously target cancer cells and enhance anti-tumor immunity (225). Another innovative approach used EVs containing a secreted EGFR isoform IsoD as a co drug to sensitize traditionally TKI resistant squamous cell cancers to TKI by altering endosomal signaling (226). Thus, EVs offer a novel strategy to resensitize tumors to conventional treatments and potentiate immune responses.

Beyond their role as drug carriers, certain native or engineered EVs possess intrinsic anti-tumor properties, multiple studies have shown that exosomes derived from various stem cell sources can inhibit OSCC angiogenesis, a critical process for tumor growth. For example, menstrual stem cell exosomes directly reduced VEGF secretion from endothelial cells and caused loss of tumor vasculature *in vivo* (227). Similarly, exosomes from stem cells of human deciduous exfoliated teeth were enriched with miR-100-5p and miR-1246 and upon transfer to endothelial cells they suppressed tube formation and tumor growth by downregulating VEGF-A (228). This highlights that some EVs can function as multifaceted therapeutics themselves beyond mere delivery vehicles.

Despite the considerable promise the clinical translation of engineered EV therapies faces several hurdles. The standardization of isolation methods for large scale production remains a significant challenge as techniques like ultracentrifugation and size exclusion chromatography each present distinct advantages and drawbacks (229). Furthermore, achieving efficient cargo loading remains a major hurdle, as current methods often suffer from low efficiency and may compromise the structural integrity and biological functionality of EVs (230). Moreover, the long-term safety and potential immunogenicity of engineered EVs, especially upon repeated administration, demand rigorous evaluation, as engineering processes may alter their native composition and trigger adverse immune reactions (231). Addressing these issues of manufacturing consistency loading efficiency and biological safety is crucial for advancing engineered EVs from promising preclinical tools to viable clinical treatments for OSCC patients (232).

The mechanisms and functions of representative epigenetic therapies are summarized in **Table 3**. Combining these epigenetic-targeting strategies with standard treatments or immunotherapy presents a promising avenue for improving patient outcomes.

**Table 3:** Therapeutic methods for OSCC treatment.

Therapeutic method	Medicine	Target	Mechanism	Function	Ref.
DNA methylation inhibitor	5-Azacytidine	miR-6741-3p/SRSF3	Reactivates tumor-suppressor miRNAs, enabling identification of miR-6741-3p as a novel tumor suppressor targeting the oncogene SRSF3.	Reactivates tumor suppressor miRNAs.	(199)
		MMP-2/MMP-9	Synergizes with spirulina-based PDT to enhance antitumor effects in OSCC cells.	Suppresses cell viability, migration, and invasion.	(201)
	Procaine	PAX9	Leads to reactivation of the tumor suppressor gene PAX9.	Suppresses stemness and enhances chemosensitivity.	(92)
	5-Azacytidine /RG108	Caspase 3/7	Induces cell cycle arrest in S and G2/M phases and promotes DNA damage.	Suppresses tumor cell viability and leads to apoptosis.	(200)
	Zebularine	P16/P21/NPY/RASSF1	Synergizes with Valproic acid to reactivate silenced tumor suppressor genes by reducing their promoter DNA methylation	Reactivates tumor suppressor genes.	(198)
	Inhibition of DNMT1	P21	Reduces myeloid-derived suppressor cells and increases tumor-infiltrating T cells.	Delays tumor growth.	(202)
		PI3K-AKT/CDK2-Rb/GSK3β	Disrupts the coordinated activation of PI3K-AKT and CDK2-Rb signaling pathways and inactivates GSK3β through shRNA knockdown or pharmacological inhibition.	Promotes tumor suppression and circumvents toxicity.	(17)
	ING4	NF-κB p65/ALDH1A2	Leads to reduced methylation of the tumor suppressor gene ALDH1A2 and ING4-mediated promotion of NF-κB p65 ubiquitination and degradation.	Contributes to the suppression of tumor cell proliferation, migration, and invasion.	(203)
HDAC inhibitor	Apicidin	HDAC8	Induces cell growth inhibition and selectively reduces HDAC8 expression in AT-84 cells	Induces apoptosis, autophagy and inhibits cell proliferation *in vitro* and *in vivo*.	(205)
	TCD	MMP-9/HDAC-2	Downregulates MMP-9 and induces G2/M cell cycle arrest and caspase-dependent apoptosis and inhibiting HDAC-2 and downstream oncogenic signaling.	Suppresses tumor proliferation, migration, and invasion.	(206)
	SPB	BCL-2/caspase-3	Induces caspase-3-dependent apoptosis via BCL-2 downregulation.	Suppresses tumor proliferation.	(207)
	Tubastatin A	PRDX2	Reduces PRDX2 to disrupt HDAC6-mediated oxidative stress suppression.	Induces apoptosis and CSCs stemness phenotype, and reverses immunosuppression.	(208)
		IL-1β	Disrupts IL-1β-driven malignancy and immune evasion by targeting tubulin-dependent secretory pathways and reversing immunosuppressive cell populations in the TME.		(113)
		RIPK1/RIPK3/MLKL	Suppresses OSCC progression through inhibiting RIPK1/RIPK3/MLKL-mediated necrotic cell death by blocking phosphorylated MLKL translocation to the cell membrane.		(209)
	4SC-202	mTORC1/C2/SOX2	Inhibits ALDH1(+) CSCs through downregulating SOX2 via miR-429/miR-1181-mediated mRNA degradation.	Overcomes chemoresistance and suppresses tumor growth.	(29)
	Roxyl-ZR	CDK/JAK1-STAT3	Blocks tumor growth in xenograft models with low systemic toxicity through JAK1-STAT3 signaling pathway inhibition.	Suppresses tumor growth.	(210)
	Vorinostat	HIF-1α/Trx-1	Is combined with the Trx-1 inhibitor PX-12 to target HIF-1α stability.	Leads to ROS-mediated apoptosis and reduces the drug concentration.	(106, 211)
	SAHA	NF-κB	Is combined with the NF-κB inhibitor emetine to target therapy-resistant cells.	Disrupts CSCs.	(213)
	Valproic acid	P16/P21/NPY/RASSF1	Synergizes with Zebularine to reactivate tumor suppressor genes.	Induces cytotoxic effects and reduces tumor growth.	(198)
		SUMO1/SUMO2	Induces G1 phase cell cycle arrest and significantly upregulates SUMO1/SUMO2 expression.		(214)
Engineered EVs	Photosensitizer chlorin e6/Flavonoid quercetin	ROS	Synergizes intrinsic tumor tropism with photo-triggered release of natural anticancer compounds to enable light-activated drug release, triggering rapid quercetin release.	Inhibits proliferation and promotes apoptosis.	(219)
	Indocyanine green/Gefitinib	ROS	Utilizes passive and homologous targeting to achieve deep tumor accumulation and penetration.	Leads to potent inhibition of proliferation and angiogenesis.	(220)
	Tetrahedral DNA nanostructure modified extracellular vesicle	Hsc70/GPX4	Combines enhanced biodistribution with Hsc70 protein delivery to drive GPX4 degradation.	Leads to potent inhibition of tumor proliferation and migration.	(221)
	Cabazitaxel/TRAIL	CTX/TRAIL	Inhibits the PI3K/AKT/mTOR signaling pathway via CTX release and induces apoptosis via TRAIL-mediated activation of death receptors.	Overcomes drug resistance and enhances tumor suppression.	(222)
	siRNA against LCP1	LCP1	Enables targeted siRNA delivery for precise silencing of the LCP1 oncogene.	Provides a tumor-specific gene therapy platform.	(223)
	miR-155 inhibitor	FOXO3a	Upregulates FOXO3a and induces mesenchymal-to-epithelial transition. Additionally, the treatment suppresses CSC properties and drug efflux transporter expression.	Restores cisplatin sensitivity and overcomes key mechanisms of chemoresistance.	(224)
	IsoD	TKI	Transfers exquisite sensitivity from responsive to TKI-resistant tumor cells to TKIs.	Reprograms cellular signaling that bypasses conventional resistance mechanisms.	(226)
	Menstrual stem cell exosomes	VEGF	Triggers dose-dependent cytotoxicity in endothelial cells and suppresses VEGF secretion.	Disrupts tumor vasculature, resulting in tumor suppression.	(227)
	miR-100-5p/miR-1246	VEGFA/MMP-9/ANGPT1	Represses pro-angiogenic factors, leading to downregulation of VEGFA, MMP-9, and ANGPT1.	Inhibits endothelial cell proliferation, migration, and tube formation while promoting apoptosis.	(228)

SRSF3, Serine/arginine-rich splicing factor 3; MMP-2/9, Matrix metallopeptidase 2/9; PDT, Photodynamic therapy; PAX9, Paired box 9; RG108, N-Phthalyl-L-tryptophan; NPY, Neuropeptide y; RASSF1, Ras association domain family member 1; DNMT1, DNA methyltransferase1; PI3K-AKT, Phosphoinositide 3-kinase - AKT serine/threonine kinase; CDK2-Rb, Cyclin-dependent kinase 2 - retinoblastoma protein; GSK3β, Glycogen synthase kinase 3 beta; ING4, Inhibitor of growth family member 4; NF-κB p65, Nuclear factor kappa B subunit p65; ALDH1A2, Aldehyde dehydrogenase 1 family member A2; HDAC 8, Histone deacetylase 8 ; BCL-2, B-cell lymphoma 2; PRDX2, Peroxiredoxin 2; IL-1β, Interleukin-1 beta; TME, Tumor environment; RIPK1, Receptor interacting serine/threonine kinase 1; RIPK3, Receptor interacting serine/threonine kinase 3; MLKL, Mixed lineage kinase domain like pseudokinase; mTORC1/2, Mechanistic target of rapamycin complex 1/2; SOX2, SRY-box transcription factor 2; CSC, Cancer stem cell; CDK, Cyclin-dependent kinase; JAK1-STAT3, Janus kinase 1 - signal transducer and activator of transcription 3; HIF-1α, Hypoxia inducible factor 1 subunit alpha; Trx-1, Thioredoxin-1; SUMO1/2, Small Ubiquitin Like Modifier 1/2; ROS, Reactive Oxygen Species; Hsc70, Heat shock cognate 70; GPX4, Glutathione peroxidase 4; CTX, Cabazitaxel; TRAIL, Tumor necrosis factor-related apoptosis-inducing ligand; LCP1, Lymphocyte cytosolic protein 1; FOXO3a, Forkhead box O3a; TKI, tyrosine kinase inhibitors; VEGF, Vascular endothelial growth factor; MMP-9, Matrix metallopeptidase 9; ANGPT1, Angiopoietin 1.

## 5 Epigenetics behind drug resistance in OSCC

Emerging evidence underscores the critical contribution of epigenetic alterations to the development of drug resistance in various cancers, including OSCC (233). These heritable changes in gene expression, which occur without alterations to the DNA sequence itself, are now recognized as fundamental drivers in a subset of cancer cells known as drug-tolerant persister (DTP) cells. These cells survive initial drug exposure within a heterogeneous tumor population and can evolve increased tolerance to anticancer therapies (234). A key mechanism underpinning this survival is the maintenance of CSCs, a population notorious for its inherent resistance to conventional treatments (235). Cancer cells dynamically reshape their epigenomic landscape through processes such as DNA methylation, histone modifications, and the action of ncRNAs. This reprogramming enables them to activate diverse mechanisms to evade therapeutic attacks (236). Central to this process are epigenetic regulators, including DNMTs, chromatin remodeling complexes, and various histone modifiers, alongside ncRNA (237). These factors collectively fine-tune the expression of genes involved in multiple forms of therapy resistance. Consequently, a deep understanding of these epigenetic mechanisms provides promising avenues for overcoming drug resistance and enhancing cancer treatment efficacy in OSCC.

### 5.1 DNA methylation and drug resistance in OSCC

DNA hypomethylation fosters a resistant phenotype primarily by activating pro-survival signaling and shaping an immunosuppressive TME. A key example is the Sox11 gene, whose hypomethylation leads to its overexpression and subsequent activation of the PI3K/AKT signaling pathway. The constitutive activation of this pathway is a well-established mechanism that confers resistance to a broad spectrum of anticancer drugs by enhancing cell survival and proliferation (73). Beyond specific oncogenes, widespread hypomethylation can remodel the immune landscape. For instance, the hypomethylation of immune-related lncRNAs, such as MEG3, contributes to an immunosuppressive TME characterized by the enrichment of regulatory T cells and MDSCs. This immunological state is a principal cause of resistance to immune checkpoint inhibitor therapy (77).

Conversely, DNA hypermethylation promotes resistance by transcriptionally silencing genes essential for drug response. The epigenetic inactivation of tumor suppressor genes plays a direct role in this process. For example, hypermethylation-induced silencing of the pro-apoptotic gene HOXA5 diminishes the cell death response, thereby blunting the efficacy of chemotherapeutic agents like cisplatin. Functional studies confirm that reactivating HOXA5 can significantly enhance cisplatin-induced cytotoxicity, positioning it as a key mediator of chemoresistance (91). Furthermore, hypermethylation can target DNA repair genes, creating a dependency that can be exploited. The silencing of genes like O-6-methylguanine-DNA methyltransferase (MGMT) and mutL homolog 1 (MLH1) alters the cellular response to genotoxic stress. This forms the mechanistic basis for the efficacy of combining demethylating agents, such as 5-aza-dC, with cisplatin. This combination therapy has been shown to sensitize OSCC cells to chemotherapy by reversing the epigenetic blockade of these critical pathways, offering a promising strategy to overcome chemoresistance (238). However, as 5-aza-dC acts as a global demethylating agent, the precise causal relationship between the re-expression of specific DNA repair genes and the restored chemosensitivity remains to be fully elucidated. Future studies employing more targeted epigenetic editing tools are warranted to definitively establish the mechanistic links.

In conclusion, DNA methylation aberrations are versatile drivers of drug resistance in OSCC. Hypermethylation silences tumor suppressors and apoptotic agents, while hypomethylation activates oncogenic signaling and fosters an immunosuppressive milieu. Therefore, profiling DNA methylation patterns holds significant promise for predicting therapeutic response and developing novel strategies to overcome drug resistance in OSCC patients.

### 5.2 Histone modifications and drug resistance in OSCC

While the role of histone modifications in driving drug resistance is well-established in many cancers, direct mechanistic evidence in OSCC remains limited. A key study by de Castro et al. (2024) identified the TNF-α/NFκB/sirtuin 1 (SIRT1) axis as a contributor to cisplatin resistance in OSCC, where SIRT1-mediated histone deacetylation facilitates the expansion of CSCs, a known reservoir for therapy-resistant cells. Pharmacological inhibition of this pathway reduced CSC populations and resensitized tumors to treatment (239).

Beyond this example in OSCC, a wealth of evidence from other malignancies highlights the versatility of histone modifications in conferring therapy resistance. For instance, the histone demethylase lysine demethylase 5A (KDM5A) promotes resistance to temozolomide in glioblastoma and to tamoxifen in breast cancer by erasing the active H3K4me3 mark from the promoters of tumor suppressor and cell cycle arrest genes (240). Similarly, the methyltransferase EZH2, which catalyzes the repressive H3K27me3 mark, drives cisplatin resistance in ovarian cancer (241), and gefitinib resistance in non-small cell lung cancer (242) by silencing key pro-apoptotic and differentiation pathways. Conversely, the loss of H3K27me3 demethylases like KDM6A/UTX can impair the response to cytarabine in acute myeloid leukemia by altering the expression of drug transporters (243).

In conclusion, the current understanding of histone modification-mediated drug resistance in OSCC is nascent, with the SIRT1 axis representing a promising but isolated finding (239). Future research should actively bridge this knowledge gap by systematically profiling histone marks in therapy-resistant OSCC. Investigations should explore the roles of understudied writers, erasers, and readers of histone modifications, with a particular focus on their interplay in sustaining CSC phenotypes, repressing apoptosis, and regulating drug metabolism (244). Given the success of epigenetic combination therapies in other cancers, rational strategies that simultaneously target multiple histone modification pathways, or combine histone-modifying agents with conventional chemotherapy, hold significant potential to overcome resistance and improve outcomes for OSCC patients (245).

### 5.3 ncRNAs and drug resistance in OSCC

ncRNAs have emerged as central regulators in the development of therapy resistance in OSCC. They orchestrate complex networks that confer therapy resistance in OSCC by regulating critical pathways involving apoptosis, drug efflux, EMT, stemness, autophagy, and TME (246).

A prevalent mechanism is the ceRNA network. For instance, the circRNA circ-ILF2 promotes cisplatin resistance by sponging miR-1252 to upregulate KLF transcription factor 8 (KLF8), while simultaneously inducing M2 macrophage polarization to foster an immunosuppressive niche (247). Similarly, the lncRNA CYTOR drives cisplatin resistance and EMT by sequestering miR-1252-5p and miR-3148, leading to LPP upregulation (248). However, the upstream mechanisms responsible for forkhead box D1 (FOXD1) upregulation in OSCC remain unclear. Elucidating the cause of FOXD1 overexpression, whether genetic, epigenetic, or driven by other signaling pathways, will be a critical focus for future research. Moreover, the lncRNA OIP5 antisense RNA 1 (OIP5-AS1) also contributes to cisplatin resistance through a ceRNA mechanism, sponging miR-27b-3p to enhance tripartite motif containing 14 (TRIM14) expression (249). Collectively, these findings underscore the pivotal role of ceRNA networks as a fundamental and recurring mechanism underpinning drug resistance in OSCC.

ncRNAs critically regulate cell death pathways to enable survival under therapeutic stress. They can directly suppress apoptosis, as demonstrated by the lncRNA CEBPA divergent transcript (CEBPA-DT), which promotes cisplatin resistance by modulating the CEBPA-BCL2 mediated apoptosis pathway (250). Conversely, some ncRNAs can enhance sensitivity by promoting alternative cell death pathways. The circRNA circ-PKD2 is induced by cisplatin and sensitizes OSCC cells by promoting Atg13-mediated autophagy and apoptosis through sponging miR-646 (251). The critical translational step is to determine whether circ-PKD2 expression in patient tumors correlates with improved cisplatin sensitivity and thus holds predictive value. Furthermore, the circRNA CircAP1M2 activates autophagy related 9A (ATG9A)-associated autophagy to promote cisplatin resistance by inhibiting miR-1249-3p (252), highlighting the context-dependent role of ncRNA-regulated cell death in drug response.

The regulation of drug transport and intercellular communication is another pivotal mechanism of ncRNA-mediated resistance. ncRNAs can enhance the expression of drug efflux pumps to reduce intracellular drug accumulation. The circRNA circ_0109291 contributes to cisplatin resistance by acting as a sponge for miR-188-3p, leading to increased expression of the efflux transporter ATP binding cassette subfamily B member 1 (ABCB1) (253). Beyond cell-autonomous mechanisms, ncRNAs are actively shuttled within the TME via EVs to disseminate resistance. Exosomes derived from cisplatin-resistant OSCC cells are enriched with miR-21 and can transfer this miRNA to sensitive cells, conferring resistance by targeting the tumor suppressor genes PTEN and programmed cell death 4 (PDCD4) (254).

TME serves as a critical arena where ncRNAs orchestrate therapy resistance by mediating pro-tumorigenic crosstalk. Within cancer cells, ncRNAs can reinforce a therapy-resistant phenotype by sustaining stem-like populations. For instance, the lncRNA HOXA11-AS maintains the stemness of CD133-positive CSCs and reduces radiosensitivity by targeting the miR-518a-3p/PDK1 pathway (139). More broadly, stromal components in the TME actively export resistance via ncRNAs. A key example involves CAFs, which secrete exosomes carrying the lncRNA RORA antisense RNA 1 (RORA-AS1). Upon transferring to OSCC cells, this lncRNA promotes radiotherapy resistance by activating the IFITM1/STAT signaling cascade (255). To establish the clinical relevance of this intercellular communication, future work should validate the correlation and spatial co-localization between RORA-AS1 and CAFs in larger clinical cohorts. This intercellular communication underscores how ncRNAs derived from both tumor and stromal cells collaboratively forge an immunosuppressive and treatment-resistant niche.

In summary, ncRNAs form a tight ceRNA network that controls OSCC drug resistance by governing apoptosis, drug efflux, EMT, stemness, autophagy and TME. Their dysregulation, whether through cell-intrinsic expression changes or via exosomal transfer between cancer cells and stromal components, drives and spreads resistance, making these RNA nodes promising therapeutic targets to avert treatment failure and improve outcomes for OSCC patients.

## 6 Conclusion and future perspectives

Epigenetic dysregulation is now established as a fundamental driver of OSCC pathogenesis, intricately involved in tumor initiation, progression, metastasis, and the development of therapy resistance. This review has detailed the pivotal roles of DNA methylation, histone modifications, and ncRNAs in orchestrating these malignant processes. These mechanisms collectively reshape the cellular identity of OSCC by silencing tumor suppressor genes, activating oncogenic pathways, and critically, by dynamically molding an immunosuppressive TME. The reversible nature of these epigenetic alterations presents a compelling therapeutic opportunity, positioning epi-drugs as promising agents for OSCC management.

Substantial preclinical evidence shows that epigenetic therapies, including DNMT and HDAC inhibitors, can effectively halt the progression of OSCC. These agents demonstrate multi-faceted anti-tumor effects, from inducing apoptosis and cell cycle arrest to inhibiting invasion and metastasis (15). A particularly promising strategy is the development of isoform-selective inhibitors, such as those targeting HDAC6, which have shown potential in overcoming chemoresistance by targeting CSCs and reprogramming the immunosuppressive TME (113). Furthermore, the rational combination of epi-drugs with each other, or with conventional chemotherapy, radiotherapy, or targeted agents, frequently yields synergistic effects, offering a viable path to enhance therapeutic efficacy and counteract resistance. Considering the pivotal role of TME in driving therapy resistance, the development of therapeutic agents that directly target TME components should be prioritized. Future efforts may focus on strategies that disrupt the immunosuppressive and pro-tumorigenic networks within the TME, and rationally combine them with epigenetic therapies for enhanced efficacy (256, 257).

Despite this promise, the clinical translation of epigenetic therapies in OSCC faces significant challenges. A major impediment is the current lack of robust and reproducible epigenetic biomarkers validated in large, multi-center cohorts to guide patient stratification and predict therapeutic response (89). To systematically summarize the current landscape of epigenetic research in OSCC, **Table 4** consolidates key epigenetic biomarkers that have shown diagnostic or prognostic potential. Future research should prioritize the identification of OSCC-specific epigenetic vulnerabilities and the development of standardized biomarker detection platforms. Concurrently, advances in drug delivery systems, particularly nanoparticle-based vectors, are crucial to improve the tumor-specific delivery of epigenetic modulators while minimizing systemic toxicity (258).

**Table 4:** Diagnostic biomarkers and prognostic biomarkers in OSCC.

Clinical significance	Gene	Regulation	Epigenetic modulation	Function	Ref.
Diagnostic biomarker	miR-296	Upregulated	Hypomethylation of miR-296	As potential diagnostic biomarker, it reflects field cancerization and epigenetic instability.	(79)
	TERT	Upregulated	Hypomethylation of TERT		
	Fgf3	Upregulated	Hypomethylation of Fgf3	The early and specific hypomethylation of Fgf3 indicates its potential as a non-invasive or tissue-based molecular marker for identifying premalignant changes and early OSCC.	(80)
	TGM-3	Downregulated	Hypermethylation of TGM-3	The promoter hypermethylation-mediated epigenetic silencing is related to epithelial differentiation and formation of the cornified envelope.	(95)
	Hsa_circ_0009128	Upregulated	-	Hsa_circ_0009128 is upregulated in OSCC tissues and cell lines, and its expression level positively correlates with TNM staging and lymph node metastasis.	(183)
Prognostic biomarker	WISP1	Upregulated	Hypomethylation of WISP1	High WISP1 expression is linked to worse disease-specific survival (DSS; p=0.022) and reduced regional disease-free survival (RDFS; p=0.027)	(70)
	OAT	Upregulated	Hypomethylation of OAT	Hypermethylated OAT is predominantly found in radiation-sensitive tumor. Additionally, it is linked to increased infiltration of various stromal and immune cells in the tumor microenvironment which is associated with a worse prognosis.	(81)
	TPPP3	Upregulated	Hypomethylation of TPPP3	Overexpression of TPPP3 inhibits OSCC cell proliferation and migration in vitro, indicating its role as a tumor suppressor.	(82)
	lncRNA H19	Upregulated	Hypomethylation of lncRNA H19	OSCC patients with hypomethylated lncRNA H19 exhibit a significantly lower 5-year survival rate.	(83)
	miR-34b/c	Downregulated	Hypermethylation of miR-34b/c	Promoter hypermethylation of miR-34b/c, detected in a substantial subset of tumors, is associated with nodal involvement and predicts shorter overall survival.	(89)
	HOXA3	Downregulated	Hypermethylation of HOXA3	Hypermethylation of the HOXA3 3' UTR is strongly associated with higher tumor grade, advanced stage, poor differentiation, extranodal extension, and reduced overall survival.	(94)
	-	Upregulated	H3K9me3	High H3K9me3 levels associate with aggressive phenotypes, deeper tumor invasion, and poorer outcomes.	(109)
	-	Upregulated	H3K27me3	A high percentage of H3K27me3-positive tumor cells and high total H3K27me3 scores are significantly correlated with shorter overall survival in both univariate and multivariate analyses.	(110)
	TINCR	Downregulated	-	Patients with lower TINCR expression exhibit significantly worse overall survival, highlighting its strong correlation with disease progression and patient outcome.	(151)
	circ-OMAC	Upregulated	-	Its expression is significantly elevated in metastatic lymph nodes compared to primary tumors, and high levels of circ-OMAC correlate strongly with reduced overall survival in OSCC patients.	(184)

TERT, Telomerase reverse transcriptase; TGM-3, Transglutaminase 3; WISP1, WNT1-inducible signaling pathway protein 1; OAT, Ornithine aminotransferase; TPPP3, Tubulin polymerization promoting protein family member 3; HOXA3, Homeobox A3; TINCR, Terminal differentiation-induced non-coding RNA; circ-OMAC, circRNA-oral cancer metastasis-associated circRNA.

Looking ahead, the integration of multi-omics profiling, including single-cell epigenomics and spatial transcriptomics, into routine diagnostic workflows will be essential. These approaches will be pivotal for advancing biomarker-driven precision therapy in OSCC. Future therapeutic development should prioritize clinical trials that evaluate rational combinations of epi-drugs with conventional treatments or immunotherapy, guided by robust epigenetic and TME-based biomarkers to predict and monitor response. This will deepen our understanding of the dynamic epigenetic networks in OSCC and uncover novel actionable targets (259). The interplay between epigenetic reprogramming and the TME represents a fertile ground for therapeutic innovation, suggesting that combinations of epi-drugs with immune checkpoint inhibitors could be particularly effective (104). Ultimately, the successful clinical implementation of precision epigenetic therapy for OSCC will depend on multidisciplinary efforts to optimize drug design, validate predictive biomarkers, and intelligently integrate epigenetic strategies into multimodal treatment regimens.

## References

[1] Lechner M Liu J Masterson L Fenton TR . HPV-associated oropharyngeal cancer: epidemiology, molecular biology and clinical management. Nat Rev Clin Oncol. (2022) 19:306–27. doi: 10.1038/s41571-022-00603-7, PMID: 35105976 PMC8805140

[2] Wushou A Wang M Yibulayin F Feng L Lu MM Luo Y . Patients with cT1N0M0 oral squamous cell carcinoma benefit from elective neck dissection: A SEER-based study. J Natl Compr Canc Netw. (2021) 19:385–92. doi: 10.6004/jnccn.2020.7632, PMID: 33378738

[3] Tan Y Wang Z Xu M Li B Huang Z Qin S . Oral squamous cell carcinomas: state of the field and emerging directions. Int J Oral Sci. (2023) 15:44. doi: 10.1038/s41368-023-00249-w, PMID: 37736748 PMC10517027

[4] Yuan SF Chan LP Nguyen HDH Su CW Chen YK Chen JY . Areca nut-induced metabolic reprogramming and M2 differentiation promote OPMD Malignant transformation. J Exp Clin Cancer Res. (2024) 43:233. doi: 10.1186/s13046-024-03163-z, PMID: 39160581 PMC11334407

[5] Kinane DF Gabert J Xynopoulos G Guzeldemir-Akcakanat E . Strategic approaches in oral squamous cell carcinoma diagnostics using liquid biopsy. Periodontol 2000. (2024) 96:316–28. doi: 10.1111/prd.12567, PMID: 38676371 PMC11579816

[6] Jin N An Y Tian Y Zhang Z He K Chi C . Multispectral fluorescence imaging of EGFR and PD-L1 for precision detection of oral squamous cell carcinoma: a preclinical and clinical study. BMC Med. (2024) 22:342. doi: 10.1186/s12916-024-03559-w, PMID: 39183296 PMC11346054

[7] Zanoni DK Demétrio De Souza França P Valero C Peterson G Ardigo M Ghossein R . A prospective double-blinded comparison of reflectance confocal microscopy with conventional histopathology for *in vivo* assessment in oral cancer. Clin Cancer Res. (2024) 30:2486–96. doi: 10.1158/1078-0432.Ccr-23-1361, PMID: 38526414 PMC11145174

[8] Liu HM Xiong XP Yu ZL Shao Z Chen GL Liu YT . Neoadjuvant immunotherapy with or without chemotherapy in locally advanced oral squamous cell carcinoma: Randomized, two-arm, phase 2 trial. Cell Rep Med. (2025) 6:101930. doi: 10.1016/j.xcrm.2025.101930, PMID: 39889711 PMC11866509

[9] Wu CS Li HP Hsieh CH Lin YT Yi-Feng Chang I Chung AK . Integrated multi-omics analyses of oral squamous cell carcinoma reveal precision patient stratification and personalized treatment strategies. Cancer Lett. (2025) 614:217482. doi: 10.1016/j.canlet.2025.217482, PMID: 39842500

[10] Gan M Liu N Li W Chen M Bai Z Liu D . Metabolic targeting of regulatory T cells in oral squamous cell carcinoma: new horizons in immunotherapy. Mol Cancer. (2024) 23:273. doi: 10.1186/s12943-024-02193-7, PMID: 39696340 PMC11657557

[11] Liu YT Liu HM Ren JG Zhang W Wang XX Yu ZL . Immune-featured stromal niches associate with response to neoadjuvant immunotherapy in oral squamous cell carcinoma. Cell Rep Med. (2025) 6:102024. doi: 10.1016/j.xcrm.2025.102024, PMID: 40107247 PMC11970382

[12] Liu Z Zhang Z Zhang Y Zhou W Zhang X Peng C . Spatial transcriptomics reveals that metabolic characteristics define the tumor immunosuppression microenvironment via iCAF transformation in oral squamous cell carcinoma. Int J Oral Sci. (2024) 16:9. doi: 10.1038/s41368-023-00267-8, PMID: 38287007 PMC10824761

[13] de Visser KE Joyce JA . The evolving tumor microenvironment: From cancer initiation to metastatic outgrowth. Cancer Cell. (2023) 41:374–403. doi: 10.1016/j.ccell.2023.02.016, PMID: 36917948

[14] Kundu M Butti R Panda VK Malhotra D Das S Mitra T . Modulation of the tumor microenvironment and mechanism of immunotherapy-based drug resistance in breast cancer. Mol Cancer. (2024) 23:92. doi: 10.1186/s12943-024-01990-4, PMID: 38715072 PMC11075356

[15] Mesgari H Esmaelian S Nasiri K Ghasemzadeh S Doroudgar P Payandeh Z . Epigenetic regulation in oral squamous cell carcinoma microenvironment: A comprehensive review. Cancers (Basel). (2023) 15(23):49. doi: 10.3390/cancers15235600, PMID: 38067304 PMC10705512

[16] Gupta I Badrzadeh F Tsentalovich Y Gaykalova DA . Connecting the dots: investigating the link between environmental, genetic, and epigenetic influences in metabolomic alterations in oral squamous cell carcinoma. J Exp Clin Cancer Res. (2024) 43:239. doi: 10.1186/s13046-024-03141-5, PMID: 39169426 PMC11337877

[17] Liu Y Sun Y Yang J Wu D Yu S Liu J . DNMT1-targeting remodeling global DNA hypomethylation for enhanced tumor suppression and circumvented toxicity in oral squamous cell carcinoma. Mol Cancer. (2024) 23:104. doi: 10.1186/s12943-024-01993-1, PMID: 38755637 PMC11097543

[18] Yang W Zhou J Zhang Z Zhang K Xu Y Li L . Downregulation of lncRNA APCDD1L-AS1 due to DNA hypermethylation and loss of VHL protein expression promotes the progression of clear cell renal cell carcinoma. Int J Biol Sci. (2022) 18:2583–96. doi: 10.7150/ijbs.71519, PMID: 35414787 PMC8990466

[19] Wang X Wang C Yan G Kang Y Sun G Wang S . BAP18 is involved in upregulation of CCND1/2 transcription to promote cell growth in oral squamous cell carcinoma. EBioMedicine. (2020) 53:102685. doi: 10.1016/j.ebiom.2020.102685, PMID: 32113162 PMC7047197

[20] Ryu HY Hochstrasser M . Histone sumoylation and chromatin dynamics. Nucleic Acids Res. (2021) 49:6043–52. doi: 10.1093/nar/gkab280, PMID: 33885816 PMC8216275

[21] Vymetalkova V Vodicka P Vodenkova S Alonso S Schneider-Stock R . DNA methylation and chromatin modifiers in colorectal cancer. Mol Aspects Med. (2019) 69:73–92. doi: 10.1016/j.mam.2019.04.002, PMID: 31028771

[22] He Y Jiang X Duan L Xiong Q Yuan Y Liu P . LncRNA PKMYT1AR promotes cancer stem cell maintenance in non-small cell lung cancer via activating Wnt signaling pathway. Mol Cancer. (2021) 20:156. doi: 10.1186/s12943-021-01469-6, PMID: 34856993 PMC8638142

[23] Yang J Shi X Yang M Luo J Gao Q Wang X . Glycolysis reprogramming in cancer-associated fibroblasts promotes the growth of oral cancer through the lncRNA H19/miR-675-5p/PFKFB3 signaling pathway. Int J Oral Sci. (2021) 13:12. doi: 10.1038/s41368-021-00115-7, PMID: 33762576 PMC7991655

[24] Imam MS Abdel-Sattar RM Aldekhail NM Humaish NKA Gary SAG Alkhulaifi MAM . Cuproptosis in lung cancer: a nexus of ncRNA regulation, epigenetics, and tumor microenvironment Remodeling. Clin Exp Med. (2025) 26(1):17. doi: 10.1007/s10238-025-01903-9, PMID: 41335135 PMC12675717

[25] Jang TH Huang WC Tung SL Lin SC Chen PM Cho CY . MicroRNA-485-5p targets keratin 17 to regulate oral cancer stemness and chemoresistance via the integrin/FAK/Src/ERK/β-catenin pathway. J BioMed Sci. (2022) 29:42. doi: 10.1186/s12929-022-00824-z, PMID: 35706019 PMC9202219

[26] Huang Z Rui X Yi C Chen Y Chen R Liang Y . Silencing LCN2 suppresses oral squamous cell carcinoma progression by reducing EGFR signal activation and recycling. J Exp Clin Cancer Res. (2023) 42:60. doi: 10.1186/s13046-023-02618-z, PMID: 36899380 PMC10007849

[27] Chakraborty AK Raut RD Iqbal K Choudhury C Alhousami T Chogle S . Lysine-specific demethylase 1 controls key OSCC preneoplasia inducer STAT3 through CDK7 phosphorylation during oncogenic progression and immunosuppression. Int J Oral Sci. (2025) 17:31. doi: 10.1038/s41368-025-00363-x, PMID: 40246812 PMC12006301

[28] Shiah SG Hsiao JR Chang HJ Hsu YM Wu GH Peng HY . MiR-30a and miR-379 modulate retinoic acid pathway by targeting DNA methyltransferase 3B in oral cancer. J BioMed Sci. (2020) 27:46. doi: 10.1186/s12929-020-00644-z, PMID: 32238162 PMC7114797

[29] Liang X Deng M Zhang C Ping F Wang H Wang Y . Combined class I histone deacetylase and mTORC1/C2 inhibition suppresses the initiation and recurrence of oral squamous cell carcinomas by repressing SOX2. Cancer Lett. (2019) 454:108–19. doi: 10.1016/j.canlet.2019.04.010, PMID: 30981761

[30] Yang C Xue Y Duan Y Mao C Wan M . Extracellular vesicles and their engineering strategies, delivery systems, and biomedical applications. J Control Release. (2024) 365:1089–123. doi: 10.1016/j.jconrel.2023.11.057, PMID: 38065416

[31] Di Ianni E Obuchi W Breyne K Breakefield XO . Extracellular vesicles for the delivery of gene therapy. Nat Rev Bioengineering. (2025) 3:360–73. doi: 10.1038/s44222-025-00277-7, PMID: 41104040 PMC12526208

[32] Li C Dong X Li B . Tumor microenvironment in oral squamous cell carcinoma. Front Immunol. (2024) 15:1485174. doi: 10.3389/fimmu.2024.1485174, PMID: 39744628 PMC11688467

[33] Cabral LGS Martins IM Paulo EPA Pomini KT Poyet JL Maria DA . Molecular mechanisms in the carcinogenesis of oral squamous cell carcinoma: A literature review. Biomolecules. (2025) 15(5):30. doi: 10.3390/biom15050621, PMID: 40427514 PMC12109257

[34] Qiao Y Yin H Zhang Y Zhang J Dong Q . Domestication and feedback: bidirectional hijacking in pancreatic ductal adenocarcinoma microenvironment. Front Immunol. (2025) 16:1585858. doi: 10.3389/fimmu.2025.1585858, PMID: 40861469 PMC12375688

[35] Xu J Ding L Mei J Hu Y Kong X Dai S . Dual roles and therapeutic targeting of tumor-associated macrophages in tumor microenvironments. Signal Transduct Target Ther. (2025) 10:268. doi: 10.1038/s41392-025-02325-5, PMID: 40850976 PMC12375796

[36] Chen S Wang M Lu T Liu Y Hong W He X . JMJD6 in tumor-associated macrophage regulates macrophage polarization and cancer progression via STAT3/IL-10 axis. Oncogene. (2023) 42:2737–50. doi: 10.1038/s41388-023-02781-9, PMID: 37567973 PMC10491492

[37] Shi X Yang J Deng S Xu H Wu D Zeng Q . TGF-β signaling in the tumor metabolic microenvironment and targeted therapies. J Hematol Oncol. (2022) 15:135. doi: 10.1186/s13045-022-01349-6, PMID: 36115986 PMC9482317

[38] Wu F Yang J Liu J Wang Y Mu J Zeng Q . Signaling pathways in cancer-associated fibroblasts and targeted therapy for cancer. Signal Transduct Target Ther. (2021) 6:218. doi: 10.1038/s41392-021-00641-0, PMID: 34108441 PMC8190181

[39] Jia H Chen X Zhang L Chen M . Cancer associated fibroblasts in cancer development and therapy. J Hematol Oncol. (2025) 18:36. doi: 10.1186/s13045-025-01688-0, PMID: 40156055 PMC11954198

[40] Huang CX Lao XM Wang XY Ren YZ Lu YT Shi W . Pericancerous cross-presentation to cytotoxic T lymphocytes impairs immunotherapeutic efficacy in hepatocellular carcinoma. Cancer Cell. (2024) 42:2082–97.e10. doi: 10.1016/j.ccell.2024.10.012, PMID: 39547231

[41] Tay C Tanaka A Sakaguchi S . Tumor-infiltrating regulatory T cells as targets of cancer immunotherapy. Cancer Cell. (2023) 41:450–65. doi: 10.1016/j.ccell.2023.02.014, PMID: 36917950

[42] Lasser SA Ozbay Kurt FG Arkhypov I Utikal J Umansky V . Myeloid-derived suppressor cells in cancer and cancer therapy. Nat Rev Clin Oncol. (2024) 21:147–64. doi: 10.1038/s41571-023-00846-y, PMID: 38191922

[43] De Martino D Bravo-Cordero JJ . Collagens in cancer: structural regulators and guardians of cancer progression. Cancer Res. (2023) 83:1386–92. doi: 10.1158/0008-5472.Can-22-2034, PMID: 36638361 PMC10159947

[44] Spada S Tocci A Di Modugno F Nisticò P . Fibronectin as a multiregulatory molecule crucial in tumor matrisome: from structural and functional features to clinical practice in oncology. J Exp Clin Cancer Res. (2021) 40:102. doi: 10.1186/s13046-021-01908-8, PMID: 33731188 PMC7972229

[45] Prabhudesai SA Carvalho K Dhupar A Spadigam A . Elastin remodeling: Does it play a role in priming the Malignant phenotype of oral mucosa? Indian J Pathol Microbiol. (2023) 66:332–8. doi: 10.4103/ijpm.ijpm_512_21, PMID: 37077077

[46] Amorim S Reis CA Reis RL Pires RA . Extracellular matrix mimics using hyaluronan-based biomaterials. Trends Biotechnol. (2021) 39:90–104. doi: 10.1016/j.tibtech.2020.06.003, PMID: 32654775

[47] Zheng B Han Y Zhang H . Role of matrix metalloproteinases in the invasion of glioblastoma and drug interventions (Review). Int J Mol Med. (2026) 57(2):17. doi: 10.3892/ijmm.2025.5704, PMID: 41347826 PMC12695158

[48] Wang H Wang T Yan S Tang J Zhang Y Wang L . Crosstalk of pyroptosis and cytokine in the tumor microenvironment: from mechanisms to clinical implication. Mol Cancer. (2024) 23:268. doi: 10.1186/s12943-024-02183-9, PMID: 39614288 PMC11607834

[49] Song S Zhang G Chen X Zheng J Liu X Wang Y . HIF-1α increases the osteogenic capacity of ADSCs by coupling angiogenesis and osteogenesis via the HIF-1α/VEGF/AKT/mTOR signaling pathway. J Nanobiotechnology. (2023) 21:257. doi: 10.1186/s12951-023-02020-z, PMID: 37550736 PMC10405507

[50] Wang X Li L Zhao K Lin Q Li H Xue X . A novel LncRNA HITT forms a regulatory loop with HIF-1α to modulate angiogenesis and tumor growth. Cell Death Differ. (2020) 27:1431–46. doi: 10.1038/s41418-019-0449-8, PMID: 31700144 PMC7205893

[51] López-Graniel CM de León DT Meneses-García A Gómez-Ruiz C Frías-Mendivil M Granados-García M . Tumor angiogenesis as a prognostic factor in oral cavity carcinomas. J Exp Clin Cancer Res. (2001) 20:463–8., PMID: 11876537

[52] Ren J Chu T Li B Wu S Yang Z Sun G . Personalized 3D vaccine integrates immunotherapy and antiangiogenic therapy to prevent oral squamous cell carcinoma recurrence. Adv Mater. (2025) 37:e2417708. doi: 10.1002/adma.202417708, PMID: 40708335

[53] Su J Song Y Zhu Z Huang X Fan J Qiao J . Cell-cell communication: new insights and clinical implications. Signal Transduct Target Ther. (2024) 9:196. doi: 10.1038/s41392-024-01888-z, PMID: 39107318 PMC11382761

[54] Peng Z Tong Z Ren Z Ye M Hu K . Cancer-associated fibroblasts and its derived exosomes: a new perspective for reshaping the tumor microenvironment. Mol Med. (2023) 29:66. doi: 10.1186/s10020-023-00665-y, PMID: 37217855 PMC10201773

[55] Baig MS Roy A Rajpoot S Liu D Savai R Banerjee S . Tumor-derived exosomes in the regulation of macrophage polarization. Inflammation Res. (2020) 69:435–51. doi: 10.1007/s00011-020-01318-0, PMID: 32162012

[56] Mortezaee K . Exosomes in bridging macrophage-fibroblast polarity and cancer stemness. Med Oncol. (2025) 42:216. doi: 10.1007/s12032-025-02774-6, PMID: 40397051

[57] Soler MF Abaurrea A Azcoaga P Araujo AM Caffarel MM . New perspectives in cancer immunotherapy: targeting IL-6 cytokine family. J Immunother Cancer. (2023) 11(11):13. doi: 10.1136/jitc-2023-007530, PMID: 37945321 PMC10649711

[58] Wu L Jin Y Zhao X Tang K Zhao Y Tong L . Tumor aerobic glycolysis confers immune evasion through modulating sensitivity to T cell-mediated bystander killing via TNF-α. Cell Metab. (2023) 35:1580–96.e9. doi: 10.1016/j.cmet.2023.07.001, PMID: 37506695

[59] Lin X Kang K Chen P Zeng Z Li G Xiong W . Regulatory mechanisms of PD-1/PD-L1 in cancers. Mol Cancer. (2024) 23:108. doi: 10.1186/s12943-024-02023-w, PMID: 38762484 PMC11102195

[60] Zhang H Dai Z Wu W Wang Z Zhang N Zhang L . Regulatory mechanisms of immune checkpoints PD-L1 and CTLA-4 in cancer. J Exp Clin Cancer Res. (2021) 40:184. doi: 10.1186/s13046-021-01987-7, PMID: 34088360 PMC8178863

[61] Lee JE Kim MY . Cancer epigenetics: Past, present and future. Semin Cancer Biol. (2022) 83:4–14. doi: 10.1016/j.semcancer.2021.03.025, PMID: 33798724

[62] Nishiyama A Nakanishi M . Navigating the DNA methylation landscape of cancer. Trends Genet. (2021) 37:1012–27. doi: 10.1016/j.tig.2021.05.002, PMID: 34120771

[63] Scelfo A Barra V Abdennur N Spracklin G Busato F Salinas-Luypaert C . Tunable DNMT1 degradation reveals DNMT1/DNMT3B synergy in DNA methylation and genome organization. J Cell Biol. (2024) 223(4):1012–27. doi: 10.1083/jcb.202307026, PMID: 38376465 PMC10876481

[64] Lee AV Nestler KA Chiappinelli KB . Therapeutic targeting of DNA methylation alterations in cancer. Pharmacol Ther. (2024) 258:108640. doi: 10.1016/j.pharmthera.2024.108640, PMID: 38570075

[65] Xiao X Wang W Guo C Wu J Zhang S Shi H . Hypermethylation leads to the loss of HOXA5, resulting in JAG1 expression and NOTCH signaling contributing to kidney fibrosis. Kidney Int. (2024) 106:98–114. doi: 10.1016/j.kint.2024.02.023, PMID: 38521405 PMC12862895

[66] Guo H Vuille JA Wittner BS Lachtara EM Hou Y Lin M . DNA hypomethylation silences anti-tumor immune genes in early prostate cancer and CTCs. Cell. (2023) 186:2765–82.e28. doi: 10.1016/j.cell.2023.05.028, PMID: 37327786 PMC10436379

[67] Flausino CS Daniel FI Modolo F . DNA methylation in oral squamous cell carcinoma: from its role in carcinogenesis to potential inhibitor drugs. Crit Rev Oncol Hematol. (2021) 164:103399. doi: 10.1016/j.critrevonc.2021.103399, PMID: 34147646

[68] Zhu D Zeng S Su C Li J Xuan Y Lin Y . The interaction between DNA methylation and tumor immune microenvironment: from the laboratory to clinical applications. Clin Epigenetics. (2024) 16:24. doi: 10.1186/s13148-024-01633-x, PMID: 38331927 PMC10854038

[69] Chen Y Ma D Wang X Fang J Liu X Song J . Calnexin impairs the antitumor immunity of CD4(+) and CD8(+) T cells. Cancer Immunol Res. (2019) 7:123–35. doi: 10.1158/2326-6066.Cir-18-0124, PMID: 30401678

[70] Clausen MJ Melchers LJ Mastik MF Slagter-Menkema L Groen HJ van der Laan BF . Identification and validation of WISP1 as an epigenetic regulator of metastasis in oral squamous cell carcinoma. Genes Chromosomes Cancer. (2016) 55:45–59. doi: 10.1002/gcc.22310, PMID: 26391330

[71] Xu M Sun Z Wang D Li G Feng W Qiao C . Integrative multi-omics analysis decodes HOXC9-driven Malignant transformation and metastasis in OSCC. iScience. (2025) 28:112919. doi: 10.1016/j.isci.2025.112919, PMID: 41054515 PMC12496171

[72] Kheirandish S Eshghyar N Yazdani F Amini Shakib P Hosseini-Bereshneh A Nouri Z . Methylation assessment of two DKK2 and DKK4 genes in oral squamous cell carcinoma patients. Iran J Public Health. (2020) 49:1947–53. doi: 10.18502/ijph.v49i10.4698, PMID: 33346226 PMC7719650

[73] Liu Y Cao P Xiao L Tang N Fei W Li X . Hypomethylation-associated Sox11 upregulation promotes oncogenesis via the PI3K/AKT pathway in OLP-associated OSCC. J Cell Mol Med. (2024) 28:e18556. doi: 10.1111/jcmm.18556, PMID: 39039706 PMC11263134

[74] Zhang X Feng H Li D Liu S Amizuka N Li M . Identification of differentially expressed genes induced by aberrant methylation in oral squamous cell carcinomas using integrated bioinformatic analysis. Int J Mol Sci. (2018) 19(6):15. doi: 10.3390/ijms19061698, PMID: 29875348 PMC6032197

[75] Budania S Sur D Nangal J Pilli S Mukherjee K Biswas M . LINE-1 retrotransposon encoded ORF1p expression and promoter methylation in oral squamous cell carcinoma: a pilot study. Cancer Genet. (2020) 244:21–9. doi: 10.1016/j.cancergen.2020.01.050, PMID: 32088612

[76] Basu B Chakraborty J Chandra A Katarkar A Baldevbhai JRK Dhar Chowdhury D . Genome-wide DNA methylation profile identified a unique set of differentially methylated immune genes in oral squamous cell carcinoma patients in India. Clin Epigenetics. (2017) 9:13. doi: 10.1186/s13148-017-0314-x, PMID: 28174608 PMC5292006

[77] Calanca N Francisco ALN Bizinelli D Kuasne H Barros Filho MC Flores BCT . DNA methylation-based depiction of the immune microenvironment and immune-associated long non-coding RNAs in oral cavity squamous cell carcinomas. BioMed Pharmacother. (2023) 167:115559. doi: 10.1016/j.biopha.2023.115559, PMID: 37742611

[78] Nizar Jawad Z . Epigenetic and genetic events of oral squamous cell carcinoma: perspective on DNA methylation, silencing of tumor suppressor gene, and activating oncogenes. Cell Mol Biol (Noisy-le-grand). (2025) 71:96–104. doi: 10.14715/cmb/2025.71.9.12, PMID: 41054367

[79] Morandi L Gissi D Tarsitano A Asioli S Gabusi A Marchetti C . CpG location and methylation level are crucial factors for the early detection of oral squamous cell carcinoma in brushing samples using bisulfite sequencing of a 13-gene panel. Clin Epigenetics. (2017) 9:85. doi: 10.1186/s13148-017-0386-7, PMID: 28814981 PMC5558660

[80] Sun YW Chen KM Imamura Kawasawa Y Salzberg AC Cooper TK Caruso C . Hypomethylated Fgf3 is a potential biomarker for early detection of oral cancer in mice treated with the tobacco carcinogen dibenzo[def,p]chrysene. PLoS One. (2017) 12:e0186873. doi: 10.1371/journal.pone.0186873, PMID: 29073177 PMC5658092

[81] Sun Y Yang J Cai H Liu J Liu Y Luo J . Differential OAT methylation correlates with cell infiltration in tumor microenvironment and overall survival postradiotherapy in oral squamous cell carcinoma patient. J Oral Pathol Med. (2022) 51:611–9. doi: 10.1111/jop.13328, PMID: 35708285

[82] Xiao T Rahhal O Wang L Deng Z Wang R Xu X . TPPP3, a good prognostic indicator, suppresses cell proliferation and migration in OSCC. Int Dent J. (2025) 75:970–83. doi: 10.1016/j.identj.2024.09.035, PMID: 39814636 PMC11976587

[83] Lee EY Song JM Kim HJ Park HR . Hypomethylation of lncRNA H19 as a potential prognostic biomarker for oral squamous cell carcinoma. Arch Oral Biol. (2021) 129:105214. doi: 10.1016/j.archoralbio.2021.105214, PMID: 34333230

[84] Rajendran P Sekar R Abdallah BM Fathima Jh S Ali EM Jayaraman S . Epigenetic modulation of long noncoding RNA H19 in oral squamous cell carcinoma-A narrative review. Noncoding RNA Res. (2024) 9:602–11. doi: 10.1016/j.ncrna.2024.01.020, PMID: 38532798 PMC10963247

[85] Yang J Yan X Wang X Chang X Gao J Song G . Epigenetic targeting in oral squamous cell carcinoma: mechanisms, therapies, and translational potential. Expert Rev Anticancer Ther. (2025) 26(1):1–18. doi: 10.1080/14737140.2025.2570164, PMID: 41037064

[86] Kim SY Han YK Song JM Lee CH Kang K Yi JM . Aberrantly hypermethylated tumor suppressor genes were identified in oral squamous cell carcinoma (OSCC). Clin Epigenetics. (2019) 11:116. doi: 10.1186/s13148-019-0715-0, PMID: 31405379 PMC6689875

[87] Ren XY Chen XJ Chen XB Wang CY Liu Q Pan X . Immune landscape of the B7 and TNFR families in oral squamous cell carcinoma. Chin J Dent Res. (2020) 23:109–17. doi: 10.3290/j.cjdr.a44747, PMID: 32548602

[88] Ko HH Peng HH Chou HE Hou HH Liu WW Kuo MY . Downregulation of PAX1 in OSCC enhances stemness and immunosuppression via IFIT1 and PD-L1 pathways. Oral Dis. (2025) 31:2071–83. doi: 10.1111/odi.15225, PMID: 39748231

[89] Supic G Stefik D Ivkovic N Sami A Zeljic K Jovic S . Prognostic impact of miR-34b/c DNA methylation, gene expression, and promoter polymorphism in HPV-negative oral squamous cell carcinomas. Sci Rep. (2022) 12:1296. doi: 10.1038/s41598-022-05399-1, PMID: 35079080 PMC8789922

[90] Cortez MA Ivan C Valdecanas D Wang X Peltier HJ Ye Y . PDL1 Regulation by p53 via miR-34. J Natl Cancer Inst. (2016) 108(1):9. doi: 10.1093/jnci/djv303, PMID: 26577528 PMC4862407

[91] Chen YJ Liao SW Lai YL Li YF Lu YC Tai CK . Epigenetic downregulation of the proapoptotic gene HOXA5 in oral squamous cell carcinoma. Mol Med Rep. (2025) 31(3):10. doi: 10.3892/mmr.2024.13421, PMID: 39704209 PMC11683450

[92] Bhol CS Mishra SR Patil S Sahu SK Kirtana R Manna S . PAX9 reactivation by inhibiting DNA methyltransferase triggers antitumor effect in oral squamous cell carcinoma. Biochim Biophys Acta Mol Basis Dis. (2022) 1868:166428. doi: 10.1016/j.bbadis.2022.166428, PMID: 35533906

[93] Liao SW Liao XH Wu SH Li YF Chen PY Wang YL . Methylation-mediated silencing of miR-124–3 regulates LRRC1 expression and promotes oral cancer progression. Cancers (Basel). (2025) 17(7):17. doi: 10.3390/cancers17071136, PMID: 40227650 PMC11988110

[94] Padam KSR Basavarajappa DS Kumar NAN Gadicherla S Chakrabarty S Hunter KD . Epigenetic regulation of HOXA3 and its impact on oral squamous cell carcinoma progression. Oral Surg Oral Med Oral Pathol Oral Radiol. (2025) 139:550–63. doi: 10.1016/j.oooo.2024.11.088, PMID: 39658479

[95] Shojaeian S Moazeni-Roodi A Allameh A Garajei A Kazemnejad A Kabir K . Methylation of TGM-3 promoter and its association with oral squamous cell carcinoma (OSCC). Avicenna J Med Biotechnol. (2021) 13:65–73. doi: 10.18502/ajmb.v13i2.5523, PMID: 34012521 PMC8112137

[96] Li X Li Z Gao Q Peng Y Yu Y Hu T . Correlation of DNA methylation of DNMT3A and TET2 with oral squamous cell carcinoma. Discov Oncol. (2024) 15:15. doi: 10.1007/s12672-024-00866-9, PMID: 38246976 PMC10800327

[97] Huang W Liu H Lv T . Silencing of SETD6 inhibits the tumorigenesis of oral squamous cell carcinoma by inhibiting methylation of PAK4 and RelA. Histol Histopathol. (2021) 36:229–37. doi: 10.14670/hh-18-327, PMID: 33710605

[98] Yang H Jin X Dan H Chen Q . Histone modifications in oral squamous cell carcinoma and oral potentially Malignant disorders. Oral Dis. (2020) 26:719–32. doi: 10.1111/odi.13115, PMID: 31056829

[99] Yao W Hu X Wang X . Crossing epigenetic frontiers: the intersection of novel histone modifications and diseases. Signal Transduct Target Ther. (2024) 9:232. doi: 10.1038/s41392-024-01918-w, PMID: 39278916 PMC11403012

[100] Mao W Wang B Huang R Sun Z Yan M Dong P . Histone modifications in head and neck squamous cell carcinoma. Front Oncol. (2024) 14:1427725. doi: 10.3389/fonc.2024.1427725, PMID: 38983924 PMC11231198

[101] Veerasamy V Veeran V Nagini S . Dysregulated PI3K/AKT signaling in oral squamous cell carcinoma: The tumor microenvironment and epigenetic modifiers as key drivers. Oncol Res. (2025) 33:1835–60. doi: 10.32604/or.2025.064010, PMID: 40746882 PMC12308251

[102] Rajendran P Prasad M Ali EM Sekar R AlZahrani AM Karobari MI . Molecular insight into histone methylation as a novel target for oral squamous cell carcinoma: future hope in personalised medicine. J Cancer. (2025) 16:1575–90. doi: 10.7150/jca.103243, PMID: 39991574 PMC11843246

[103] Yang J Xu J Wang W Zhang B Yu X Shi S . Epigenetic regulation in the tumor microenvironment: molecular mechanisms and therapeutic targets. Signal Transduct Target Ther. (2023) 8:210. doi: 10.1038/s41392-023-01480-x, PMID: 37217462 PMC10203321

[104] Liu K Li Y Shen M Xu W Wu S Yang X . Epigenetic regulation of stromal and immune cells and therapeutic targets in the tumor microenvironment. Biomolecules. (2025) 15(1):25. doi: 10.3390/biom15010071, PMID: 39858465 PMC11764280

[105] Khan T Iftikhar F Akhlaq R Musharraf SG Ali A . Acidotic and hypoxic tumor microenvironment induces changes to histone acetylation and methylation in oral squamous cell carcinoma. BioMed Chromatogr. (2023) 37:e5616. doi: 10.1002/bmc.5616, PMID: 36882186

[106] Akhlaq R Ahmed T Khan T Yaseen Jeelani SU Joseph-Chowdhury JN Sidoli S . PX-12 modulates vorinostat-induced acetylation and methylation marks in CAL 27 cells. Epigenomics. (2025) 17:79–87. doi: 10.1080/17501911.2024.2441652, PMID: 39716806 PMC11792842

[107] Zhang L Hu G . Tumor promoting role of NRIP1 in oral squamous cell carcinoma: the involvement of NSD2-mediated histone methylation of DGCR8. Tohoku J Exp Med. (2023) 260:193–204. doi: 10.1620/tjem.2023.J029, PMID: 37045786

[108] Yang L Zhang Q Yang Q . KDM3A promotes oral squamous cell carcinoma cell proliferation and invasion via H3K9me2 demethylation-activated DCLK1. Genes Genomics. (2022) 44:1333–42. doi: 10.1007/s13258-022-01287-0, PMID: 36094735

[109] Tachaveeraphong W Phattarataratip E . The significance of modified histone H3 in epithelial dysplasia and oral cancer. Int Dent J. (2024) 74:769–76. doi: 10.1016/j.identj.2024.01.011, PMID: 38326164 PMC11287179

[110] Shahhosseini A Bourova-Flin E Derakhshan S Aminishakib P Goudarzi A . High levels of histone H3 K27 acetylation and tri-methylation are associated with shorter survival in oral squamous cell carcinoma patients. Biomedicine (Taipei). (2023) 13:22–38. doi: 10.37796/2211-8039.1391, PMID: 37168723 PMC10166250

[111] Lu Y Yang J Zhu J Shu Y Zou X Ruan Q . Advances in the histone acetylation modification in the oral squamous cell carcinoma. J Oncol. (2023) 2023:4616682. doi: 10.1155/2023/4616682, PMID: 39282225 PMC11401686

[112] Nunes SP Morales L Rubio C Munera-Maravilla E Lodewijk I Suárez-Cabrera C . Modulation of tumor microenvironment by targeting histone acetylation in bladder cancer. Cell Death Discov. (2024) 10:1. doi: 10.1038/s41420-023-01786-3, PMID: 38172127 PMC10764810

[113] Mahale A Routholla G Lavanya S Sharma P Ghosh B Kulkarni OP . Pharmacological blockade of HDAC6 attenuates cancer progression by inhibiting IL-1β and modulating immunosuppressive response in OSCC. Int Immunopharmacol. (2024) 132:111921. doi: 10.1016/j.intimp.2024.111921, PMID: 38547770

[114] Hatanaka Y Niinuma T Kitajima H Nishiyama K Maruyama R Ishiguro K . DLEU1 promotes oral squamous cell carcinoma progression by activating interferon-stimulated genes. Sci Rep. (2021) 11:20438. doi: 10.1038/s41598-021-99736-5, PMID: 34650128 PMC8516910

[115] Zhang J Zhu H Li L Gao Y Yu B Ma G . New mechanism of LncRNA: In addition to act as a ceRNA. Noncoding RNA Res. (2024) 9:1050–60. doi: 10.1016/j.ncrna.2024.06.002, PMID: 39022688 PMC11254507

[116] Ma H Chang H Yang W Lu Y Hu J Jin S . A novel IFNα-induced long noncoding RNA negatively regulates immunosuppression by interrupting H3K27 acetylation in head and neck squamous cell carcinoma. Mol Cancer. (2020) 19:4. doi: 10.1186/s12943-019-1123-y, PMID: 31907020 PMC6943933

[117] Nazário WR Costa A de Oliveira Santos D Del Nero NRD Rodrigues TS Borges LDF . Sex-related differences in histone acetylation and tumor development in a 4-nitroquinoline 1-oxide and ethanol-induced oral squamous cell carcinoma mouse model. J Oral Pathol Med. (2025) 54:1074–84. doi: 10.1111/jop.70062, PMID: 40928052 PMC12602132

[118] Yuan M Wang K Pang B Lu H Jiang S Song K . Preliminary investigation of the epigenetic regulation of KMT2D via H3K4me1/H3K27ac in oral squamous cell carcinoma. Cell Mol Life Sci. (2025) 82:237. doi: 10.1007/s00018-025-05765-y, PMID: 40515851 PMC12167212

[119] Xiao Y Zhang Y Hu Y Zhang X Tan J Yao S . Advances in the study of posttranslational modifications of histones in head and neck squamous cell carcinoma. Clin Epigenetics. (2024) 16:165. doi: 10.1186/s13148-024-01785-w, PMID: 39574168 PMC11580233

[120] Zhang L Xu X Su X . Noncoding RNAs in cancer immunity: functions, regulatory mechanisms, and clinical application. Mol Cancer. (2020) 19:48. doi: 10.1186/s12943-020-01154-0, PMID: 32122338 PMC7050126

[121] Balakittnen J Weeramange CE Wallace DF Duijf PHG Cristino AS Kenny L . Noncoding RNAs in oral cancer. Wiley Interdiscip Rev RNA. (2023) 14:e1754. doi: 10.1002/wrna.1754, PMID: 35959932 PMC10909450

[122] Huang F Xin C Lei K Bai H Li J Chen Q . Noncoding RNAs in oral premalignant disorders and oral squamous cell carcinoma. Cell Oncol (Dordr). (2020) 43:763–77. doi: 10.1007/s13402-020-00521-9, PMID: 32495292 PMC12990699

[123] He Z Ji Y Yuan Y Liang T Liu C Jiao Y . Uncovering the role of microRNAs in esophageal cancer: from pathogenesis to clinical applications. Front Pharmacol. (2025) 16:1532558. doi: 10.3389/fphar.2025.1532558, PMID: 39944625 PMC11814179

[124] Xu Z Chen Y Ma L Chen Y Liu J Guo Y . Role of exosomal non-coding RNAs from tumor cells and tumor-associated macrophages in the tumor microenvironment. Mol Ther. (2022) 30:3133–54. doi: 10.1016/j.ymthe.2022.01.046, PMID: 35405312 PMC9552915

[125] Rinn JL Chang HY . Long noncoding RNAs: molecular modalities to organismal functions. Annu Rev Biochem. (2020) 89:283–308. doi: 10.1146/annurev-biochem-062917-012708, PMID: 32569523

[126] Guo CJ Xu G Chen LL . Mechanisms of long noncoding RNA nuclear retention. Trends Biochem Sci. (2020) 45:947–60. doi: 10.1016/j.tibs.2020.07.001, PMID: 32800670

[127] Xu Y Jiang E Shao Z Shang Z . Long noncoding RNAs in the metastasis of oral squamous cell carcinoma. Front Oncol. (2020) 10:616717. doi: 10.3389/fonc.2020.616717, PMID: 33520725 PMC7845733

[128] Li X Ren Y Hao H Jin Y Chen B Zhao K . Long non-coding RNAs as key orchestrators of the tumor microenvironment in lung cancer. Front Immunol. (2025) 16:1716180. doi: 10.3389/fimmu.2025.1716180, PMID: 41409273 PMC12705557

[129] Chen Y Li Z Chen X Zhang S . Long non-coding RNAs: From disease code to drug role. Acta Pharm Sin B. (2021) 11:340–54. doi: 10.1016/j.apsb.2020.10.001, PMID: 33643816 PMC7893121

[130] Zhang S Wang X Wang D . Long non-coding RNA LINC01296 promotes progression of oral squamous cell carcinoma through activating the MAPK/ERK signaling pathway via the miR-485-5p/PAK4 axis. Arch Med Sci. (2022) 18:786–99. doi: 10.5114/aoms.2019.86805, PMID: 35591837 PMC9102572

[131] Che H Che Y Zhang Z Lu Q . Long non-coding RNA LINC01929 accelerates progression of oral squamous cell carcinoma by targeting the miR-137-3p/FOXC1 axis. Front Oncol. (2021) 11:657876. doi: 10.3389/fonc.2021.657876, PMID: 33968763 PMC8097103

[132] Tong F Guo J Miao Z Li Z . LncRNA SNHG17 promotes the progression of oral squamous cell carcinoma by modulating miR-375/PAX6 axis. Cancer biomark. (2021) 30:1–12. doi: 10.3233/cbm-191070, PMID: 32924983 PMC12499959

[133] Guo S Huang B You Z Luo Z Xu D Zhang J . FOXD2-AS1 promotes Malignant cell behavior in oral squamous cell carcinoma via the miR-378 g/CRABP2 axis. BMC Oral Health. (2024) 24:625. doi: 10.1186/s12903-024-04388-2, PMID: 38807101 PMC11134640

[134] Zhang Y Mao Q Xia Q Cheng J Huang Z Li Y . Noncoding RNAs link metabolic reprogramming to immune microenvironment in cancers. J Hematol Oncol. (2021) 14:169. doi: 10.1186/s13045-021-01179-y, PMID: 34654454 PMC8518176

[135] Wei X Li Z Zheng H Li X Lin Y Yang H . Long non-coding RNA MAGEA4-AS1 binding to p53 enhances MK2 signaling pathway and promotes the proliferation and metastasis of oral squamous cell carcinoma. Funct Integr Genomics. (2024) 24:158. doi: 10.1007/s10142-024-01436-6, PMID: 39249547 PMC11384635

[136] Zhou W Feng Y Lin C Chao CK He Z Zhao S . Yin yang 1-induced long noncoding RNA DUXAP9 drives the progression of oral squamous cell carcinoma by blocking CDK1-mediated EZH2 degradation. Adv Sci (Weinh). (2023) 10:e2207549. doi: 10.1002/advs.202207549, PMID: 37401236 PMC10477890

[137] Jiang X Liang X Li S Yang Y Xu X Gu W . The LINC00319 binding to STAT3 promotes the cell proliferation, migration, invasion and EMT process in oral squamous cell carcinoma. Arch Biochem Biophys. (2024) 761:110170. doi: 10.1016/j.abb.2024.110170, PMID: 39366629

[138] Jiao J Zhao Y Li Q Jin S Liu Z . LncRNAs in tumor metabolic reprogramming and tumor microenvironment remodeling. Front Immunol. (2024) 15:1467151. doi: 10.3389/fimmu.2024.1467151, PMID: 39539540 PMC11557318

[139] Nakashima C Fujiwara-Tani R Mori S Kishi S Ohmori H Fujii K . An axis between the long non-coding RNA HOXA11-AS and NQOs enhances metastatic ability in oral squamous cell carcinoma. Int J Mol Sci. (2022) 23(18):17. doi: 10.3390/ijms231810704, PMID: 36142607 PMC9506332

[140] Zhu W Wang J Liu X Xu Y Zhai R Zhang J . lncRNA CYTOR promotes aberrant glycolysis and mitochondrial respiration via HNRNPC-mediated ZEB1 stabilization in oral squamous cell carcinoma. Cell Death Dis. (2022) 13:703. doi: 10.1038/s41419-022-05157-1, PMID: 35963855 PMC9376070

[141] Li W Zhang H You Z Guo B . LncRNAs in immune and stromal cells remodel phenotype of cancer cell and tumor microenvironment. J Inflammation Res. (2024) 17:3173–85. doi: 10.2147/jir.S460730, PMID: 38774447 PMC11108079

[142] Li Y Ma Z Li W Xu X Shen P Zhang SE . PDPN(+) CAFs facilitate the motility of OSCC cells by inhibiting ferroptosis via transferring exosomal lncRNA FTX. Cell Death Dis. (2023) 14:759. doi: 10.1038/s41419-023-06280-3, PMID: 37993428 PMC10665425

[143] Zhang D Song Y Li D Liu X Pan Y Ding L . Cancer-associated fibroblasts promote tumor progression by lncRNA-mediated RUNX2/GDF10 signaling in oral squamous cell carcinoma. Mol Oncol. (2022) 16:780–94. doi: 10.1002/1878-0261.12935, PMID: 33657265 PMC8807363

[144] Ai Y Liu S Luo H Wu S Wei H Tang Z . lncRNA DCST1-AS1 facilitates oral squamous cell carcinoma by promoting M2 macrophage polarization through activating NF-κB signaling. J Immunol Res. (2021) 2021:5524231. doi: 10.1155/2021/5524231, PMID: 34414241 PMC8369177

[145] Zou C Wu S Wei H Luo H Tang Z Li X . LINC01355 contributes to Malignant phenotype of oral squamous cell carcinoma and cytotoxic T cell infiltration via activating notch signaling pathway. J Immunol Res. (2021) 2021:1830790. doi: 10.1155/2021/1830790, PMID: 34355042 PMC8331309

[146] Zou C Lv X Wei H Wu S Song J Tang Z . Long non-coding RNA LINC00472 inhibits oral squamous cell carcinoma via miR-4311/GNG7 axis. Bioengineered. (2022) 13:6371–82. doi: 10.1080/21655979.2022.2040768, PMID: 35240924 PMC8974029

[147] Gao YQ Shi PW Shi WK Liu YM . Expression and mechanism of long non-coding RNA HCG22 in oral squamous cell carcinoma. Hua Xi Kou Qiang Yi Xue Za Zhi. (2021) 39:658–66. doi: 10.7518/hxkq.2021.06.006, PMID: 34859625 PMC8703100

[148] Du Y Shuai Y Wang H Li H Li Y . Exosome-mediated long noncoding RNA (lncRNA) PART1 suppresses Malignant progression of oral squamous cell carcinoma via miR-17-5p/SOCS6 axis. Turk J Med Sci. (2023) 53:630–9. doi: 10.55730/1300-0144.5625, PMID: 37476905 PMC10388088

[149] Shafiee Allaf O Li W Zeng C Li P Zhang Y Xu Y . LncRNA PCBP1-AS1 suppresses cell growth in oral squamous cell carcinoma by targeting miR-34c-5p/ZFP36 axis. Cell Mol Biol (Noisy-le-grand). (2024) 70:99–105. doi: 10.14715/cmb/2024.70.9.14, PMID: 39380272

[150] Hu Y Lv F Li N Yuan X Zhang L Zhao S . Long noncoding RNA MEG3 inhibits oral squamous cell carcinoma progression via GATA3. FEBS Open Bio. (2023) 13:195–208. doi: 10.1002/2211-5463.13532, PMID: 36468944 PMC9811608

[151] Zhuang Z Huang J Wang W Wang C Yu P Hu J . Down-regulation of long non-coding RNA TINCR induces cell dedifferentiation and predicts progression in oral squamous cell carcinoma. Front Oncol. (2020) 10:624752. doi: 10.3389/fonc.2020.624752, PMID: 33732637 PMC7959775

[152] Dong C Zhao Y Yang S Jiao X . LINC00173 blocks GATA6-mediated transcription of COL5A1 to affect Malignant development of oral squamous cell carcinoma. J Oral Pathol Med. (2023) 52:493–503. doi: 10.1111/jop.13425, PMID: 36856154

[153] Li Y Lin M Wang S Cao B Li C Li G . Novel angiogenic regulators and anti-angiogenesis drugs targeting angiogenesis signaling pathways: perspectives for targeting angiogenesis in lung cancer. Front Oncol. (2022) 12:842960. doi: 10.3389/fonc.2022.842960, PMID: 35372042 PMC8965887

[154] Huang CY Chou ST Hsu YM Chao WJ Wu GH Hsiao JR . MEG3-mediated oral squamous-cell-carcinoma-derived exosomal miR-421 activates angiogenesis by targeting HS2ST1 in vascular endothelial cells. Int J Mol Sci. (2024) 25(14):19. doi: 10.3390/ijms25147576, PMID: 39062818 PMC11277508

[155] Qin SY Li B Liu JM Lv QL Zeng XL . LncRNA NR2F2-AS1 inhibits the progression of oral squamous cell carcinoma by mediating the miR-32-5p/SEMA3A axis. Kaohsiung J Med Sci. (2024) 40:877–89. doi: 10.1002/kjm2.12888, PMID: 39177014 PMC11895586

[156] Ai Y Wei H Wu S Tang Z Li X Zou C . Exosomal LncRNA LBX1-AS1 Derived From RBPJ Overexpressed-Macrophages Inhibits Oral Squamous Cell Carcinoma Progress via miR-182-5p/FOXO3. Front Oncol. (2021) 11:605884. doi: 10.3389/fonc.2021.605884, PMID: 33816238 PMC8010199

[157] Sun Y Pan J Li Y Hu Y Ma J Chen F . Restoring BARX2 in OSCC reverses partial EMT and suppresses metastasis through miR-186-5p/miR-378a-3p-dependent SERPINE2 inhibition. Oncogene. (2024) 43:1941–54. doi: 10.1038/s41388-024-03053-w, PMID: 38719950

[158] Malekjafarian SM Mohtasham N Mirhashemi M Sadeghi M Arab F Mohajertehran F . Metastasis and cell proliferation inhibition by microRNAs and its potential therapeutic applications in OSCC: A systematic review. Pathol Res Pract. (2024) 262:155532. doi: 10.1016/j.prp.2024.155532, PMID: 39142242

[159] Guo Y Qiao X Zhu L Song R . MicroRNA-182-5p modulates oral squamous cell carcinoma migration and invasion via targeting MTSS1 gene. Pathol Oncol Res. (2020) 26:1007–13. doi: 10.1007/s12253-019-00647-8, PMID: 30949866

[160] Li K Zhou Z Li J Xiang R . miR-146b functions as an oncogene in oral squamous cell carcinoma by targeting HBP1. Technol Cancer Res Treat. (2020) 19:1533033820959404. doi: 10.1177/1533033820959404, PMID: 33327874 PMC7750896

[161] Zheng TL Cen K . MiR-92a inhibits proliferation and promotes apoptosis of OSCC cells through Wnt/β-catenin signaling pathway. Eur Rev Med Pharmacol Sci. (2020) 24:4803–9. doi: 10.26355/eurrev_202005_21169, PMID: 32432743

[162] Wang L Song Y Wang H Liu K Shao Z Shang Z . MiR-210-3p-EphrinA3-PI3K/AKT axis regulates the progression of oral cancer. J Cell Mol Med. (2020) 24:4011–22. doi: 10.1111/jcmm.15036, PMID: 32180353 PMC7171305

[163] Peng M Pang C . MicroRNA-140-5p inhibits the tumorigenesis of oral squamous cell carcinoma by targeting p21-activated kinase 4. Cell Biol Int. (2020) 44:145–54. doi: 10.1002/cbin.11213, PMID: 31393040

[164] Wang X Chang K Gao J Wei J Xu G Xiao L . MicroRNA-504 functions as a tumor suppressor in oral squamous cell carcinoma through inhibiting cell proliferation, migration and invasion by targeting CDK6. Int J Biochem Cell Biol. (2020) 119:105663. doi: 10.1016/j.biocel.2019.105663, PMID: 31812760

[165] Zhou YM Yao YL Liu W Shen XM Shi LJ Wu L . MicroRNA-134 inhibits tumor stem cell migration and invasion in oral squamous cell carcinomas via downregulation of PI3K-Akt signaling pathway by inhibiting LAMC2 expression. Cancer biomark. (2020) 29:51–67. doi: 10.3233/cbm-191362, PMID: 32568182 PMC12662496

[166] He J Ye W Kou N Chen K Cui B Zhang X . MicroRNA-29b-3p suppresses oral squamous cell carcinoma cell migration and invasion via IL32/AKT signalling pathway. J Cell Mol Med. (2020) 24:841–9. doi: 10.1111/jcmm.14794, PMID: 31680452 PMC6933408

[167] Liu H Zheng J Ren Z Shen K Zeng Y . miR-107 modulates EMT progression of OSCC by targeting SNCG and inhibiting the ERK/NF-κB signaling pathways. J Transl Med. (2025) 23:881. doi: 10.1186/s12967-025-06910-8, PMID: 40775646 PMC12330075

[168] Ou D Wu Y Zhang J Liu J Liu Z Shao M . miR-340-5p affects oral squamous cell carcinoma (OSCC) cells proliferation and invasion by targeting endoplasmic reticulum stress proteins. Eur J Pharmacol. (2022) 920:174820. doi: 10.1016/j.ejphar.2022.174820, PMID: 35227681

[169] Cui Z Liu QL Sun SQ Jiao K Liu DR Zhou XC . MiR-378a-5p inhibits angiogenesis of oral squamous cell carcinoma by targeting KLK4. Neoplasma. (2020) 67:85–92. doi: 10.4149/neo_2019_190306N191, PMID: 31829025

[170] Giarratana AO Prendergast CM Salvatore MM Capaccione KM . TGF-β signaling: critical nexus of fibrogenesis and cancer. J Transl Med. (2024) 22:594. doi: 10.1186/s12967-024-05411-4, PMID: 38926762 PMC11201862

[171] You X Zhou Z Chen W Wei X Zhou H Luo W . MicroRNA-495 confers inhibitory effects on cancer stem cells in oral squamous cell carcinoma through the HOXC6-mediated TGF-β signaling pathway. Stem Cell Res Ther. (2020) 11:117. doi: 10.1186/s13287-020-1576-3, PMID: 32171324 PMC7071696

[172] Wang Y Jia RZ Diao S He J Jia L . miRNA-101 targets TGF-βR1 to retard the progression of oral squamous cell carcinoma. Oncol Res. (2020) 28:203–12. doi: 10.3727/096504019x15761480623959, PMID: 31831099 PMC7851522

[173] Tao M Zheng M Xu Y Ma S Zhang W Ju S . CircRNAs and their regulatory roles in cancers. Mol Med. (2021) 27:94. doi: 10.1186/s10020-021-00359-3, PMID: 34445958 PMC8393742

[174] Zhang W Xu C Yang Z Zhou J Peng W Zhang X . Circular RNAs in tumor immunity and immunotherapy. Mol Cancer. (2024) 23:171. doi: 10.1186/s12943-024-02082-z, PMID: 39169354 PMC11337656

[175] Zhu M Chen D Ruan C Yang P Zhu J Zhang R . CircRNAs: A promising star for treatment and prognosis in oral squamous cell carcinoma. Int J Mol Sci. (2023) 24(18):20. doi: 10.3390/ijms241814194, PMID: 37762497 PMC10532269

[176] Zhou J Jin S . Circ_0058063 contributed to oral squamous cell carcinoma development by sponging miR-145 and regulating PI3K/AKT pathway. Mol Biotechnol. (2023) 65:2049–60. doi: 10.1007/s12033-023-00715-0, PMID: 36928742

[177] Huang C Li H Zhou L Li D . Circ_0005050 promotes the proliferation of oral squamous cell carcinoma and inhibits the apoptosis by activating JAK/STAT3 signaling pathway. Pathol Res Pract. (2022) 238:154058. doi: 10.1016/j.prp.2022.154058, PMID: 36155326

[178] Li L Yin Y Nan F Ma Z . Circ_LPAR3 promotes the progression of oral squamous cell carcinoma (OSCC). Biochem Biophys Res Commun. (2022) 589:215–22. doi: 10.1016/j.bbrc.2021.12.012, PMID: 34922206

[179] Li X Wang C Zhang H Li Y Hou D Liu D . circFNDC3B accelerates vasculature formation and metastasis in oral squamous cell carcinoma. Cancer Res. (2023) 83:1459–75. doi: 10.1158/0008-5472.Can-22-2585, PMID: 36811957 PMC10152237

[180] Long Y Li C Zhu B . Circ_0008068 facilitates the oral squamous cell carcinoma development by microRNA-153-3p/acylgycerol kinase (AGK) axis. Bioengineered. (2022) 13:13055–69. doi: 10.1080/21655979.2022.2074106, PMID: 35635053 PMC9275858

[181] Hei N Liu P Jin L Peng S Bao Y . Circular hsa_circ_0020377 regulates KLF7 by targeting miR-194-5p to facilitate tumor cell Malignant behaviors and glycolysis in oral squamous cell carcinoma progression. Funct Integr Genomics. (2023) 23:52. doi: 10.1007/s10142-023-00973-w, PMID: 36717528

[182] Su Z Pan C Xie H Ning Y Li S Xiao H . Downregulation of circLPAR3 inhibits tumor progression and glycolysis by liberating miR-144-3p and upregulating LPCAT1 in oral squamous cell carcinoma. Laryngoscope Investig Otolaryngol. (2022) 7:425–36. doi: 10.1002/lio2.771, PMID: 35434335 PMC9008151

[183] Zhang H Wang Z Zhang Z . Hsa_circ_0009128 mediates progression of oral squamous cell carcinoma by influencing MMP9. Oral Dis. (2023) 29:661–71. doi: 10.1111/odi.14019, PMID: 34514700

[184] Li B Xie S Pan G Lin Z Ren S Chen S . Circ-OMAC drives metastasis in oral squamous cell carcinoma. Oral Dis. (2024) 30:140–8. doi: 10.1111/odi.14387, PMID: 36135340

[185] Zhang Q Jiang C Ren W Li S Zheng J Gao Y . Circ-LRP6 mediates epithelial-mesenchymal transition and autophagy in oral squamous cell carcinomas. J Oral Pathol Med. (2021) 50:660–7. doi: 10.1111/jop.13163, PMID: 33501755

[186] Lv JL Ma R Ren YS Liang QY Zhang HM Dong GC . CircRNA: the potential biomarkers and therapeutic targets in oral squamous cell carcinoma (OSCC). Front Oncol. (2025) 15:1555002. doi: 10.3389/fonc.2025.1555002, PMID: 40538852 PMC12176845

[187] Yuan G Wei L Zheng X Xiong J Liu H . Circ_0049396 attenuates the progression of oral squamous cell carcinoma by miR-650 suppression to induce neurofilament heavy polypeptide enhancement. Oral Surg Oral Med Oral Pathol Oral Radiol. (2023) 136(6):703–13. doi: 10.1016/j.oooo.2023.07.047, PMID: 39492301

[188] Dong W Zhao L Zhang S Zhang S Si H . Circ-KIAA0907 inhibits the progression of oral squamous cell carcinoma by regulating the miR-96-5p/UNC13C axis. World J Surg Oncol. (2021) 19:75. doi: 10.1186/s12957-021-02184-8, PMID: 33715625 PMC7962272

[189] Li B Ding Y Huo C Ren S Chen S He H . Targeting Circ-OCAC suppress oral squamous cell carcinoma progression. Oral Dis. (2024) 30:2202–18. doi: 10.1111/odi.14687, PMID: 37485985

[190] Xia Y Hei N Peng S Cui Z . The role and mechanism of circ-BNC2 on the Malignant progression of oral squamous cell carcinoma. Head Neck. (2023) 45:2424–37. doi: 10.1002/hed.27442, PMID: 37377048

[191] Han L Cheng J Li A . hsa_circ_0072387 suppresses proliferation, metastasis, and glycolysis of oral squamous cell carcinoma cells by downregulating miR-503-5p. Cancer Biother Radiopharm. (2021) 36:84–94. doi: 10.1089/cbr.2019.3371, PMID: 32302508

[192] Dai Y Zhu Y Xu H . circ_0004872 inhibits proliferation, invasion, and glycolysis of oral squamous cell carcinoma by sponged miR-424-5p. J Clin Lab Anal. (2022) 36:e24486. doi: 10.1002/jcla.24486, PMID: 35576499 PMC9280002

[193] Hogg SJ Beavis PA Dawson MA Johnstone RW . Targeting the epigenetic regulation of antitumour immunity. Nat Rev Drug Discov. (2020) 19:776–800. doi: 10.1038/s41573-020-0077-5, PMID: 32929243

[194] Ramaiah MJ Tangutur AD Manyam RR . Epigenetic modulation and understanding of HDAC inhibitors in cancer therapy. Life Sci. (2021) 277:119504. doi: 10.1016/j.lfs.2021.119504, PMID: 33872660

[195] Peng Q Yang JY Zhou G . Emerging functions and clinical applications of exosomes in human oral diseases. Cell Biosci. (2020) 10:68. doi: 10.1186/s13578-020-00424-0, PMID: 32489584 PMC7245751

[196] Morel D Jeffery D Aspeslagh S Almouzni G Postel-Vinay S . Combining epigenetic drugs with other therapies for solid tumours - past lessons and future promise. Nat Rev Clin Oncol. (2020) 17:91–107. doi: 10.1038/s41571-019-0267-4, PMID: 31570827

[197] Xie Z Zhou Z Yang S Zhang S Shao B . Epigenetic regulation and therapeutic targets in the tumor microenvironment. Mol Biomed. (2023) 4:17. doi: 10.1186/s43556-023-00126-2, PMID: 37273004 PMC10241773

[198] Takahashi S Yoshida K Paudel D Morikawa T Uehara O Harada F . Epigenetic agents, zebularine and valproic acid, inhibit the growth of the oral squamous cell carcinoma cell line HSC4 *in vitro* and *in vivo*. Discov Oncol. (2025) 16:1293. doi: 10.1007/s12672-025-02928-y, PMID: 40632344 PMC12241533

[199] More DA Singh N Mishra R Muralidharan HP Gopinath KS Gopal C . Intronic miR-6741-3p targets the oncogene SRSF3: Implications for oral squamous cell carcinoma pathogenesis. PLoS One. (2024) 19:e0296565. doi: 10.1371/journal.pone.0296565, PMID: 38781195 PMC11115324

[200] Ushio R Hiroi M Matsumoto A Mori K Yamamoto N Ohmori Y . Enhanced cytotoxic effects in human oral squamous cell carcinoma cells treated with combined methyltransferase inhibitors and histone deacetylase inhibitors. Biomedicines. (2022) 10(4):17. doi: 10.3390/biomedicines10040763, PMID: 35453513 PMC9029187

[201] Ranjbar A Shahabi S Alaeddini M Mohammadpour H Hodjat M Saberi S . Synergistic effects of 5-azacitidine and photodynamic therapy using spirulina platensis on cell death in CAL27 oral squamous cell carcinoma Cells: Synergistic Anticancer Effects of 5-Azacitidine and Spirulina-Based Photodynamic Therapy in Oral Squamous Cell Carcinoma Cells. Photodiagnosis Photodyn Ther. (2025) 56:105232. doi: 10.1016/j.pdpdt.2025.105232, PMID: 41005692

[202] Yang SC Wang WY Zhou JJ Wu L Zhang MJ Yang QC . Inhibition of DNMT1 potentiates antitumor immunity in oral squamous cell carcinoma. Int Immunopharmacol. (2022) 111:109113. doi: 10.1016/j.intimp.2022.109113, PMID: 35944462

[203] Cui Z Sun S Li J Li J Sha T He J . Inhibitor of growth 4 (ING4) plays a tumor-repressing role in oral squamous cell carcinoma via nuclear factor kappa-B (NF-kB)/DNA methyltransferase 1 (DNMT1) axis-mediated regulation of aldehyde dehydrogenase 1A2 (ALDH1A2). Curr Cancer Drug Targets. (2022) 22:771–83. doi: 10.2174/1568009622666220406104732, PMID: 35388759

[204] Tasoulas J Giaginis C Patsouris E Manolis E Theocharis S . Histone deacetylase inhibitors in oral squamous cell carcinoma treatment. Expert Opin Investig Drugs. (2015) 24:69–78. doi: 10.1517/13543784.2014.952368, PMID: 25216628

[205] Ahn MY . HDAC inhibitor apicidin suppresses murine oral squamous cell carcinoma cell growth *in vitro* and *in vivo* via inhibiting HDAC8 expression. Oncol Lett. (2018) 16:6552–60. doi: 10.3892/ol.2018.9468, PMID: 30405794 PMC6202526

[206] Chen HL Lo YH Lin CL Lee TH Leung W Wang SW . Trichodermin inhibits the growth of oral cancer through apoptosis-induced mitochondrial dysfunction and HDAC-2-mediated signaling. BioMed Pharmacother. (2022) 153:113351. doi: 10.1016/j.biopha.2022.113351, PMID: 35785707

[207] Qian K Sun L Zhou G Ge H Meng Y Li J . Sodium phenylbutyrate inhibits tumor growth and the epithelial-mesenchymal transition of oral squamous cell carcinoma *in vitro* and *in vivo*. Cancer Biother Radiopharm. (2018) 33:139–45. doi: 10.1089/cbr.2017.2418, PMID: 29658787

[208] Tavares MO Milan TM Bighetti-Trevisan RL Leopoldino AM de Almeida LO . Pharmacological inhibition of HDAC6 overcomes cisplatin chemoresistance by targeting cancer stem cells in oral squamous cell carcinoma. J Oral Pathol Med. (2022) 51:529–37. doi: 10.1111/jop.13326, PMID: 35678235

[209] Mahale A Regula S Chanchlani B Ghosh B Kulkarni OP . HDAC6 inhibition attenuates RIPK1/RIPK3/MLKL signalling and improves anti-tumor immune response in oral cancer. Int Immunopharmacol. (2025) 167:115634. doi: 10.1016/j.intimp.2025.115634, PMID: 41110172

[210] Zhao B Huang Z Qin Z Li Y Wang T Wang L . Enhancement of histone deacetylase inhibitor sensitivity in combination with cyclin-dependent kinase inhibition for the treatment of oral squamous cell carcinoma. Cell Physiol Biochem. (2019) 53:141–56. doi: 10.33594/000000126, PMID: 31237760

[211] Akhlaq R Khan T Ahmed T Musharraf SG Ali A . PX-12 synergistically enhances the therapeutic efficacy of vorinostat under hypoxic tumor microenvironment in oral squamous cell carcinoma *in vitro*. Drug Dev Res. (2023) 84:556–60. doi: 10.1002/ddr.22045, PMID: 36808757

[212] Bayat S Shekari Khaniani M Choupani J Alivand MR Mansoori Derakhshan S . HDACis (class I), cancer stem cell, and phytochemicals: Cancer therapy and prevention implications. BioMed Pharmacother. (2018) 97:1445–53. doi: 10.1016/j.biopha.2017.11.065, PMID: 29156535

[213] Silva LC Leite AA Borgato GB Wagner VP Martins MD Loureiro FJA . Oral squamous cell carcinoma cancer stem cells have different drug sensitive to pharmacological NFκB and histone deacetylation inhibition. Am J Cancer Res. (2023) 13:6038–50., PMID: 38187064 PMC10767341

[214] Sang Z Sun Y Ruan H Cheng Y Ding X Yu Y . Anticancer effects of valproic acid on oral squamous cell carcinoma via SUMOylation *in vivo* and *in vitro*. Exp Ther Med. (2016) 12:3979–87. doi: 10.3892/etm.2016.3907, PMID: 28101176 PMC5228083

[215] Zhang X Zhang H Gu J Zhang J Shi H Qian H . Engineered extracellular vesicles for cancer therapy. Adv Mater. (2021) 33:e2005709. doi: 10.1002/adma.202005709, PMID: 33644908

[216] Liu T Sun L Ji Y Zhu W . Extracellular vesicles in cancer therapy: Roles, potential application, and challenges. Biochim Biophys Acta Rev Cancer. (2024) 1879:189101. doi: 10.1016/j.bbcan.2024.189101, PMID: 38608963

[217] Zhao Z Li D Wu Z Wang Q Ma Z Zhang C . Research progress and prospect of nanoplatforms for treatment of oral cancer. Front Pharmacol. (2020) 11:616101. doi: 10.3389/fphar.2020.616101, PMID: 33391000 PMC7773899

[218] Yap T Pruthi N Seers C Belobrov S McCullough M Celentano A . Extracellular vesicles in oral squamous cell carcinoma and oral potentially Malignant disorders: A systematic review. Int J Mol Sci. (2020) 21(4):29. doi: 10.3390/ijms21041197, PMID: 32054041 PMC7072764

[219] Liu Q Jiang D Zhang S Ru Y Li J Guo P . Light-activated photosensitizer/quercetin co-loaded extracellular vesicles for precise oral squamous cell carcinoma therapy. Int J Pharm. (2025) 671:125224. doi: 10.1016/j.ijpharm.2025.125224, PMID: 39824264

[220] Li M Yin S Xu A Kang L Ma Z Liu F . Synergistic phototherapy-molecular targeted therapy combined with tumor exosome nanoparticles for oral squamous cell carcinoma treatment. Pharmaceutics. (2023) 16(1):21. doi: 10.3390/pharmaceutics16010033, PMID: 38258044 PMC10821490

[221] Wang D Wang Y Zhang S Yang X Yang Y Han T . Tetrahedral-DNA-nanostructure-modified engineered extracellular vesicles enhance oral squamous cell carcinomas therapy by targeting GPX4. ACS Nano. (2025) 19:9351–66. doi: 10.1021/acsnano.5c00674, PMID: 40014396

[222] Qiu Y Sun J Qiu J Chen G Wang X Mu Y . Antitumor activity of cabazitaxel and MSC-TRAIL derived extracellular vesicles in drug-resistant oral squamous cell carcinoma. Cancer Manag Res. (2020) 12:10809–20. doi: 10.2147/cmar.S277324, PMID: 33149686 PMC7605918

[223] Kase Y Uzawa K Wagai S Yoshimura S Yamamoto JI Toeda Y . Engineered exosomes delivering specific tumor-suppressive RNAi attenuate oral cancer progression. Sci Rep. (2021) 11:5897. doi: 10.1038/s41598-021-85242-1, PMID: 33723306 PMC7960743

[224] Sayyed AA Gondaliya P Mali M Pawar A Bhat P Khairnar A . MiR-155 inhibitor-laden exosomes reverse resistance to cisplatin in a 3D tumor spheroid and xenograft model of oral cancer. Mol Pharm. (2021) 18:3010–25. doi: 10.1021/acs.molpharmaceut.1c00213, PMID: 34176265

[225] Li L Lu S Liang X Cao B Wang S Jiang J . γδTDEs: an efficient delivery system for miR-138 with anti-tumoral and immunostimulatory roles on oral squamous cell carcinoma. Mol Ther Nucleic Acids. (2019) 14:101–13. doi: 10.1016/j.omtn.2018.11.009, PMID: 30594069 PMC6307324

[226] Toh SY Leong HS Chong FT Rodrigues-Junior DM Ren MJ Kwang XL . Therapeutic application of extracellular vesicular EGFR isoform D as a co-drug to target squamous cell cancers with tyrosine kinase inhibitors. Dev Cell. (2024) 59:2189–202.e8. doi: 10.1016/j.devcel.2024.07.003, PMID: 39089249

[227] Rosenberger L Ezquer M Lillo-Vera F Pedraza PL Ortúzar MI González PL . Stem cell exosomes inhibit angiogenesis and tumor growth of oral squamous cell carcinoma. Sci Rep. (2019) 9:663. doi: 10.1038/s41598-018-36855-6, PMID: 30679544 PMC6345809

[228] Liu P Zhang Q Mi J Wang S Xu Q Zhuang D . Exosomes derived from stem cells of human deciduous exfoliated teeth inhibit angiogenesis *in vivo* and *in vitro* via the transfer of miR-100-5p and miR-1246. Stem Cell Res Ther. (2022) 13:89. doi: 10.1186/s13287-022-02764-9, PMID: 35241153 PMC8895508

[229] Mukerjee N Bhattacharya A Maitra S Kaur M Ganesan S Mishra S . Exosome isolation and characterization for advanced diagnostic and therapeutic applications. Mater Today Bio. (2025) 31:101613. doi: 10.1016/j.mtbio.2025.101613, PMID: 40161926 PMC11950786

[230] René CA Parks RJ . Bioengineering extracellular vesicle cargo for optimal therapeutic efficiency. Mol Ther Methods Clin Dev. (2024) 32:101259. doi: 10.1016/j.omtm.2024.101259, PMID: 38770107 PMC11103572

[231] Tang TT Wang B Lv LL Liu BC . Extracellular vesicle-based Nanotherapeutics: Emerging frontiers in anti-inflammatory therapy. Theranostics. (2020) 10:8111–29. doi: 10.7150/thno.47865, PMID: 32724461 PMC7381724

[232] Jangholi A Müller Bark J Kenny L Vasani S Rao S Dolcetti R . Exosomes at the crossroad between therapeutic targets and therapy resistance in head and neck squamous cell carcinoma. Biochim Biophys Acta Rev Cancer. (2022) 1877:188784. doi: 10.1016/j.bbcan.2022.188784, PMID: 36028150

[233] Nussinov R Tsai CJ Jang H . Anticancer drug resistance: An update and perspective. Drug Resist Updat. (2021) 59:100796. doi: 10.1016/j.drup.2021.100796, PMID: 34953682 PMC8810687

[234] Pu Y Li L Peng H Liu L Heymann D Robert C . Drug-tolerant persister cells in cancer: the cutting edges and future directions. Nat Rev Clin Oncol. (2023) 20:799–813. doi: 10.1038/s41571-023-00815-5, PMID: 37749382

[235] Mikubo M Inoue Y Liu G Tsao MS . Mechanism of drug tolerant persister cancer cells: the landscape and clinical implication for therapy. J Thorac Oncol. (2021) 16:1798–809. doi: 10.1016/j.jtho.2021.07.017, PMID: 34352380

[236] Costa P Sales SLA Pinheiro DP Pontes LQ Maranhão SS Pessoa C . Epigenetic reprogramming in cancer: From diagnosis to treatment. Front Cell Dev Biol. (2023) 11:1116805. doi: 10.3389/fcell.2023.1116805, PMID: 36866275 PMC9974167

[237] Yang Q Chen Y Guo R Dai Y Tang L Zhao Y . Interaction of ncRNA and epigenetic modifications in gastric cancer: focus on histone modification. Front Oncol. (2021) 11:822745. doi: 10.3389/fonc.2021.822745, PMID: 35155211 PMC8826423

[238] Lima DG do Amaral G Planello AC Borgato GB Guimaraes GN de Souza AP . Combined therapy with cisplatin and 5-AZA-2CdR modifies methylation and expression of DNA repair genes in oral squamous cell carcinoma. Int J Clin Exp Pathology. (2022) 15:131–44., PMID: 35414841 PMC8986466

[239] de Castro LR de Oliveira LD Milan TM Eskenazi APE Bighetti-Trevisan RL de Almeida OGG . Up-regulation of TNF-alpha/NFkB/SIRT1 axis drives aggressiveness and cancer stem cells accumulation in chemoresistant oral squamous cell carcinoma. J Cell Physiol. (2024) 239:e31164. doi: 10.1002/jcp.31164, PMID: 38149816

[240] Banelli B Carra E Barbieri F Würth R Parodi F Pattarozzi A . The histone demethylase KDM5A is a key factor for the resistance to temozolomide in glioblastoma. Cell Cycle. (2015) 14:3418–29. doi: 10.1080/15384101.2015.1090063, PMID: 26566863 PMC4825557

[241] Sun J Cai X Yung MM Zhou W Li J Zhang Y . miR-137 mediates the functional link between c-Myc and EZH2 that regulates cisplatin resistance in ovarian cancer. Oncogene. (2019) 38:564–80. doi: 10.1038/s41388-018-0459-x, PMID: 30166592 PMC7474467

[242] Liu X Lu X Zhen F Jin S Yu T Zhu Q . LINC00665 induces acquired resistance to gefitinib through recruiting EZH2 and activating PI3K/AKT pathway in NSCLC. Mol Ther Nucleic Acids. (2019) 16:155–61. doi: 10.1016/j.omtn.2019.02.010, PMID: 30889481 PMC6424064

[243] Stief SM Hanneforth AL Weser S Mattes R Carlet M Liu WH . Loss of KDM6A confers drug resistance in acute myeloid leukemia. Leukemia. (2020) 34:50–62. doi: 10.1038/s41375-019-0497-6, PMID: 31201358 PMC7214274

[244] Yang C Zhang J Ma Y Wu C Cui W Wang L . Histone methyltransferase and drug resistance in cancers. J Exp Clin Cancer Res. (2020) 39:173. doi: 10.1186/s13046-020-01682-z, PMID: 32859239 PMC7455899

[245] Wang N Ma T Yu B . Targeting epigenetic regulators to overcome drug resistance in cancers. Signal Transduct Target Ther. (2023) 8:69. doi: 10.1038/s41392-023-01341-7, PMID: 36797239 PMC9935618

[246] Meng X Lou QY Yang WY Wang YR Chen R Wang L . The role of non-coding RNAs in drug resistance of oral squamous cell carcinoma and therapeutic potential. Cancer Commun (Lond). (2021) 41:981–1006. doi: 10.1002/cac2.12194, PMID: 34289530 PMC8504146

[247] Wu S Lv X Wei H Chen W Zheng J Li X . Circ-ILF2 in oral squamous cell carcinoma promotes cisplatin resistance and induces M2 polarization of macrophages. J Cell Mol Med. (2023) 27:4133–44. doi: 10.1111/jcmm.17998, PMID: 37864310 PMC10746935

[248] Chen S Yang M Wang C Ouyang Y Chen X Bai J . Forkhead box D1 promotes EMT and chemoresistance by upregulating lncRNA CYTOR in oral squamous cell carcinoma. Cancer Lett. (2021) 503:43–53. doi: 10.1016/j.canlet.2020.11.046, PMID: 33352248

[249] Xiao Z Li J Jin Q Liu D . Long non-coding RNA OIP5-AS1 contributes to cisplatin resistance of oral squamous cell carcinoma through the miR-27b-3p/TRIM14 axis. Exp Ther Med. (2021) 21:408. doi: 10.3892/etm.2021.9839, PMID: 33692839 PMC7938452

[250] Qiao X Liu J Zhu L Song R Zhong M Guo Y . Long noncoding RNA CEBPA-DT promotes cisplatin chemo-resistance through CEBPA/BCL2 mediated apoptosis in oral squamous cellular cancer. Int J Med Sci. (2021) 18:3728–37. doi: 10.7150/ijms.64253, PMID: 34790046 PMC8579301

[251] Gao L Zhang Q Li S Zheng J Ren W Zhi K . Circ-PKD2 promotes Atg13-mediated autophagy by inhibiting miR-646 to increase the sensitivity of cisplatin in oral squamous cell carcinomas. Cell Death Dis. (2022) 13:192. doi: 10.1038/s41419-021-04497-8, PMID: 35220397 PMC8882170

[252] Wenhao R Yali C Shaoming L Jingjing Z Ling G Keqian Z . circAP1M2 activates ATG9A-associated autophagy by inhibiting miR-1249-3p to promote cisplatin resistance in oral squamous cell carcinoma. J Cell Physiol. (2023) 238:2612–24. doi: 10.1002/jcp.31116, PMID: 37661341

[253] Gao F Han J Wang Y Jia L Luo W Zeng Y . Circ_0109291 promotes cisplatin resistance of oral squamous cell carcinoma by sponging miR-188-3p to increase ABCB1 expression. Cancer Biother Radiopharm. (2022) 37:233–45. doi: 10.1089/cbr.2020.3928, PMID: 32758011

[254] Liu T Chen G Sun D Lei M Li Y Zhou C . Exosomes containing miR-21 transfer the characteristic of cisplatin resistance by targeting PTEN and PDCD4 in oral squamous cell carcinoma. Acta Biochim Biophys Sin (Shanghai). (2017) 49:808–16. doi: 10.1093/abbs/gmx078, PMID: 28910982

[255] Li L Ye P Li G Xie T Zha C Wang Z . Carcinoma-associated fibroblast-derived exosomes lncRNA RORA-AS1 facilitates radiotherapy resistance of oral squamous cell carcinoma through the IFITM1/STAT axis. Biochim Biophys Acta Mol Cell Res. (2025) 1872:120015. doi: 10.1016/j.bbamcr.2025.120015, PMID: 40588135

[256] Zhang L Wang ZRY Liu KL Liu YH Wang S Jiang W . Targets of tumor microenvironment for potential drug development. Medcomm-Oncology. (2024) 3(1):44. doi: 10.1002/mog2.68

[257] Jin MZ Jin WL . The updated landscape of tumor microenvironment and drug repurposing. Signal Transduction Targeted Ther. (2020) 5(1):16. doi: 10.1038/s41392-020-00280-x, PMID: 32843638 PMC7447642

[258] Mitchell MJ Billingsley MM Haley RM Wechsler ME Peppas NA Langer R . Engineering precision nanoparticles for drug delivery. Nat Rev Drug Discov. (2021) 20:101–24. doi: 10.1038/s41573-020-0090-8, PMID: 33277608 PMC7717100

[259] Vatapalli R Rossi AP Chan HM Zhang J . Cancer epigenetic therapy: recent advances, challenges, and emerging opportunities. Epigenomics. (2025) 17:59–74. doi: 10.1080/17501911.2024.2430169, PMID: 39601374 PMC11702999

